# Electrodeposited bismuth nanomaterials as binder free and scalable catalysts for highly selective CO_2_ to formate conversion

**DOI:** 10.1039/d6ta04799h

**Published:** 2026-10-12

**Authors:** Anil Kumar Sihag, Yashraj Kumar Singh, Esraa Kotob, Sarah Zulfa Khairunnisa, Markus Ostermann, Olga Guselnikova, Markus Valtiner, Maryam Abdinejad, Christian M. Pichler

**Affiliations:** a Institute of Applied Physics, Vienna University of Technology 1040 Vienna Austria pichlerc@iap.tuwien.ac.at; b Indian Institute of Science Education and Research Pune 411008 India; c Department of Energy Conversion and Storage, Technical University of Denmark 2800 Lyngby Denmark

## Abstract

The design of binder-free catalyst materials that can be manufactured in a scalable manner and utilized in gas diffusion electrodes (GDEs) remains a critical bottleneck for further advancement of CO_2_-to-formate electrolysis. Here, direct chronopotentiometric electrodeposition from a choline chloride:ethylene glycol deep eutectic solvent (DES) was applied to manufacture conformal, nanowire-like bismuth catalyst layers on commercial GDE substrates (Bi/GDE). These materials demonstrate a significantly improved catalytic activity per mass, compared with the conventional aqueous deposition method, as they showed uncompromised catalytic activity at a 6-times lower mass loading. Insight into the electrochemical surface restructuring mechanism was gained by combining ICP-MS dissolution analysis with *in situ* Raman spectroscopy. It could be shown that both DES and aqueous-derived catalysts undergo an identical Bi_2_O_3_ → Bi_2_O_2_CO_3_ → Bi^0^ restructuring sequence, converging to a common hierarchical nanoflower active phase morphology with equivalent charge-transfer resistance, demonstrating that the activated state, not the initial morphology, governs selectivity. Both catalysts deliver high formate faradaic efficiencies at 100 mA cm^−2^ (96.8% for the DES- and 93.7% for the aqueous-derived electrode), with the DES electrode achieving a mass-normalised activity for formate production of 166.9 mA mg_Bi_^−1^. The electrodeposition process could be scaled to 150 cm^2^ substrates while maintaining a formate faradaic efficiency of 95.5%. Operation at 100 mA cm^−2^ is extended to 48 h of total elapsed time, corresponding to 32 h of cumulative electrolysis, through a periodic electrolysis swing protocol that mitigates GDE flooding, identified as the primary degradation pathway, while intrinsic catalyst stability showed no decline. This work establishes DES electrodeposition directly on GDEs as a material-efficient and inherently scalable route to high-performance Bi/GDE catalysts, and reveals electrochemical activation history as the key design parameter for CO_2_-to-formate conversion.

## Abstract 2

Binder free and scalable Bi-based GDE catalysts were prepared for electrochemical CO_2_ reduction. Different deposition electrolytes result in variations in the initial structure, which converge to a similar nanoflower morphology in the active state.

## Graphical abstract

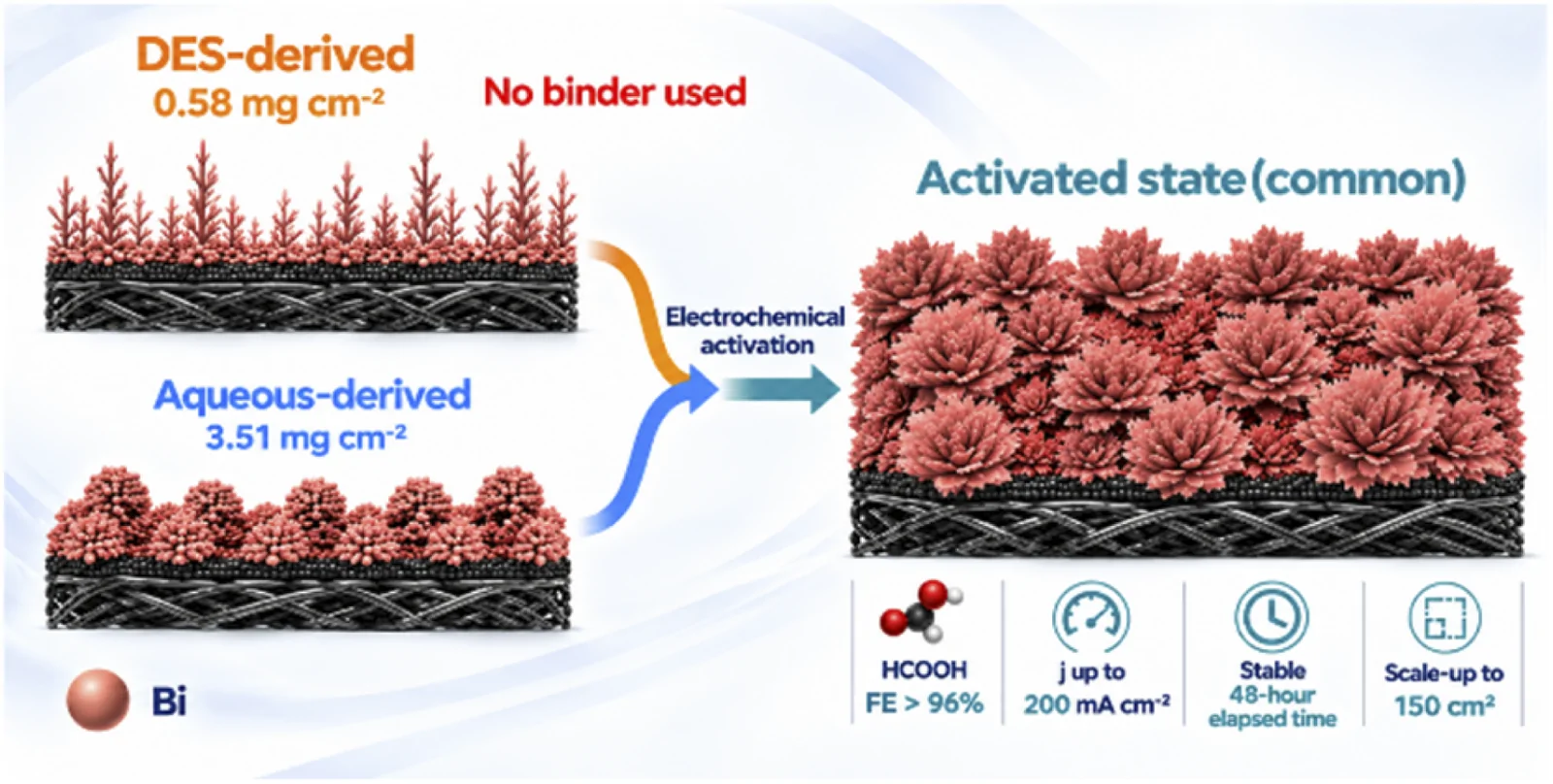

## 1 Introduction

The continuously increasing atmospheric concentration of carbon dioxide (CO_2_), driven primarily by anthropogenic fossil fuel combustion, necessitates the development of scalable carbon-neutral energy cycles.^1–3^ The electrochemical CO_2_ reduction reaction (CO_2_RR), powered by renewable electricity, has emerged as a promising strategy to convert CO_2_ into value-added chemicals and fuels while simultaneously enabling energy storage and carbon recycling.^4–7^

Catalyst composition critically governs product selectivity in CO_2_ electroreduction; Ag and Au predominantly yield CO,^8–10^ Cu-based catalysts enable the formation of multi-carbon (C2+) products,^11,12^ whereas post-transition metals such as Sn, In, and Bi selectively produce formic acid/formate.^13–19^ Among the various products, formic acid (HCOOH) is particularly attractive due to its simple two-electron pathway, which gives access to high faradaic efficiencies, and the molecule serves simultaneously as an energy storage vector, a commodity chemical, and a direct fuel for low-temperature fuel cells.^20–22^ Techno-economic analyses further identify CO_2_-to-formate as one of the most economically competitive CO_2_ reduction pathways and a realistic market entry route for CO_2_ electrolysis.^23–26^ The development of selective, stable, and scalable catalysts for this transformation therefore remains a central objective in electrochemical materials research.

In aqueous electrolytes, the hydrogen evolution reaction (HER) competes with the CO_2_RR at similar potentials, diverting electrons toward H_2_ and lowering faradaic efficiency of carbon products.^8^ Bismuth-based catalysts have demonstrated high formate faradaic efficiencies with effective suppression of the HER.^27–30^ Under cathodic operation, Bi-based precursors such as Bi_2_O_3_ and bismuth-based metal–organic frameworks (MOFs) are electrochemically reduced to metallic Bi, a transformation accompanied by pronounced morphological restructuring of the catalyst layer that establishes the active surface for CO_2_-to-formate conversion.^31–34^

Compositional tuning through doping with secondary metals such as In^35^ or Co^36^, or through main group and halide dopants such as B^37^ or F^38^ has been employed to modulate the Bi electronic structure and CO_2_ adsorption. Precursor engineering provides a complementary route: Bi-containing MOFs serve as structured precursors with high surface area and tunable porosity, while sulfate- and halide-based Bi compounds release anions during activation to generate porous, defective Bi surfaces.^38–42^ The extent to which the *in situ* formed catalyst state, rather than the initial as-deposited morphology or loading, controls selectivity and mass-normalised activity still remains poorly understood. Despite the advances in catalyst design, most reported materials are deposited onto the gas diffusion electrode (GDE) substrate by spray coating or drop casting from catalyst inks. The ink formulation typically requires fluorinated polymer binders (*e.g.*, Nafion and PTFE). Furthermore, the synthesis pathways of the catalyst materials are often very complex and require multiple individual steps. The combination of multi-step synthesis with a binder-based formulation limits the cost-effectiveness and scalability of catalyst manufacturing.^43^ This is a major obstacle for implementing these catalyst materials in larger scale applications and hampering the advancement of the CO_2_RR in general.

In this study, a facile and scalable chronopotentiometric electrodeposition approach is reported for depositing binder-free Bi based catalyst layers directly onto commercial GDE substrates from two different electrolyte systems (deep eutectic solvent/DES and aqueous system), yielding catalysts with markedly different morphologies. Mechanistic insights into dynamic restructuring of these nanostructures under electrocatalytic conditions were obtained by coupling inductively coupled plasma mass spectrometry (ICP-MS) dissolution analysis with *in situ* Raman spectroscopy. The influence of both the initial and electrochemically activated morphology on CO_2_RR performance and long-term stability was then systematically investigated, addressing a central research gap in the field. The DES-derived Bi/GDE achieves a faradaic efficiency of 96.8% toward formate at 100 mA cm^−2^ (−2.4 V *vs.* Ag/AgCl) at a Bi loading of only 0.58 mg cm^−2^, corresponding to a mass-normalised partial current density of 166.9 mA mg^−1^, approximately 6-fold higher than that of the aqueous-derived counterpart (26.7 mA mg^−1^) at equivalent selectivity. At 150 mA cm^−2^, the aqueous-derived Bi/GDE, owing to its larger Bi reservoir (3.5 mg cm^−2^), retains a formate faradaic efficiency (FE) of 88.9%, whereas the DES-derived catalyst becomes Bi-reservoir limited. The electrodeposition approach was subsequently demonstrated on substrates of up to 150 cm^2^, confirming the scalability of the deposition process. The DES-derived Bi/GDE was selected for long-term electrolysis tests, given its uniform conformal coverage on large-area substrates and its 6-fold lower Bi loading, both of which are practically advantageous for scaled deployment. Extended operation was demonstrated using a periodic electrolysis swing protocol, which extended the experiment to 48 h of total elapsed time, corresponding to 32 h of cumulative electrolysis, while maintaining a formate faradaic efficiency of 85% in a GDE flow cell.

By integrating scalable materials synthesis, mechanistic understanding of dynamic restructuring, and improved catalytic operation protocols, this work advances the rational design of Bi-based catalysts for industrially relevant CO_2_-to-formate conversion.

## 2 Results and discussion

### 2.1 Electrode preparation, morphology, and structural characterization

Bi/GDEs were fabricated by direct chronopotentiometric electrodeposition onto commercial GDEs from either an acidic, aqueous electrolyte or a choline chloride:ethylene glycol deep eutectic solvent (DES), using a two-electrode configuration (Fig. 1a and b). The deposition current density was varied between 2 and 36 mA cm^−2^ to systematically control the Bi loading and morphology. The electrodeposition synthesis approach was subsequently demonstrated on large-area substrates up to 10 × 15 cm^2^, confirming the scalability of the deposition process.

**Fig. 1:**
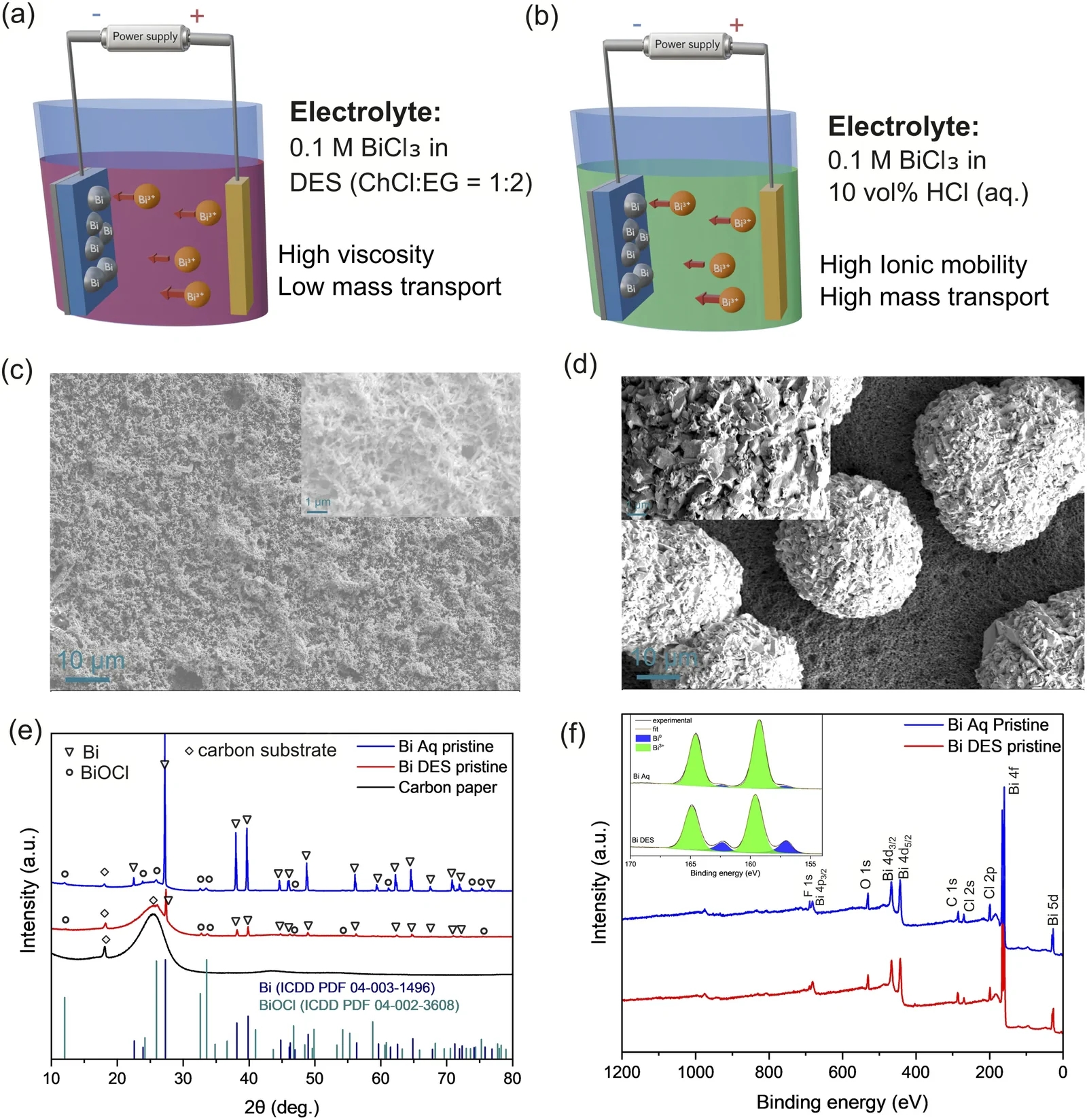
Schematic illustration of the two-electrode electrodeposition setup used for Bi deposition onto GDEs (a) from DES, and (b) from aqueous solvent, (c and d) SEM images of pristine Bi catalysts electrodeposited from (c) DES and (d) aqueous electrolyte, with higher-magnification insets, (e) XRD patterns of aqueous- and DES-derived pristine Bi electrodes compared to a bare GDE (carbon paper), with reference diffractograms for metallic Bi (ICDD PDF 04-003-1496) and BiOCl (ICDD PDF 04-002-3608), and (f) XPS survey spectra of pristine aqueous- and DES-derived Bi electrodes, with high-resolution Bi 4f spectra shown in the inset, confirming the presence of Bi, O, C, Cl, and F (from the PTFE-treated GDE substrate).

The mass of electrodeposited Bi was quantified by ICP-MS after acid digestion and normalized to the geometric electrode area (Table 1).

**Table 1:** Bismuth loading and electrodeposition efficiency for DES and aqueous electrolytes at different current densities. Electrode area: 2 cm^2^; digest volume: 20 mL

Sample	j (mA cm^−2^)	[Bi] (mg L^−1^)	Bi mass (mg)	Exp. loading (mg cm^−2^)	Theor. loading (mg cm^−2^)	Efficiency (%)
DES	2	22.47	0.449	0.224	0.73	31.00
DES	4	58.12	1.162	0.581	1.45	40.08
DES	18	55.99	1.119	0.559	6.50	8.61
Aqueous	2	42.44	0.848	0.424	0.73	58.55
Aqueous	4	88.27	1.765	0.882	1.45	60.88
Aqueous	18	351.12	7.022	3.511	6.50	54.02

At 4 mA cm^−2^, the DES system produced an experimental Bi loading of 0.58 mg cm^−2^, corresponding to a faradaic efficiency of 40% relative to the theoretical Faraday's law estimation. Increasing the DES deposition current to 18 mA cm^−2^ yielded a nearly identical loading of 0.56 mg cm^−2^ corresponding to a drastically reduced efficiency of only 8.6%. This indicates that the deposition process becomes strongly mass-transport limited at higher current densities in the viscous DES medium.^44^ In contrast, aqueous electrodeposition at 18 mA cm^−2^ produced a substantially higher Bi loading of 3.51 mg cm^−2^ at 54% efficiency, consistent with the significantly lower viscosity and faster Bi^3+^ mass transport in the aqueous system.^44^ These results establish that DES electrodeposition operates in a fundamentally different mass-transport regime. This leads to an intrinsic upper limit on the achievable Bi loading that is independent of the applied current density above a threshold value and results in approximately 6-times lower Bi loading than aqueous electrodeposition at the same current density.

The morphological consequences of these different deposition environments are clearly revealed by scanning electron microscopy (Fig. 1c, d and S1 (SI)). The aqueous-deposited catalyst forms discrete, compact spherical Bi aggregates distributed across the GDE surface, with individual particle diameters of tens of micrometers as seen at low magnification (Fig. S1a and c). At higher magnification, the surfaces of these spheres consist of densely packed, faceted crystalline domains (Fig. S1e). The DES-deposited catalyst, in contrast, produces a continuous, uniform, nanostructured coating, with a wire-like morphology, that covers the carbon fibers with nanoscale Bi domains and interconnected filament-like features, leaving the GDE porosity largely intact (Fig. 1c, S1b, d and f). This fundamental difference in morphology, localized spherical aggregates *versus* a continuous conformal coating, reflects the distinct nucleation and growth kinetics imposed by the two electrolyte environments and can have direct implications for catalyst stability and GDE functionality.

Energy-dispersive X-ray spectroscopy (EDX) elemental mapping confirms the spatial distribution of Bi in both catalyst types (Fig. S2). For the DES-derived electrode (Fig. S2a), Bi is distributed relatively uniformly across the GDE surface, while chlorine (Cl) is detected throughout the deposit, originating from the choline chloride component of the DES electrolyte. The aqueous-deposited catalyst (Fig. S2b) shows Bi confined to the large spherical aggregates, with the O signal overlapping strongly with the Bi-rich regions, consistent with a surface Bi-oxide layer. In both cases, carbon and fluorine signals arise from the GDE substrate and its PTFE-based hydrophobic coating, respectively.

X-ray diffraction (XRD) analysis of the pristine electrodes (Fig. 1e) reveals that both catalysts exhibit reflections corresponding to rhombohedral metallic bismuth (Bi, ICDD PDF 04003-1496), with characteristic (012), (104), and (110) reflections at 2*θ* = 27.22°, 38.02°, and 39.68° for the aqueous-derived catalyst and 27.40°, 38.19°, and 39.85° for the DES-derived catalyst. Additional reflections attributable to bismuth oxychloride (BiOCl, ICDD PDF 04-002-3608) are observed in both patterns, consistent with the BiCl_3_ precursor common to both deposition baths and with the residual chloride detected by EDX and XPS. Scherrer analysis of the three Bi reflections yields average crystallite sizes of 56.6 nm (aqueous) and 52.5 nm (DES) (Table S4), confirming that the significantly weaker and broader peak intensities of the DES pattern reflect its substantially lower Bi mass loading (0.58 *vs.* 3.51 mg cm^−2^) rather than a meaningful reduction in crystallite size, consistent with the conformal but lower-density Bi coverage observed by SEM. The broad feature near 26° and the sharp reflection near 18° originate from the carbon paper substrate and its PTFE hydrophobic treatment, respectively, and are reproduced in the pattern of the bare GDE (Fig. 1e, black trace). All observed reflections could be assigned to metallic Bi, BiOCl, or the substrate; no additional crystalline phases were detected.

X-ray photoelectron spectroscopy (XPS) survey spectra of the pristine electrodes show Bi, O, C, and Cl in both samples, together with F originating from the PTFE-treated GDE substrate (Fig. 1f). High-resolution Bi 4f spectra (inset of Fig. 1f and S6) show that the surface of both pristine catalysts is dominated by Bi^3+^ (Bi 4f_7/2_ at 159.3–159.6 eV), with a minor metallic Bi^0^ contribution at 156.8–157.0 eV. The Bi^0^ fraction is higher for the DES-derived catalyst (20%) than for the aqueous-derived catalyst (5%) (Table S5), suggesting that the low water content and reducing character of the ethylene glycol-based DES partially suppress surface oxidation during deposition. Consistently, the O 1s spectra of both pristine catalysts are dominated by Bi_2_O_3_ lattice oxygen (∼530 eV), with a smaller Bi–OH contribution (∼531.3 eV) (Fig. S7). The C 1s spectrum of the pristine DES-derived catalyst additionally shows a pronounced C–O component at 286.5 eV, attributed to residual choline chloride/ethylene glycol from the deposition bath (Fig. S8), and the Cl 2p_3/2_ signal at 198.1–198.4 eV corresponds to Bi-bound chloride, in agreement with the BiOCl reflections observed by XRD. The predominance of oxidized Bi in XPS, in contrast to the metallic Bi dominating the XRD patterns, reflects the different probing depths of the two techniques: XRD samples the crystalline bulk, whereas XPS probes only the outermost few nanometres, where a thin oxide/hydroxide layer forms upon air exposure of the *ex situ* samples.

Comparing the DES and aqueous deposited materials, their distinctive difference is their varying morphology (nanowires in DES *vs.* spherical in aqueous) and that in DES materials a coherent Bi covering could be achieved with reduced mass loading (0.58 *vs.* 3.5 mg cm^−2^).

### 2.2 Electrochemical activation, catalyst restructuring and effects on stability

Prior to the CO_2_RR, both catalyst types were activated by cyclic voltammetry (CV) in CO_2_-saturated 0.5 M KHCO_3_ as catholyte in the flow cell setup. The DES-derived catalyst in its pristine state exhibits a nanofibrous and interconnected nanosheet morphology (Fig. 2a), while the aqueous-derived catalyst shows large compact spherical aggregates (Fig. 2d). The CV profiles recorded during activation over 16 cycles are shown in Fig. 2b and e for the DES- and aqueous-derived catalysts, respectively. In the DES case, the cathodic current evolves and stabilizes relatively quickly, with successive cycles converging within the first 10 cycles. The aqueous catalyst undergoes more pronounced evolution during the early cycles: the initial sweeps show large cathodic excursions reaching beyond −75 mA cm^−2^ that diminish substantially upon subsequent cycling, reflecting significant dissolution and redistribution of the loosely bound, high-loading Bi spheres during the initial activation steps. In both cases, a broad anodic feature near −0.3 to −0.5 V *vs.* Ag/AgCl and a corresponding cathodic reduction peak are observed, consistent with the reversible Bi^0^/Bi^3+^ redox couple in bicarbonate electrolyte. The progressive stabilization of this feature with cycle number indicates that the dissolution–redeposition dynamics reach a steady state after 16 cycles for both materials, ensuring stable catalyst morphology. Therefore, 16 cycles were used as the standard activation protocol before subjecting the samples to the CO_2_RR.

**Fig. 2:**
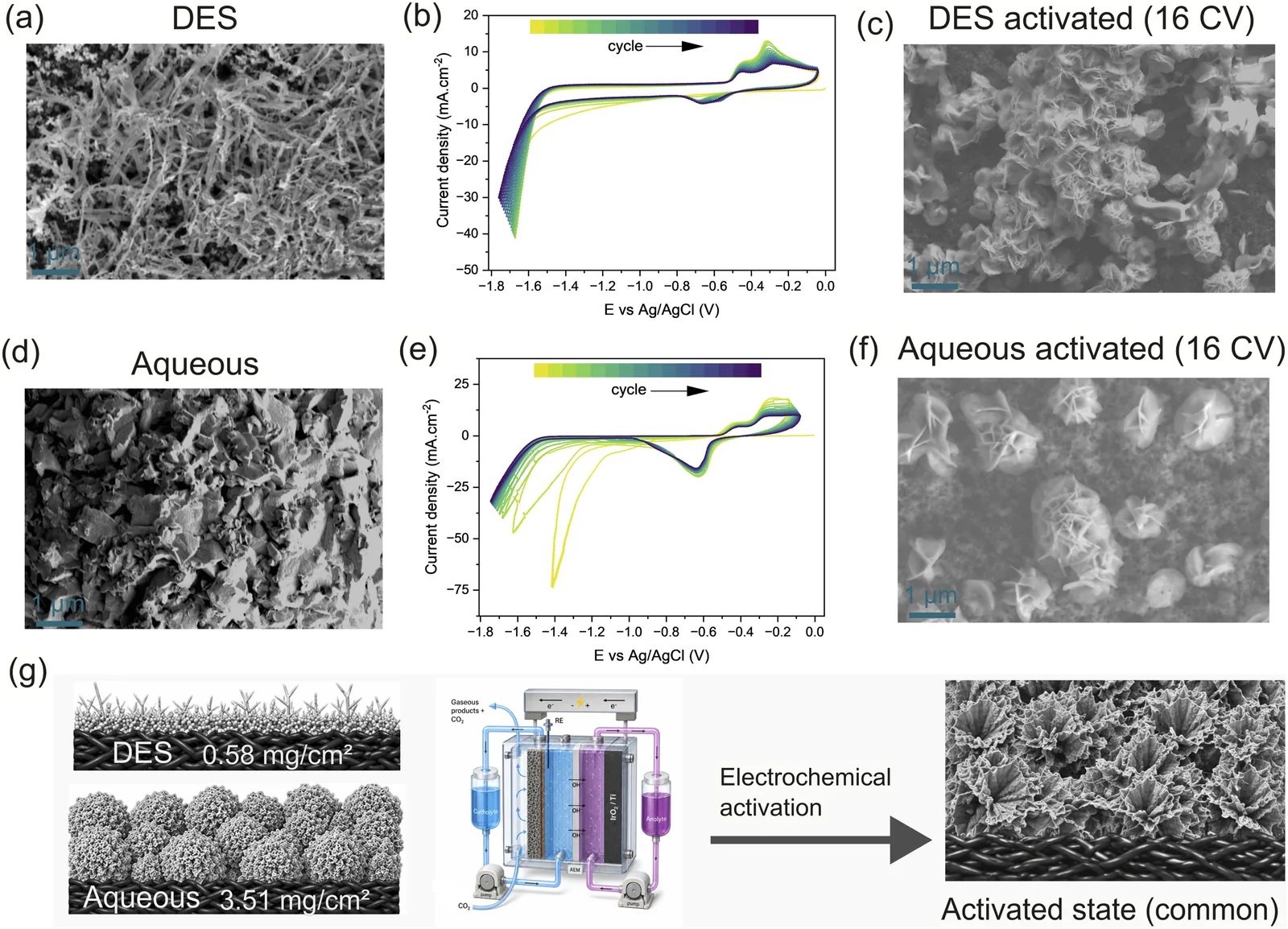
Electrochemical activation and morphological restructuring of Bi catalysts. (a and d) SEM images of pristine Bi catalysts electrodeposited from (a) DES and (d) aqueous electrolyte. (b and e) Cyclic voltammograms recorded during 16 activation cycles for (b) DES-derived and (e) aqueous-derived catalysts; the color gradient from yellow to purple indicates increasing cycle number. (c and f) SEM images after 16 CV activation cycles for (c) DES-derived and (f) aqueous-derived catalysts, revealing the formation of hierarchical nanoflower-like Bi structures in both cases. Scale bars: 1 µm. (g) Schematic illustration of the electrochemical activation process performed in the flow cell setup using 0.5 M KHCO_3_ as catholyte: both DES-derived and aqueous-derived catalysts, despite their distinct initial morphologies, converge to a common activated nanoflower state following electrochemical activation.

SEM analysis after 16 CV cycles reveals that both catalysts have undergone substantial morphological transformation, converging toward a common nanoflower-like architecture regardless of their initial structure (Fig. 2c and f). The previously compact DES derived nanofibers and the large aqueous derived spheres have both evolved into porous assemblies of thin, curved Bi nanosheets, forming hierarchical flower-like clusters. This morphological convergence is attributed to a dissolution–redeposition mechanism operative during CV cycling: partial oxidation of Bi to soluble Bi^3+^ during the anodic sweep, followed by diffusion-controlled redeposition during the cathodic sweep, progressively erases the structural memory of the initial deposit and drives recrystallization toward a thermodynamically preferred branched nanosheet morphology.^31,40^ A schematic summary of the activation-driven convergence to the common nanoflower phase is shown in Fig. 2g.

SEM images after CO_2_RR operation (Fig. S3) show morphologies essentially identical to those observed after CV activation, demonstrating that the major structural transformation occurs during the activation step and that the nanoflower structure is preserved under CO_2_RR conditions. This observation implies that the active catalytic state is generated during activation, not during electrolysis, and that the nanoflower morphology represents the stable working structure of the catalyst.

XRD after 16 CV cycles and after the CO_2_RR (Fig. S4) confirms that metallic Bi remains the only crystalline Bi phase in both systems, with weak BiOCl reflections persisting throughout. Additional reflections of KHCO_3_ (ICDD PDF 04-002-2529) dominate the electrolysed samples and provide direct crystallographic evidence for the carbonate precipitation identified below as the primary degradation mechanism. The weaker Bi reflections of the DES electrodes reflect their lower loading rather than loss of the metallic phase. Although weak BiOCl reflections persist in the bulk, XPS shows that chloride is largely removed from the catalyst surface during activation (see below), indicating that residual chloride does not govern the restructuring pathway.

XPS of the activated (16 CV) and post-CO_2_RR (100CD) electrodes (Fig. S6–S9 and Table S5) shows that the surface chemistry of both catalysts converges upon activation. With the binding-energy scale referenced to the Fermi edge, the Bi 4f_7/2_ positions of Bi^0^ (156.8–157.0 eV) and Bi^3+^ (159.1–159.6 eV) remain essentially unchanged across the pristine, activated, and post-CO_2_RR states. The Bi^0^ fraction decreases for the DES-derived catalyst (20% → 13% → 8%) and increases for the aqueous-derived catalyst (5% → 11% → 10%), converging to a comparable Bi^0^/Bi^3+^ balance. As the measurements were performed *ex situ*, part of the Bi^0^ formed under cathodic conditions is re-oxidized upon air exposure. In the O 1s spectra, the Bi–OH component becomes dominant for both catalysts after activation (69–78% of the Bi-associated oxygen) and is retained after the CO_2_RR, while K 2p and O–C=O (∼289 eV) contributions appear in the C 1s region, consistent with residual KHCO_3_ and surface carbonate species. REELS spectra (Fig. S5) follow the same trend: the pristine DES-derived catalyst exhibits a distinctly higher plasmon-loss intensity in the 10–20 eV region, consistent with its larger Bi^0^ fraction, whereas all activated and post-CO_2_RR samples show comparable spectral features. The differences in the surface electronic structure present in the as-deposited catalysts are therefore erased during activation, and no DES-specific electronic effect persists in the working catalyst.

Surface chloride decreases from 12.9 and 17.2 at% in the pristine DES- and aqueous-derived catalysts to ≤3.4 at% after activation and ≤1.9 at% after the CO_2_RR (Fig. S9). To verify that this chloride is a residue of the deposition electrolyte (BiCl_3_ and, for DES, choline chloride), pristine electrodes were assembled in the flow cell and flushed with DI water for 3 h without applied potential. This treatment removed surface chloride completely from the DES-derived electrode and reduced it to 3.7 at% on the aqueous-derived electrode, consistent with part of the chloride being bound in BiOCl. Residual chloride is thus a leachable deposition residue rather than a constituent of the activated catalyst surface.

The stability of different catalyst morphologies under electrochemical operation was quantified by ICP-MS dissolution analysis (Table S3). Direct chronopotentiometric CO_2_RR operation without prior activation (CP20) led to substantial Bi leaching from the DES electrode, with 21.6% of the initial loading dissolved, confirming that the as-deposited nanowire morphology is mechanically unstable and undergoes significant uncontrolled restructuring upon initial electrochemical exposure. The aqueous-derived Aq-CP20 electrode lost only 1.15% under identical conditions, consistent with its more strongly anchored compact Bi aggregates. However, this lower dissolution percentage does not imply that CV activation is unnecessary for the aqueous system. It is well established that Bi-based precatalysts undergo an intrinsic restructuring into nanosheet-type active phases upon exposure to bicarbonate electrolyte under cathodic polarisation, irrespective of the initial morphology.^45^ Without prior CV activation, this restructuring occurs in an uncontrolled manner during the CO_2_RR, generating transient morphological changes and dissolution events that compromise initial selectivity and operational stability.

Following 16 CV activation cycles, Bi dissolution in the DES system is reduced to 0.46 mg L^−1^ (2.1% of the initial loading), and remains stable at 2.9% even after subsequent chronopotentiometry at 100 mA cm^−2^ for 4500 s (DES-100CD). The restructured nanoflower phase is known to exhibit markedly lower dissolution rates than precursor Bi morphologies, as the dissolution–redeposition process during CV cycling produces a thermodynamically more stable surface configuration with stronger substrate anchoring.^31,45^ The aqueous-derived catalyst shows consistently low dissolution across all conditions (1.2–2.0% of the initial loading). While the relative percentage loss of the aqueous electrode appears smaller, this reflects its 6-fold larger Bi reservoir: the absolute dissolved Bi per unit area after activation is 0.012 mg cm^−2^ for DES-16 CV *versus* 0.070 mg cm^−2^ for Aq-16 CV, indicating that the DES system achieves comparable intrinsic dissolution resistance per unit area despite its substantially lower loading.

### 2.3 CO_2_ electroreduction performance in a flow cell

Having established the activation and dissolution behavior, the activated DES- and aqueous-derived Bi/GDEs were evaluated in the CO_2_RR under flow-cell conditions. The CO_2_ electroreduction performance of the activated Bi/GDEs was evaluated in a flow-cell configuration using an AEM and an IrO_2_/Ti anode, with CO_2_-saturated 0.5 M KHCO_3_ as the catholyte (Fig. 3a). Prior to evaluating current density-dependent CO_2_RR performance, the influence of electrodeposition conditions on catalytic selectivity was assessed (Fig. S11). Bi/GDEs prepared at different deposition current densities were tested at 100 mA cm^−2^. For DES-derived electrodes, high formate selectivity is achieved over a broad deposition window, with FE_HCOOH_ exceeding 90% for electrodes deposited at 4–18 mA cm^−2^ (Bi loading of 0.58 and 0.56 mg cm^−2^, respectively; Table 1), while the lower deposition current density of 2 mA cm^−2^ (0.22 mg cm^−2^) leads to negligible activity. In contrast, aqueous-derived electrodes show a strong dependence on deposition current density, with FE_HCOOH_ increasing from 13.3% at 2 mA cm^−2^ (0.42 mg cm^−2^) to 93.7% at 18 mA cm^−2^ (3.51 mg cm^−2^). Normalising these faradaic efficiencies by the corresponding Bi loading (Fig. S12) shows that the DES-derived catalyst reaches a mass-normalised HCOOH partial current density of ∼166 mA mg_Bi_^−1^ already at 4 mA cm^−2^ deposition current and sustains this level up to 18 mA cm^−2^, despite an essentially unchanged Bi loading over this range, whereas the aqueous-derived catalyst does not exceed 37 mA mg_Bi_^−1^ at any point across the deposition series. This confirms that the higher mass-normalised activity of the DES catalyst is a consistent feature of its low-loading regime. This performance dependence mirrors the EIS data (Fig. S10), where the aqueous-2 and aqueous-4 mA cm^−2^ electrodes exhibit prohibitively high charge-transfer resistances of 160.9 and 27.08 Ω, respectively, while aqueous-18 mA cm^−2^ and DES-4 mA cm^−2^ converge to equivalent *R*_ct_ values of 12.28 and 13.16 Ω despite their 6-fold difference in Bi loading. Electrodes deposited at 18 mA cm^−2^ (aqueous) and 4 mA cm^−2^ (DES) were therefore selected as representative catalysts for CO_2_RR studies. Cyclic voltammograms recorded under CO_2_ flow (Fig. 3b) show comparable electrochemical behavior for both catalysts, with an onset near −0.55 V *vs.* Ag/AgCl and increasing cathodic current at more negative potentials. The aqueous-derived electrode exhibits a slightly more pronounced anodic feature at around −0.4 V, indicative of a clearer Bi^0^/Bi^3+^ redox response, while the DES-derived electrode displays marginally higher currents at the most negative potentials, consistent with its lower charge-transfer resistance at equivalent loading as determined by EIS (Fig. S10).

**Fig. 3:**
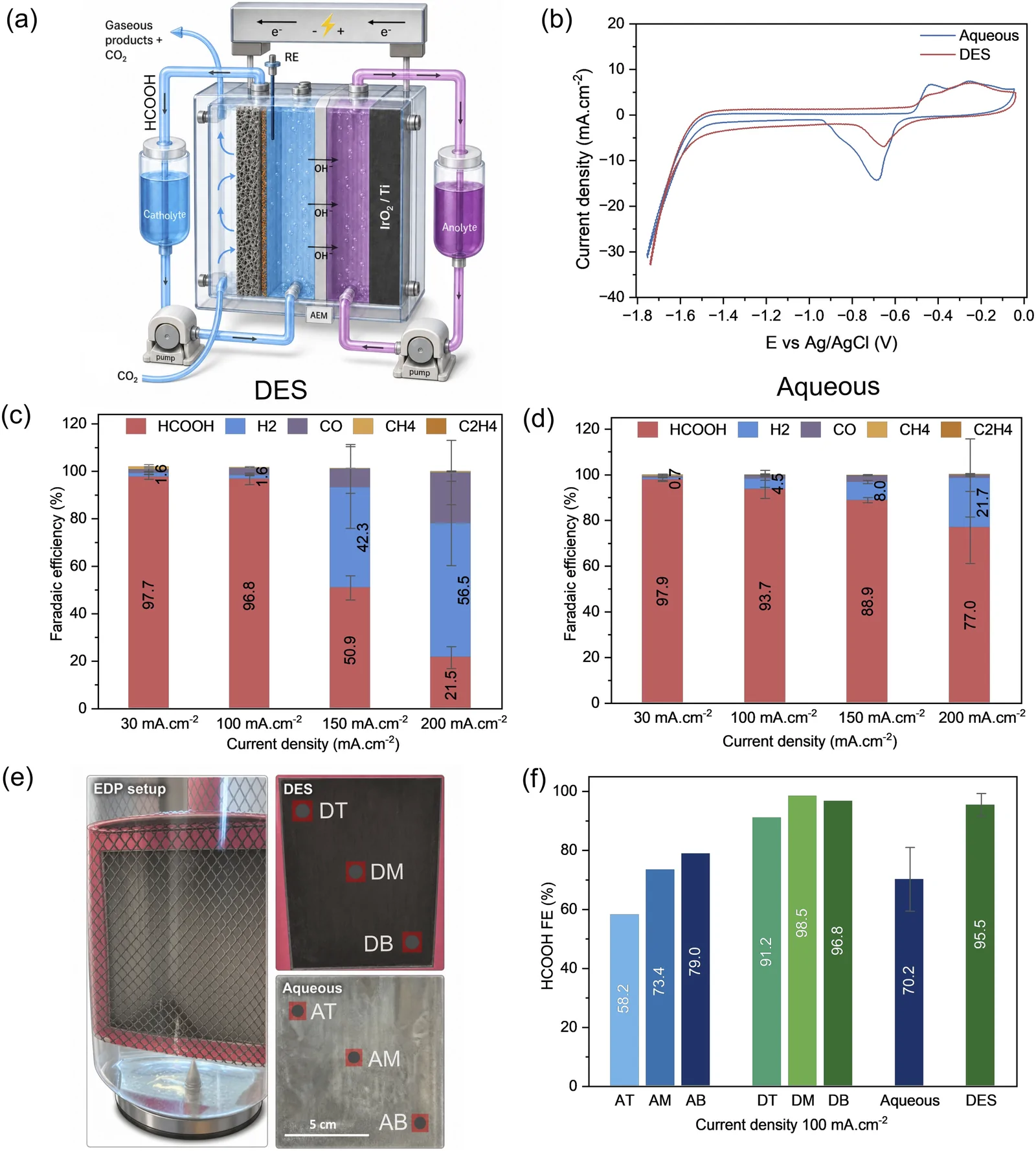
CO_2_ electroreduction performance of Bi/GDE catalysts in a flow-cell configuration. (a) Schematic of the flow-cell setup comprising a Bi/GDE (2 × 1 cm^2^) cathode, IrO_2_/Ti anode, anion exchange membrane (AEM), and recirculating KHCO_3_ catholyte and KOH anolyte. (b) Cyclic voltammograms of DES-derived and aqueous-derived Bi/GDE catalysts recorded under CO_2_ flow. (c and d) Faradaic efficiency distributions for HCOOH, H_2_, CO, CH_4_, and C_2_H_4_ at current densities of 30–200 mA cm^−2^ for (c) DES-derived and (d) aqueous-derived Bi/GDE catalysts. (e) Photograph of the electrodeposition (EDP) setup and of the resulting large-area Bi deposits on GDE substrates (10 × 15 cm^2^ for DES and 10 × 10 cm^2^ for aqueous deposition), obtained using a stainless-steel mesh counter electrode with a geometric area approximately 1.1× that of the working electrode. Markers indicate the three positions, top (T), middle (M), and bottom (B), sampled from each large-area electrode for catalytic testing, denoted as DT, DM, and DB for the DES-derived electrode and AT, AM, and AB for the aqueous-derived electrode. Scale bar: 5 cm. (f) Faradaic efficiency toward formate at 100 mA cm^−2^ measured individually at each sampling position of the aqueous-derived (AT, AM, and AB) and DES-derived (DT, DM, and DB) large-area electrodes, together with the corresponding position-averaged values (aqueous and DES); error bars denote the standard deviation over the three positions.

Product distribution was quantified by GC and HPLC over a current density range of 30–200 mA cm^−2^ (Fig. 3c and d). Both catalysts demonstrate high formate selectivity at moderate current densities. The DES-derived catalyst achieves an FE_HCOOH_ of 97.7% at 30 mA cm^−2^ and retains 96.8% at 100 mA cm^−2^, with minor H_2_ contributions of 1.6% at both current densities, placing them among the best reported catalysts concerning selectivity.^27,46,47^ The aqueous-derived catalyst similarly delivers 97.9% at 30 mA cm^−2^ and 93.7% at 100 mA cm^−2^. This performance equivalence at moderate current densities is a direct consequence of the morphological restructuring described above: CV activation drives both systems toward the same nanoflower active structure with equivalent *R*_ct_, resulting in identical CO_2_RR selectivity within the operating range where the activated Bi surface is stable.

At elevated current densities, a pronounced divergence in selectivity emerges. The DES-derived catalyst undergoes a sharp decline in FE_HCOOH_ to 50.9% at 150 mA cm^−2^, accompanied by a significant increase in H_2_ evolution (42.3%), and at 200 mA cm^−2^ H_2_ becomes the dominant product alongside significant CO formation (FE_H2_: 56.5%, FE_HCOOH_: 21.5%, FE_CO_: 21.4%). The aqueous-derived catalyst, in contrast, shows substantially higher formic acid selectivity: 88.9% at 150 mA cm^−2^ (H_2_: 8.0%) and 77.0% at 200 mA cm^−2^ (H_2_: 21.7%). The mass-normalised HCOOH partial current density quantifies this trade-off directly. At 100 mA cm^−2^, the DES-derived catalyst achieves 166.9 mA mg_Bi_^−1^, approximately 6-fold higher than the aqueous-derived catalyst (26.7 mA mg_Bi_^−1^), confirming a 6-fold higher Bi utilisation efficiency within the moderate current density regime. Above 100 mA cm^−2^, however, the mass-normalised activity of the DES catalyst declines sharply to 131.6 and 74.1 mA mg_Bi_^−1^ at 150 and 200 mA cm^−2^, respectively, while that of the aqueous catalyst continues to increase, reaching 43.9 mA mg_Bi_^−1^ at 200 mA cm^−2^. A comparison with Bi-based catalysts in the literature is shown in Table S6. Under identical flow cell conditions, an air-brushed electrode prepared from commercial Bi powder at a comparable loading (0.5 mg cm^−2^) reached a formate faradaic efficiency of only 6.2% at 100 mA cm^−2^, with H_2_ as the main product (Fig. S13 and Table S6). This inversion directly reflects loading-dependent active-site loss: the ICP-MS dissolution data confirm that unactivated DES-derived Bi undergoes significant leaching (∼21.6% of the initial loading) under direct chronopotentiometric operation, and while CV activation reduces this to ∼2–3%, the absolute Bi reservoir of the DES electrode (0.58 mg cm^−2^) remains intrinsically limited. At current densities exceeding 100 mA cm^−2^, where cathodic overpotentials exceed −2.35 V *vs.* Ag/AgCl and vigorous H_2_ bubble evolution disrupts the triple-phase boundary, this limited Bi reservoir is insufficient to sustain the active surface area required for selective CO_2_RR, shifting selectivity toward the HER. The aqueous electrode, carrying 6-fold greater Bi mass, retains productive active site density under equivalent conditions.^31,48^

Chronopotentiometric measurements at fixed current densities (Fig. S14) confirm the stable electrochemical behavior of both catalysts over the measurement period (∼4500 s). The DES-derived electrode operates at stable potentials of approximately −1.85 V and −2.35 V *vs.* Ag/AgCl at 30 and 100 mA cm^−2^, respectively, with increasingly noisy profiles at 150 and 200 mA cm^−2^ consistent with gas bubble dynamics at the GDE surface. The aqueous catalyst spans a broader potential range, reaching beyond −4.5 V at 300 mA cm^−2^, and while stable at 30 mA cm^−2^, shows significant potential fluctuations and a gradual positive drift at 100–300 mA cm^−2^, indicative of progressive changes at the triple-phase boundary under prolonged high-current operation.

### 2.4 Scalability of the electrodeposition process

After comparing different material structures and gaining insight into their restructuring mechanisms, the next step is to select an optimal material for long-term tests. Alongside catalytic performance, large-area uniformity is a critical requirement for practical deployment of GDE-based CO_2_ electrolysis: the ability to fabricate electrodes over large areas with uniform catalyst coverage and reproducible catalytic performance will serve as the main criterion for catalyst selection. To evaluate this, Bi was deposited on GDEs with areas of 10 × 15 cm (DES) and 10 × 10 cm (aqueous) using the same electrodeposition protocol as the laboratory scale electrodes (Fig. 3e). To assess the spatial homogeneity of the deposits, three samples of 2 × 1 cm^2^ were cut from the top, middle, and bottom of each large-area electrode and tested independently at 100 mA cm^−2^ under otherwise identical flow-cell conditions. The DES-derived electrode delivered formic acid faradaic efficiencies of 91.2%, 98.5%, and 96.8% at the top, middle, and bottom positions, respectively, corresponding to a position-averaged value of 95.5 ± 3.8%. The aqueous-derived electrode gave 58.2%, 73.4%, and 79.0% at the corresponding positions, averaging 70.2 ± 10.8% (Fig. 3f). SEM images recorded at the three sampling positions (Fig. S15) support this interpretation: the DES-derived electrode exhibits a continuous and homogeneous nanostructured Bi coating at all positions, whereas the aqueous-derived electrode shows a pronounced gradient in Bi coverage, from sparsely distributed small aggregates at the top to densely packed, large spherical aggregates at the bottom, mirroring the positional increase in formate selectivity. The threefold smaller standard deviation of the DES-derived electrode quantifies this uniformity directly. The aqueous-derived electrode additionally shows a monotonic increase in selectivity from the top to the bottom of the deposit. This positional trend is consistent with partial peeling of the comparatively thick aqueous Bi film, which was most pronounced in the upper region of the electrode, and with the weak substrate adhesion of the large aqueous Bi aggregates already inferred from their pronounced dissolution and redistribution during CV activation (Section 2.2). A non-uniform current distribution across the large-area substrate may contribute additionally. No comparable positional trend is observed for the DES-derived electrode, whose thin conformal layer remained intact across the full deposit area. The substantially higher and more uniform selectivity of the DES-derived large-area electrode is attributed to the conformal and homogeneous Bi coating produced by DES electrodeposition, which ensures consistent catalyst coverage across the electrode surface without the large aggregate clustering and non-uniform distribution that characterize aqueous deposition at scale. These results demonstrate that DES electrodeposition yields more uniform large-area formate selectivity at scale, establishing it as a practically viable fabrication strategy for large-area Bi/GDEs in CO_2_ electrolysis systems. Therefore, the DES material was chosen for the subsequent long-term CO_2_RR tests.

### 2.5 *In Situ* Raman analysis of activation-driven restructuring and the reaction pathway

*In situ* Raman spectroscopy was employed to probe the potential dependent evolution of the catalyst structure and surface intermediates under CO_2_ reduction conditions. Measurements were performed between open circuit potential (OCP) and −1.8 V *vs.* Ag/AgCl (corresponding to approximately +0.64 to −1.16 V *vs.* RHE) for all four catalyst states: aqueous-derived before activation (Aq), aqueous-derived after 16 CV activation cycles (Aq act), DES-derived before activation (DES), and DES-derived after activation (DES act). While dissolved formate is difficult to detect due to its low Raman cross-section, surface-bound intermediates can be identified *via* their potential-dependent spectral behavior.^49,50^ To ensure that all observed spectral features originate from the catalyst and reaction environment, background measurements were performed using electrolyte-only and electrolyte with carbon paper (Fig. S17). No characteristic bands were observed in the low-frequency region (100–200 cm^−1^) or in the carbonate region (1000–1100 cm^−1^), confirming that the signals discussed below arise exclusively from Bi-based catalyst phases and adsorbed species. However, intense signals in the 1300–1600 cm^−1^ range, where potentially adsorbed ^*^OCHO intermediates (1470 cm^−1^) could appear, overlap substantially with the broad D/G envelope of the carbon-paper support and were therefore excluded from mechanistic analysis, as discussed below.

Two potential-dependent spectral regions (50–300 cm^−1^ and 900–1100 cm^−1^) can be distinguished in the *in situ* Raman spectra of the DES and the aqueous-derived catalyst and are depicted separately in Fig. 4 and S18, respectively.

**Fig. 4:**
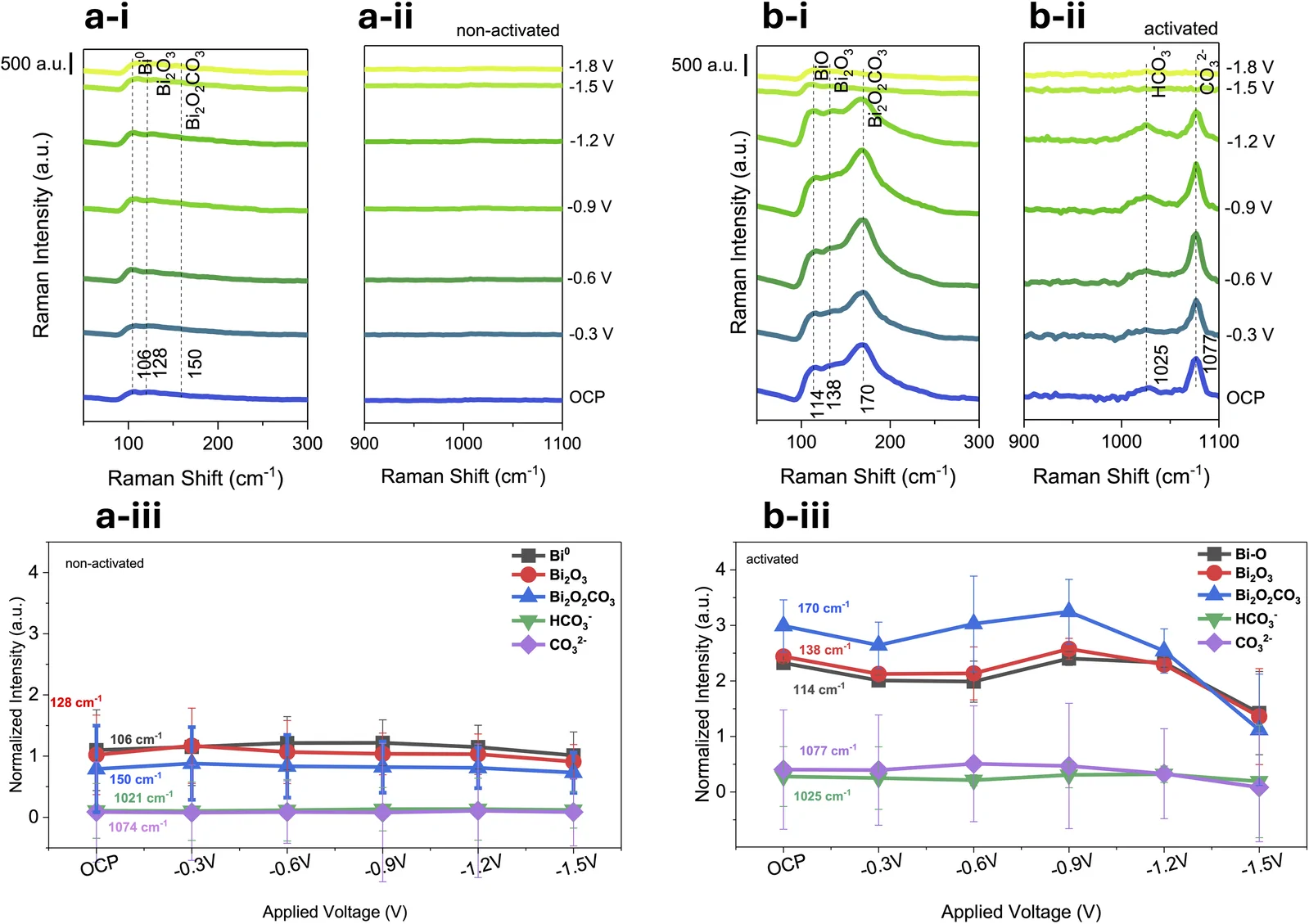
*In situ* Raman spectra of the DES-derived catalyst before (a) and after (b) electrochemical activation. (a-i and b-i) Low-wavenumber region (50–300 cm^−1^) showing the potential-dependent evolution of Bi^0^, Bi–O, Bi_2_O_3_, and Bi_2_O_2_CO_3_ bands. (a-ii and b-ii) Carbonate region (900–1100 cm^−1^) showing the HCO_3_^−^ and CO_3_^2−^ bands. (a-iii and b-iii) Corresponding normalized band intensities as a function of applied potential, non-activated (a-iii) and activated (b-iii). The equivalent data for the aqueous-derived catalyst are shown in Fig. S18.

Corresponding band assignments, literature references, and their potential-dependent evolution are summarized in Table S1.^15,50–53^ The low-frequency region contains the Bi–Bi mode of metallic Bi^0^ at 70–100 cm^−1^ and Bi–O-related modes at 111–114, 133–138, and 167–170 cm^−1^, that can be assigned to surface Bi–O species, Bi_2_O_3_, and Bi_2_O_2_CO_3_, respectively, reflecting catalyst structural evolution during polarization. The 1000–1100 cm^−1^ region contains the symmetric stretching vibrations of interfacial HCO_3_^−^ and CO_3_^2−^ species at 1021–1025 and 1074–1077 cm^−1^, respectively, which report the potential dependent carbonate equilibrium at the electrode interface.

Both catalysts exhibit the same restructuring sequence upon exposure to cathodic potential, directly evidenced by quantitative Raman evolution in the low-frequency region (Fig. 4 and S18). A pronounced difference is observed between the pristine and electrochemically activated electrodes. While the pristine catalysts exhibit only weak Bi_2_O_2_CO_3_-related features with comparatively little potential-dependent evolution, activation produces substantially stronger and dynamically evolving Bi_2_O_2_CO_3_ and Bi-oxide signatures. This demonstrates that electrochemical activation establishes a Bi surface that undergoes clear potential-dependent restructuring under reductive conditions.

The activated DES-derived catalyst shows a marked increase in the Bi_2_O_2_CO_3_ band at approximately 170 cm^−1^, reaching a maximum normalized intensity of approximately 3.2 at −0.6 V before decreasing sharply at potentials beyond −1.2 V, until they completely diminish under the most negative potentials, indicating progressive reduction toward metallic Bi. For the activated aqueous-derived catalyst, the corresponding subcarbonate band at approximately 167 cm^−1^ decreases from 2.5 at OCP to approximately 1.2 at −0.9 V and remains weak at more negative potentials, while the metallic Bi modes emerge. Thus, both catalysts undergo an oxycarbonate-to-metal transition upon cathodic polarization, although the Bi_2_O_2_CO_3_ phase is substantially more pronounced and persists to more negative potentials for the DES-derived electrode.

The transient formation of Bi_2_O_2_CO_3_ intermediates has been reported as a feature of Bi-based CO_2_RR catalysts.^15^ Incorporation of CO_2_/carbonate species into oxidic Bi produces a subcarbonate phase that remains stable in the low-overpotential regime and is progressively reduced as the potential becomes more cathodic. A directly analogous Bi_2_O_3_ → Bi_2_O_2_CO_3_ → Bi^0^ phase progression has recently been reported for basic bismuth nitrate pre-catalysts under sustained CO_2_RR operation.^54^

Comparison of Raman measurements with the corresponding LSV measurements (Fig. S20b) shows that the increase in cathodic current coincides with attenuation of the Bi_2_O_3_/Bi_2_O_2_CO_3_ bands and emergence of the metallic Bi^0^ modes. This potential-dependent correspondence identifies the oxide and oxycarbonate structures primarily as intermediate phases, and metallic Bi as the dominant working phase under strongly cathodic CO_2_RR conditions.

Control experiments were conducted on carbon paper under identical electrochemical conditions as for the target catalysts (Fig. S20a). Only the characteristic carbon D and G bands were observed, with no features in the carbonate region, consistent with the absence of Bi-related or carbonate signals in the electrolyte and carbon-paper background spectra above (Fig. S17), confirming that the Raman signals assigned to catalyst restructuring and intermediates are not influenced by the substrate.

*Ex situ* Raman spectra acquired before and after electrochemical treatment (Fig. S21) further support the transformation of the initially oxidic Bi surface toward a Bi_2_O_2_CO_3_/Bi^0^ state, consistent with the *in situ* observations. The interfacial carbonate environment also evolves distinctly for the two catalysts. The DES-derived catalyst exhibits substantially stronger CO_3_^2−^ features, with the CO_3_^2−^/HCO_3_^−^ intensity ratio (1077/1025 cm^−1^) initially increasing together with Bi_2_O_2_CO_3_ formation and subsequently decreasing at more cathodic potentials as CO_2_RR current increases and the oxycarbonate phase is reduced. This correlated evolution suggests that carbonate species participate in the dynamic restructuring of the DES-derived Bi interface during the onset of the CO_2_RR.

We additionally examined the 1300–1600 cm^−1^ region, where bands near 1460–1530 cm^−1^ have previously been attributed to adsorbed ^*^OCHO species.^15,38,55^ To evaluate the specificity of this assignment, control spectra were acquired on (i) bare carbon paper in CO_2_-saturated KHCO_3_, (ii) the activated aqueous-derived catalyst in CO_2_-saturated KHCO_3_, (iii) the activated DES-derived catalyst in N_2_-purged KHCO_3_, and (iv) the non-activated aqueous-derived catalyst in CO_2_-saturated phosphate electrolyte, to eliminate CO_3_^2−^ related features (Fig. S19). Features between 1458 and 1536 cm^−1^ were observed across these conditions, including on bare carbon paper and in the absence of CO_2_, demonstrating that they are not specific to the CO_2_RR. This spectral region overlaps with the broad D/G envelope of the carbon-paper support and may contain contributions from disorder-related carbon modes near 1500 cm^−1^. Therefore, we do not assign these features to ^*^OCHO and exclude the 1400–1600 cm^−1^ region from further mechanistic interpretation.

Overall, the *in situ* Raman data demonstrate that electrochemical activation is required to establish the dynamically restructured Bi interface, associated with enhanced CO_2_RR activity. Activation is required for both catalysts, to exhibit distinct potential-dependent Bi_2_O_2_CO_3_ formation. The subcarbonate formation is followed by its progressive reduction towards predominantly metallic Bi at more cathodic potentials. The DES-derived catalyst shows Bi_2_O_2_CO_3_ signals with higher intensity, which persist over a wider potential range and are accompanied by more pronounced changes in the interfacial carbonate environment. The enhanced formation and persistence of this subcarbonate phase correlates with the higher Bi mass-specific activity of the DES-derived catalyst, suggesting that its distinct reconstructed interface contributes to the more efficient utilization of Bi. At strongly cathodic potentials both catalysts form predominantly metallic Bi, suggesting that Bi_2_O_2_CO_3_ is a transient reconstructed phase rather than the persistent working phase. Hence, the Raman spectroscopy results link the catalytic activation and the different electrode responses to the dynamics of Bi/oxycarbonate restructuring.

### 2.6 Long-term stability CO_2_RR tests

Having established the general catalytic activity and discussed the influence of synthesis parameters on stability, we now turn to the long-term performance of the DES-derived catalyst. The stability of the DES catalyst was systematically investigated at a current density of 100 mA cm^−2^, chosen as the optimal operating condition due to its high initial formate selectivity (>90%) and relevance to industrial CO_2_ electroreduction. In a flow-cell configuration with 200 mL of 0.5 M KHCO_3_ as electrolyte in both compartments and a continuous CO_2_ flow of 10 sccm, the system initially delivered a high formate faradaic efficiency of 93% during the first hour of operation. However, prolonged electrolysis under continuous conditions led to a pronounced and steady decline in selectivity, with the formate faradaic efficiency dropping to 10% after 16 h (Fig. 5a and b, left).

**Fig. 5:**
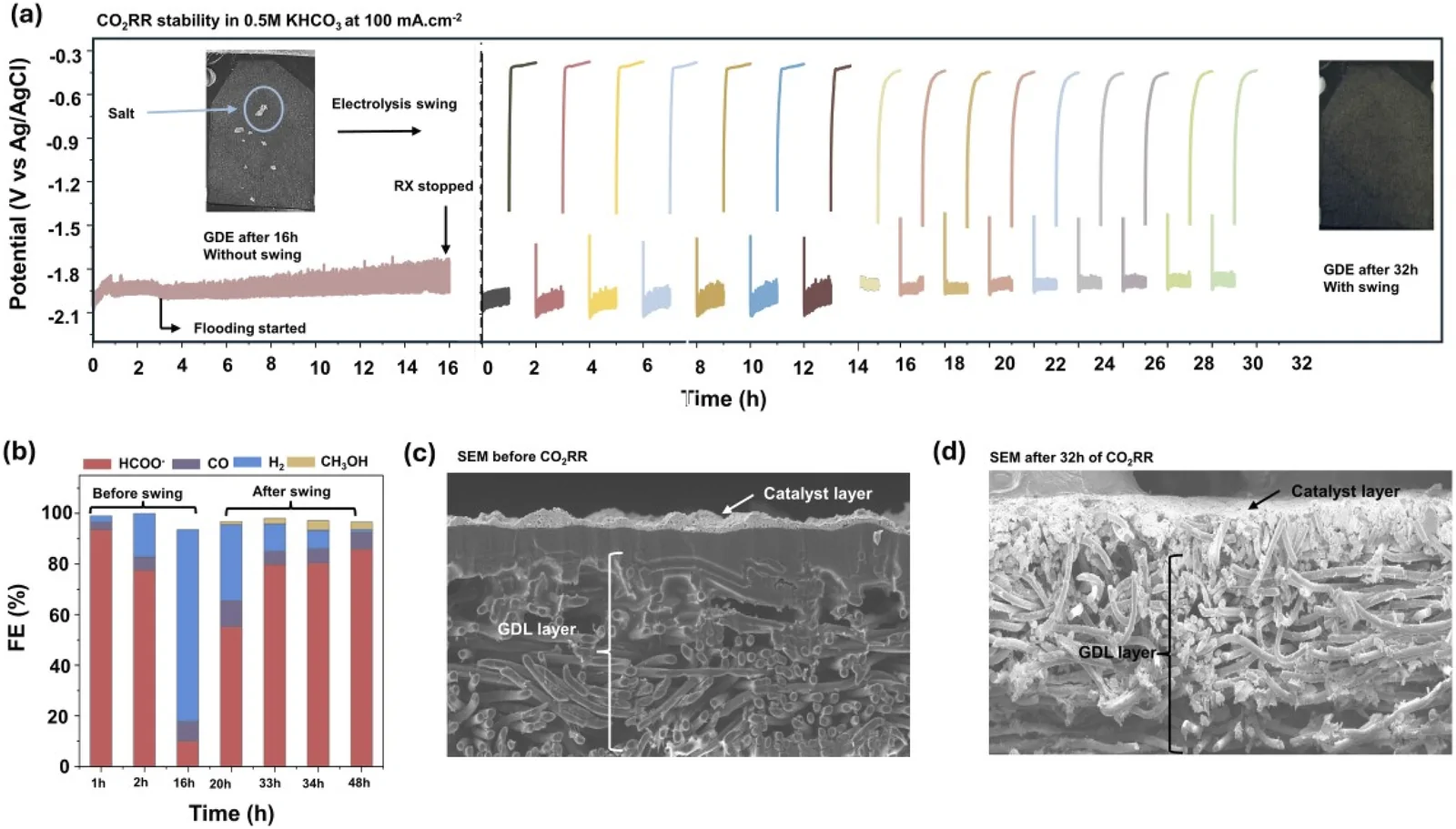
Electrochemical stability of the DES-derived catalyst during CO_2_ reduction enabled by electrolysis swing. (a) Chronopotentiometric stability test of the CO_2_ reduction reaction performed under continuous CO_2_ flow (10 sccm) in 0.5 M KHCO_3_ at a current density of 100 mA cm^−2^. Under constant electrolysis without swing, the reaction gradually deactivates due to salt accumulation on the GDE, leading to cell failure after ∼16 h (left). Implementing periodic electrolysis swing restores the electrode performance and enables stable operation for more than 32 h (right). Insets show the GDE surface after 16 h without swing, revealing salt deposition, and after 32 h with swing, where the surface remains clean. (b) Time-dependent faradaic efficiency (FE) for major products during long-term CO_2_RR operation, including formate (HCOO^−^), CO, H_2_, and CH_3_OH, showing stable product distribution throughout the extended electrolysis period. (c) Cross-sectional SEM image of the DES catalyst layer on the gas diffusion layer (GDL) before the CO_2_RR, showing a uniform catalyst coating and intact porous structure. (d) Cross-sectional SEM image of the catalyst after 32 h of the CO_2_RR under electrolysis swing conditions, demonstrating preservation of the catalyst layer and GDL structure with no significant structural degradation. All experiments were performed in a flow cell with an Ag/AgCl reference electrode, Nafion 115 membrane, and Ni foam counter electrode, using a GDE (1 × 1 cm^2^) as the working electrode.

For longer-term tests, it is important to distinguish between the degradation of the Bi catalyst and the carbon-based GDE support, as different strategies for deactivation mitigation have to be applied. In this case, the deterioration is associated with progressive flooding of the GDE, which disrupts the balance between gas and electrolyte transport within the porous structure. Post-reaction analysis confirmed extensive salt deposition and electrolyte accumulation within the GDE (Fig. S24), indicating that carbonate precipitation and cation buildup at elevated local pH promote pore blockage and loss of hydrophobicity. These effects collectively impede CO_2_ diffusion to the catalyst layer and favor competing reactions, ultimately leading to rapid performance decay and cell failure.

To overcome this limitation, a dynamic electrolysis protocol (electrolysis swing) was introduced, in which electrolysis is periodically interrupted to enable redistribution of ions and partial dissolution of accumulated salts, thereby restoring gas transport pathways.^56,57^ After the initial 16 h of continuous electrolysis, the same directly electrodeposited DES-Bi/GDE was removed from the cell, rinsed with deionized water, dried, and subsequently reused for an intermittent electrolysis protocol consisting of 1 h electrolysis followed by 1 h at open circuit potential. This second stage comprised 32 h of elapsed time, corresponding to 16 h of cumulative electrolysis (current-on time). Thus, the complete experiment consisted of 48 h of total elapsed operation and 32 h of cumulative electrolysis. As shown in Fig. 5a (right), the combination of electrode regeneration and intermittent electrolysis enabled stable operation throughout the second stage while maintaining a formate faradaic efficiency of approximately 85% (Fig. 5b). The post-electrolysis characterization shown in Fig. 5a (left side) was performed using a separate DES-Bi/GDE subjected independently to the same 16 h continuous electrolysis conditions, thereby preserving the electrode used for the extended stability experiment. Cross-sectional SEM images further corroborate these findings: the pristine GDE with a catalyst before the CO_2_RR (Fig. 5c) exhibits a uniform catalyst coating and well preserved porous structure, whereas after prolonged electrolysis under swing conditions (Fig. 5d), the catalyst layer and GDE architecture remain largely intact, with only minor perturbations in the carbon fiber network. These results demonstrate that periodic operation effectively mitigates salt-induced flooding and stabilizes electrode performance. Importantly, this strategy is readily transferable to membrane electrode assembly (MEA) systems, offering a practical pathway toward durable CO_2_ electrolysis under industrially relevant conditions. The 48 h operational limit demonstrated here is governed by GDE flooding rather than catalyst deactivation, as confirmed by the preserved Bi nanoflower morphology after prolonged operation (Fig. 5d); further lifetime extension will therefore require advances in GDE hydrophobicity retention or electrolyte management rather than catalyst redesign.

## 3 Conclusion

This work demonstrates that direct chronopotentiometric electrodeposition of bismuth from a choline chloride:ethylene glycol DES provides a facile, binder-free route to high-performance Bi/GDE catalysts at a mass loading of only ∼0.58 mg cm^−2^. Electrochemical activation results in the formation of a hierarchical nanoflower active phase. The same phase upon activation is also formed for aqueous deposited catalysts. Although the DES derived material has a 6 times lower Bi mass loading, compared to the aqueous samples, both materials show equivalent charge transfer resistance.

Raman spectroscopy illustrates that both activated catalysts undergo a common Bi_2_O_3_ → Bi_2_O_2_CO_3_ → Bi^0^ reaction pathway under CO_2_RR conditions. However, the DES derived material shows more pronounced signals of Bi_2_O_2_CO_3_ with increased stability at more cathodic potentials, indicating distinct restructuring dynamics and carbonate effects. Both catalysts deliver high formate faradaic efficiencies at current densities up to 100 mA cm^−2^ (96.8% and 93.7% for the DES- and aqueous-derived catalysts, respectively), at which point the DES-derived catalyst achieves a mass-normalised HCOOH partial current density of 166.9 mA mg_Bi_^−1^, approximately 6-fold higher than that of the aqueous-derived catalyst (26.7 mA mg_Bi_^−1^). The activated state, not the as-deposited morphology, governs catalytic selectivity. At higher current densities, the larger Bi reservoir of the aqueous electrode provides greater tolerance to dissolution-induced active-site loss, retaining an FE_HCOOH_ of 88.9% at 150 mA cm^−2^ and 77.0% at 200 mA cm^−2^, compared to 50.9% and 21.5% for the DES-derived catalyst. DES electrodeposition therefore provides its principal advantage in the 30–100 mA cm^−2^ operating range, combining substantially lower Bi consumption with high mass-specific activity. Importantly, this material efficiency is retained upon scale-up. Electrodeposition on 150 cm^2^ substrates yields 95.5% formate faradaic efficiency for the DES-derived electrode compared with 70.2% for aqueous deposition, demonstrating the benefit of the more uniform DES-derived catalyst distribution at technologically relevant electrode dimensions. Finally, progressive GDE flooding *via* carbonate salt precipitation was identified as the major degradation mechanism under sustained operation. A periodic electrolysis swing protocol (1 h on/1 h rest) mitigated this effect, extending operation at 100 mA cm^−2^ to 48 h of total elapsed time, corresponding to 32 h of cumulative electrolysis, while maintaining 85% formate selectivity. This demonstration of electrolyte-mediated morphology control *via* DES electrodeposition broadens the scope of solvent engineering beyond conventional aqueous synthesis and decouples high catalytic performance from high catalyst loading. This strategy can enable development of new electrodeposition strategies for the scalable fabrication of binder-free GDE catalysts for CO_2_ electrolysis and beyond.

## 4 Experimental section

### 4.1 DES and electrolyte preparation

To prepare 100 mL of DES, choline chloride (ChCl) and ethylene glycol (EG) were mixed at a molar ratio of 1 : 2. First, 58 g of ChCl was placed in a beaker containing a magnetic stirrer, followed by the addition of 46 mL of EG. The mixture was stirred at 300 rpm while being heated to 90 °C for 2 h, until a homogeneous transparent solution was obtained.

To prepare a 0.1 M BiCl_3_ solution in DES, 3.153 g of bismuth chloride (BiCl_3_, molar mass 315.34 g mol^−1^) was added to the prepared DES. The solution was stirred at 300 rpm and heated to 50 °C for 6 h until the BiCl_3_ was fully homogenized, resulting in a milky solution. For the aqueous electrolyte, 3.153 g of BiCl_3_ was dissolved in 100 mL of 10% v/v HCl solution in deionized water with stirring at 300 rpm at room temperature until complete dissolution, yielding a transparent BiCl_3_ solution.

### 4.2 GDE preparation

The electrolyte was transferred into a 100 mL beaker and stirred briefly. For DES electrodeposition, the electrolyte was maintained at 50 °C with stirring at 150–300 rpm. A counter electrode (Pt foil) was secured inside the beaker using a lid, and the working electrode (WE) was immersed in the electrolyte, ensuring that the counter electrode and WE faced each other. Electrodeposition was carried out using chronopotentiometry at a current density of 4 mA cm^−2^ for 500 s. The deposition current density was varied across 2, 4, 18, and 36 mA cm^−2^ for additional samples. After deposition, the carbon paper was immediately removed and rinsed with deionized (DI) water to remove excess DES. The electrodeposited sample was then dried in a vacuum oven at 50 °C for 1 h and stored in a vacuum desiccator. Aqueous electrodeposition followed the same procedure, with the exception that the electrolyte was maintained at room temperature throughout. The electrodeposited samples are labelled as DES-2, DES-4, DES-18, and DES-36 for deposition current densities of 2, 4, 18, and 36 mA cm^−2^, respectively, with analogous labels Aq-2, Aq-4, Aq-18, and Aq-36 for the aqueous-derived counterparts.

### 4.3 Electrochemical experiments in the flow cell at TUW

All electrochemical experiments were conducted within a microflow cell purchased from Electro-cell, as previously reported,^9^ with electrode areas of 2 × 1 cm^2^. The cell comprises three compartments: one for the anolyte, one for the catholyte, and one for the gas. All experiments were conducted at room temperature and pressure using an Autolab PGSTAT302N potentiostat in a three-electrode flow cell setup. An IrO_*x*_-coated Ti current collector plate served as the anode, while a leakless Ag/AgCl micro reference electrode was used as the reference electrode. Freshly prepared 0.5 M KHCO_3_ and 0.5 M KOH solutions were used as the catholyte and anolyte, respectively, and were circulated at a flow rate of 45 mL min^−1^ using a peristaltic pump. CO_2_ was introduced at a flow rate of 30 sccm. The catalyst GDE, anion exchange membrane (AEM), and anode plate were assembled within the flow cell, and the electrolytes and gas were circulated for 30 min to saturate the system.

Electrochemical activation was achieved through 16 CV scans ranging from 0 to −2 V *vs.* Ag/AgCl. All CV and linear sweep voltammetry (LSV) experiments were performed at a scan rate of 100 mV s^−1^, with potentials corrected for 85% of the internal resistance (IR). Chronopotentiometry was employed to assess CO_2_RR performance at various current densities over 4500 s.

The ohmic drop was quantified through EIS, where the real component of the Nyquist plot at 1000 Hz provided the solution resistance (*R*_s_). Manual adjustment for 85% IR compensation was carried out during data analysis. EIS measurements were conducted at different potentials *vs.* Ag/AgCl. The resulting Nyquist plots were fitted using Zview software with an equivalent circuit model comprising *R*_s_ − *R*_CT_/CPE, where *R*_s_ represents the solution resistance, *R*_CT_ denotes the charge transfer resistance, and CPE signifies the constant phase element.

### 4.4 Product analysis at TUW

#### 4.4.1 Gaseous products

Gaseous product collection was initiated after 400 s of electrolysis. Products were collected every 30 min from the outlet of the flow cell into Tedlar gas sampling bags and injected into a Shimadzu Nexis GC-2030 gas chromatograph using a gas-tight syringe. Helium was used as the carrier gas at a total flow rate of 35.4 mL min^−1^ in pressure flow control mode. The inlet pressure was initially set at 226.8 kPa and held for 2.5 min, then increased at 15.2 kPa min^−1^ to 390.1 kPa and held for 5.95 min, and finally increased at 11.2 kPa min^*−*1^ to 405.1 kPa and held for 5.42 min. A ShinCarbon ST micro-packed GC column (100/120, 1 mm ID, 2 m) was used. The column temperature program started at 50 °C, held for 2.5 min, then increased at 10 °C min^−1^ to 250 °C, followed by a further increase at 9 °C min^−1^ to 270 °C, held for 8 min. A Barrier Ionization Discharge (BID) detector was used at a detector temperature of 280 °C with a He discharge flow rate of 50 mL min^−1^.

#### 4.4.2 Liquid products

Cumulative liquid products were analyzed post-electrolysis using a Shimadzu LCMS-2020 liquid chromatograph-mass spectrometer with a Shim-pack SCR-102H ion exchange column (300 × 8.0 mm, 7 µm) maintained at 70 °C. Detection was performed using a UV SPD-40V detector at a wavelength of 220 nm. The mobile phase flow rate was maintained at 0.8 mL min^−1^.

The faradaic efficiency for gaseous products is given by using eqn (1):1
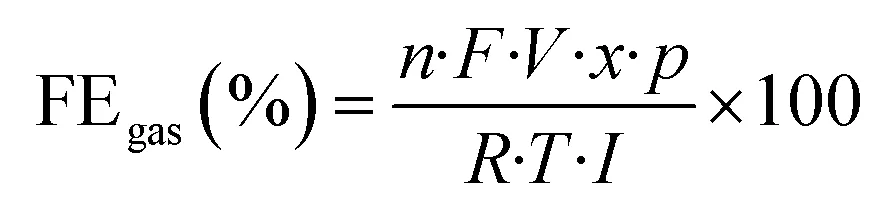


For liquid products, the faradaic efficiency is calculated using eqn (2):2
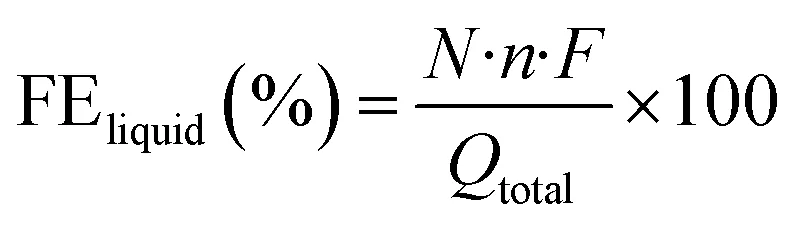
where *n* is the number of electrons transferred per molecule, *F* is the Faraday constant (96 485C mol^−1^), *V* is the volumetric flow rate, *x* is the mole fraction of the gaseous product, *p* is the pressure, *R* is the ideal gas constant (8.314 J mol^−1^ K^−1^), *T* is the temperature, *I* is the average current, *N* is the amount of substance of the product of interest, and *Q*_total_ is the total charge passed.

### 4.5 Characterization at TUW

Electron microscopy was conducted using a Zeiss Sigma EDVP scanning electron microscope (SEM) equipped with an Ametek EDAX analyzer for energy-dispersive X-ray spectroscopy (EDX). X-ray photoelectron spectroscopy (XPS) measurements were performed using a VersaProbe III spectrometer (Physical Electronics GmbH) at the Electrochemical Surface and Interface Analysis (ELSA) facility, TU Vienna. Monochromated Al Kα radiation (1486.6 eV) was used with a beam diameter of 200 µm and a beam voltage of 15 kV. Samples were mounted on double-sided copper tape; charge neutralization was applied during measurements to compensate for surface charging effects. Prior to analysis, sample surfaces were cleaned using a gas cluster ion beam (GCIB) sputter gun. Survey spectra were recorded at a pass energy of 140 eV and a step size of 0.125 eV. High-resolution core level spectra were recorded at a pass energy of 27 eV and a step size of 0.05 eV. The binding-energy scale was referenced to the Fermi edge of Bi. Spectra were processed in CasaXPS (Version 2.3.26PR1.0) using a Shirley background. Components were fitted with LA(50) line shapes, except for metallic Bi^0^, which was fitted with an asymmetric TLA(10,90) line shape. The Bi 4f spin–orbit splitting was fixed at 5.31 eV and the Bi^0^ 4f_7/2_ position was constrained to 156.8–157.0 eV; splittings of 1.6 eV (Cl 2p) and 2.8 eV (K 2p) were used. Atomic concentrations were calculated from the fitted peak areas corrected for relative sensitivity factors, analyser transmission, and inelastic mean free path. For the chloride-removal control, pristine electrodes were assembled in the flow cell and DI water was circulated for 3 h without applied potential prior to XPS analysis.

#### 4.5.1 Raman spectroscopy

Raman spectroscopy measurements were performed using a LabRAM Aramis Raman microscope (Horiba Jobin Yvon, Germany) equipped with a 532 nm laser excitation source (50 mW power).^58^ Calibration was performed using a silicon wafer standard (520.6 cm^−1^). Spectra were collected using a 50× objective (4.0 mm focal length; numerical aperture 0.55) with a 200 µm entrance slit, a 500 µm confocal hole, and a 600/mm grating, covering a Raman shift range of approximately 50–2000 cm^−1^. Signals were collected using LabSpec 5.19 software.

For *ex situ* measurements, samples were analyzed under ambient conditions with an acquisition time of 50 s and 3 accumulations to improve the signal-to-noise ratio.

*In situ* Raman measurements were carried out during electrochemical CO_2_ reduction by applying a constant potential to the working electrode in the Raman cell (DekResearch), as shown in Fig. S16. CO_2_-saturated 0.5 M KHCO_3_ was used as the electrolyte. After applying each potential using a PalmSens4 potentiostat, the system was allowed to equilibrate for 30 s, followed by an additional 100 s stabilization period to allow the current to reach a steady state before spectral acquisition at a scan rate of 0.1 V s^−1^ using PSTrace 5.11 software. Raman spectra were then collected with an acquisition time of 50 s and 10 accumulations while maintaining the applied potential, resulting in a total chronoamperometric hold time of 600 s per potential step. Measurements were performed sequentially over a potential range from −0.3 to −1.8 V *vs.* Ag/AgCl. Raw *in situ* spectra were baseline-corrected using asymmetric least squares smoothing in OriginPro (asymmetry factor 1 × 10^−5^, threshold 5 × 10^−5^, smoothing factor 4, 4 iterations). Baseline-corrected spectra were intensity-normalized to the Bi_2_O_3_ peak at 480–490 cm^−1^, dividing all intensities by 330 for aqueous-derived samples and by 300 for DES-derived samples.

X-ray diffraction (XRD) patterns were recorded using a Malvern PANalytical Empyrean diffractometer. Metal-deposited GDEs were directly placed on the XRD sample holder, and data were collected over a 2*θ* range of 4–90°.

### 4.6 Electrochemical measurements at DTU

#### 4.6.1 Nafion membrane pre-treatment

The Nafion 115 membrane was pretreated prior to use. The membrane was first boiled in a 3% hydrogen peroxide (H_2_O_2_) solution at 80 °C for 1 h, then rinsed three times with Milli-Q (MQ) water and boiled in MQ water at 80 °C for 2 h. Subsequently, the membrane was boiled in 0.5 M sulfuric acid (H_2_SO_4_) at 80 °C for 1 h, followed by three additional rinses with MQ water. The pretreated membrane was stored in 1 M KHCO_3_ until use.

#### 4.6.2 Electrochemical setup

All electrochemical measurements were performed using a BioLogic potentiostat/galvanostat controlled by EC-Lab software in a flow cell configuration under ambient conditions (Fig. S25). Ni foam served as the counter electrode, Ag/AgCl as the reference electrode, and a pretreated Nafion 115 membrane separated the two compartments. The working electrode used for the long-term stability measurements was the directly electrodeposited, binder-free DES-Bi/GDE prepared by chronopotentiometric electrodeposition as described above. The electrode had a Bi loading of 0.58 mg cm^−2^ and an active area of 1 × 1 cm^2^, and was evaluated using 0.5 M KHCO_3_ electrolyte in both compartments and a continuous CO_2_ flow of 10 sccm.

#### 4.6.3 Stability test

The longer-term CO_2_RR behavior of the directly electrodeposited DES-Bi/GDE was evaluated by chronopotentiometry at 100 mA cm^−2^ using 200 mL of 0.5 M KHCO_3_ (22 mL min^−1^) in each compartment under a continuous CO_2_ flow of 10 sccm. The electrode was initially operated continuously for 16 h, during which a progressive decrease in formate selectivity was observed. It was subsequently removed from the cell, rinsed with deionized water, dried on a hot plate for 15 min at 75 °C, and reinstalled in the flow cell. The same electrode was then operated for an additional 32 h of elapsed time using an electrolysis swing protocol consisting of 1 h of electrolysis at 100 mA cm^−2^ followed by 1 h at open circuit potential. The intermittent stage therefore comprised 16 h of cumulative current-on time. Overall, the complete experiment comprised 48 h of total elapsed time and 32 h of cumulative current-on time. Liquid samples were collected every 1–2 h for ^1^H NMR analysis. At each liquid sampling time point, the gaseous products were simultaneously analyzed using online GC.

Post-electrolysis characterization representing the electrode condition after 16 h of continuous operation was conducted using a separate DES-Bi/GDE subjected independently to the same 16 h continuous-electrolysis conditions. A separate electrode was used for this characterization because the electrode employed in the extended stability test was retained intact for the subsequent intermittent-operation stage.

### 4.7 Product analysis at DTU

#### 4.7.1 NMR spectroscopy

^1^H NMR spectra were recorded on a JEOL 400 MHz spectrometer at a proton resonance frequency of 400 MHz at 298 K. Measurements were carried out using a 5 mm broadband probe with standard pulse sequences, accumulating 16–64 scans depending on sample concentration. Chemical shifts (*δ*) are reported in parts per million (ppm) relative to the residual solvent peak, and coupling constants (*J*) are given in hertz (Hz). NMR samples were prepared by mixing 550 µL of the electrolyte sample with 40 µL of 40 mM maleic acid as an internal standard and 10 µL of D_2_O.

The molar concentration of each liquid product *C*_*x*_ was determined from the ^1^H NMR spectra using eqn (3):3
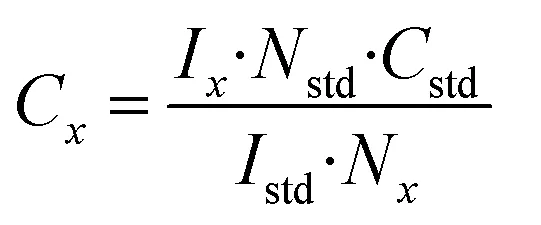
where *I*_*x*_ and *I*_std_ are the integrals of the product and maleic acid standard peaks, respectively, *N*_std_ and *N*_*x*_ are the number of equivalent protons in maleic acid and the product, respectively, and *C*_std_ is the molar concentration of maleic acid in the NMR tube (40 mM).

The faradaic efficiency for each liquid product was then calculated using eqn (4):4
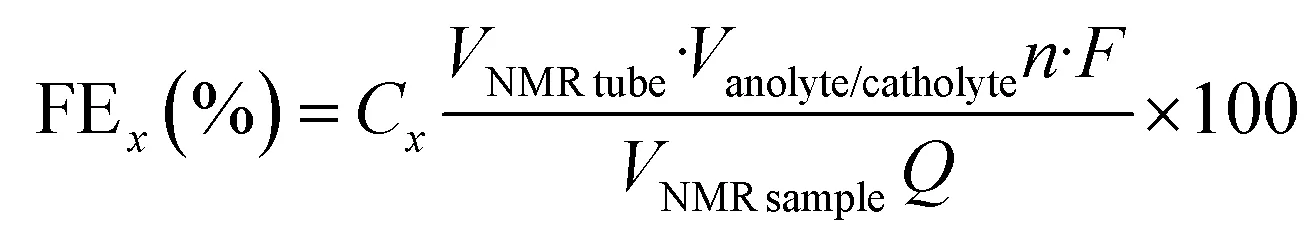
where *V*_NMR tube_ = 600 µL is the total liquid volume in the NMR tube, *V*_NMR sample_ = 550 µL is the volume of the electrolyte sample, *V*_anolyte/catholyte_ is the total electrolyte volume, *n* is the number of electrons transferred per molecule, *F* is the Faraday constant (96 485C mol^−1^), and *Q* is the total charge passed through the working electrode.

#### 4.7.2 Gas chromatography

GC analyses were carried out using a Thermo Scientific TRACE 1310 GC system equipped with a suitable capillary column under controlled carrier gas flow and temperature-programmed conditions. The faradaic efficiency for gaseous products was calculated using eqn (5):5
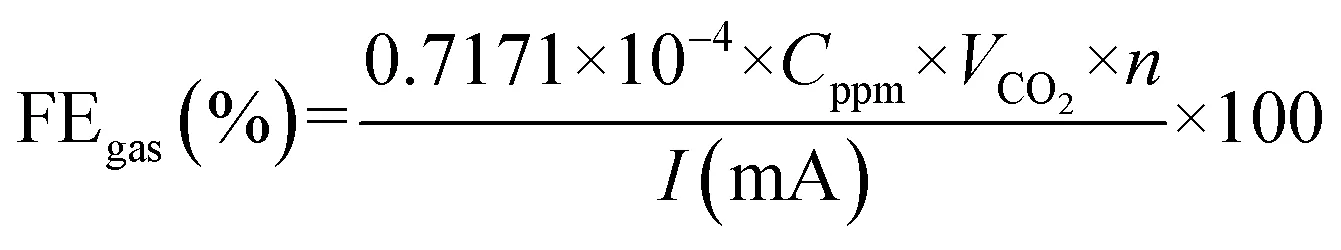
where *C*_ppm_ is the product concentration in ppm, *V*_CO_2__ is the CO_2_ flow rate (mL min^−1^), *n* is the number of electrons transferred per molecule, and *I* is the average current in mA.

## Conflicts of interest

There are no conflicts to declare.

## Supplementary Material

TA-OLF-D6TA04799H-s001

## Data Availability

Data for this article, including Raman and XPS spectra, electrochemical experiments and material analysis, are available at reposiTUm at https://researchdata.tuwien.ac.at. Supplementary information (SI): additinoal experimental descriptions, electron microscopic images, XRD, XPS and further electrochemical analysis. See DOI: https://doi.org/10.1039/d6ta04799h.
