# Supplementary material for: Electrodeposited bismuth nanomaterials as binder free and scalable catalysts for highly selective CO2 to formate conversion
Source: J Mater Chem A Mater. 2026 Oct 12. Online ahead of print. doi: 10.1039/d6ta04799h (PMC13720869; doi:10.1039/d6ta04799h)
Supplement: Supplementary file 1 [file TA-OLF-D6TA04799H-s001.pdf]

Supplementary Information (SI) for Journal of Materials Chemistry A.
This journal is © The Royal Society of Chemistry

Supporting Information

Electrodeposited Bismuth Nanomaterials as Binder Free and Scalable Catalysts for Highly Selective CO_2 to Formate Conversion

Anil Kumar Sihag,[a] Yashraj Kumar Singh,[a,b] Esraa Kotob,[c] Sarah Zulfa Khairunnisa,[a] Markus Ostermann,[a] Olga Guselnikova,[a] Markus Valtiner,[a] Maryam Abdinejad,[c] Christian M. Pichler[a,*]

[a]Institute of Applied Physics, Vienna University of Technology, 1040 Vienna, Austria
[b]Indian Institute of Science Education and Research, Pune 411008, India
[c]Department of Energy Conversion and Storage, Technical University of Denmark, 2800 Lyngby, Denmark

*Correspondence: pichlerc@iap.tuwien.ac.at

S1 Instruments and Experimental Procedures

Chemicals and Materials at TUW

Bismuth chloride ($BiCl_3$), choline chloride (ChCl), ethylene glycol (EG), $KHCO_3$ (99.5%), and KOH (99.9%) were purchased from Sigma-Aldrich and used as received. PTFE-treated carbon papers with a microporous layer (Freudenberg H23C6) were obtained from the Fuel Cell Store and subsequently cut into the desired dimensions (2 cm × 1 cm) using a razor blade. The entire microflow cell assembly, including a leakless Ag/AgCl micro reference electrode and an IrO_2-coated titanium plate for the counter electrode, was sourced from Electrocell. Sustainion® X37-50 Grade RT anion exchange membranes (AEMs) were purchased from Dioxide Materials. AEMs were converted into the hydroxide form by immersion in 1 M KOH for more than 24 h prior to use.

Chemicals and Materials at DTU

Deuterium oxide (D_2O, >99.8%D) was purchased from Avantor (VWR). All experimental solutions were prepared using deionized water purified by a Millipore Milli-Q water purification system (18.2 MΩ cm). A Pt counter electrode, Ag/AgCl reference electrode, glassy carbon electrode, single-compartment electrochemical cell, and H-cell were obtained from BASi (West Lafayette, IN, USA). Nafion 115 membrane was supplied by Ion Power GmbH.

Characterization at DTU

Ion Milling: Cross-sectional samples for scanning electron microscopy (SEM) analysis were prepared using a Hitachi E-3500 ion mill. Ion milling was employed to obtain smooth, damage-free cross-sections of the electrode structure, enabling high-resolution imaging of the catalyst layer and gas diffusion layer interface.

Scanning Electron Microscopy: Cross-sectional morphology was characterized using a field-emission scanning electron microscope (FE-SEM, Zeiss Ultra) equipped with a high-brightness field emission electron source and multiple detectors, including in-lens secondary electron and backscattered electron detectors. Imaging was performed at accelerating voltages in the range of 0.1–30 kV, providing spatial resolution down to ~1 nm at 15 kV and magnifications up to ~900,000×.

S2 Supporting Figures

Double Layer Capacitance and Interfacial Charge-Transfer Kinetics

The double-layer capacitance and EIS charge-transfer resistance data presented here were used to select representative electrodes for CO_2RR testing and to confirm the equivalence of the activated DES and aqueous catalyst surfaces discussed in the main manuscript.

The double-layer capacitance (C_{dl}) was measured by scan-rate-dependent cyclic voltammetry in the non-faradaic potential window (-0.35 to -0.45 V vs. Ag/AgCl) in CO_2-saturated 0.5 M $KHCO_3$ as catholyte in the flow cell setup (Figures S26 and S27). The capacitive current difference ($|j_{\mathrm{a}} - j_{\mathrm{c}}|/2$) scales linearly with scan rate for all samples, and the slope of the linear fit directly yields C_{dl} (Table S2).

Within the aqueous series, C_{dl} increases from 2.8 mF at 2 mA cm^{-2} to 2.19 mF at 4 mA cm^{-2} and 3.87 mF at 18 mA cm^{-2}, reflecting the progressively denser Bi network formed at higher deposition currents. The DES series increases monotonically, with C_{dl} increasing from 0.62 mF at 2 mA cm^{-2} to 1.43 mF at 4 mA cm^{-2} and 8.04 mF at 18 mA cm^{-2}. At equivalent deposition current density, DES-derived electrodes exhibit lower C_{dl} than their aqueous-derived counterparts at 2 and 4 mA cm^{-2}, but higher capacitance at 18 mA cm^{-2}.

Although C_{dl} is frequently used as a relative descriptor of electrochemically accessible surface area, we refrain from converting these values into absolute ECSA. Such a conversion requires knowledge of the specific interfacial capacitance (C_s) and assumes that C_s is comparable among the investigated electrodes. This assumption cannot be established for the present porous Bi/GDE architecture, in which the measured capacitance may depend not only on Bi surface area but also on nanoscale surface structure, electrolyte wetting and pore accessibility, surface oxide/hydroxide species, and contributions from the underlying porous carbon electrode.

This limitation is particularly evident for the DES-derived series. The electrodes deposited at 4 and 18 mA cm^{-2} possess nearly identical Bi loadings (0.581 and 0.560 mg cm^{-2}, respectively), whereas their measured C_{dl} values differ substantially (1.43 and 8.04 mF). No correspondingly large difference in the activated electrode morphology/structure is resolved by SEM/XRD. Consequently, assigning the approximately 5.6-fold difference in C_{dl} exclusively to a difference in catalytically active Bi surface area would not be sufficiently supported by the available characterization. Therefore, we report C_{dl} as an experimental descriptor of the electrochemically accessible electrode/electrolyte interface, rather than using it to calculate absolute ECSA or surface-area-normalized catalytic activity.

The selected DES-4 electrode exhibits a lower measured capacitance than Aq-18 (1.43 versus 3.87 mF), while both deliver high formate Faradaic efficiencies under CO_2RR conditions.

The interfacial charge-transfer kinetics of the activated electrodes were further characterized by electrochemical impedance spectroscopy (EIS) at potentials of -1.3 V and -1.5 V vs. Ag/AgCl (Figure S10). Nyquist plots for all samples display a single depressed semicircle, and equivalent circuit fitting yields the solution resistance (R_s) and charge-transfer resistance (R_{ct}). The solution resistance is invariant across all samples (4.08–5.38 Ω). Within the DES series at -1.3 V (Figure S10a), R_{ct} decreases progressively from 86.36 Ω at 2 mA cm^{-2} to 42.98 Ω at 18 mA cm^{-2}, reflecting improved charge transfer as higher Bi loading generates a more percolated and electrically connected catalyst network. In contrast, the 36 mA cm^{-2} DES electrode exhibits a markedly elevated R_{ct} of 141.80 Ω, substantially higher than all lower-loading DES samples.

The most decisive comparison is revealed at -1.5 V (Figure S10b), where the aqueous series is benchmarked directly against the DES-4 mA cm^{-2} electrode. For the aqueous catalysts, R_{ct} decreases sharply with increasing deposition current, from 160.9 Ω at 2 mA cm^{-2} to 27.08 Ω at 4 mA cm^{-2} and 12.28 Ω at 18 mA cm^{-2}, consistent with the large fraction of Bi lost during activation at low aqueous loadings and the consequent need for substantial over-deposition to sustain a conducting Bi network. The DES-4 mA cm^{-2} electrode yields an R_{ct} of 13.16 Ω, statistically equivalent to the aqueous 18 mA cm^{-2} electrode (12.28 Ω), despite being deposited at 4.5-fold lower current density and carrying a 6-fold lower Bi mass loading. This result provides direct electrochemical evidence that DES electrodeposition generates a Bi catalyst network with charge-transfer characteristics equivalent to a heavily loaded aqueous electrode while requiring substantially less material, confirming that DES electrodeposition achieves markedly higher Bi utilization efficiency than aqueous deposition.

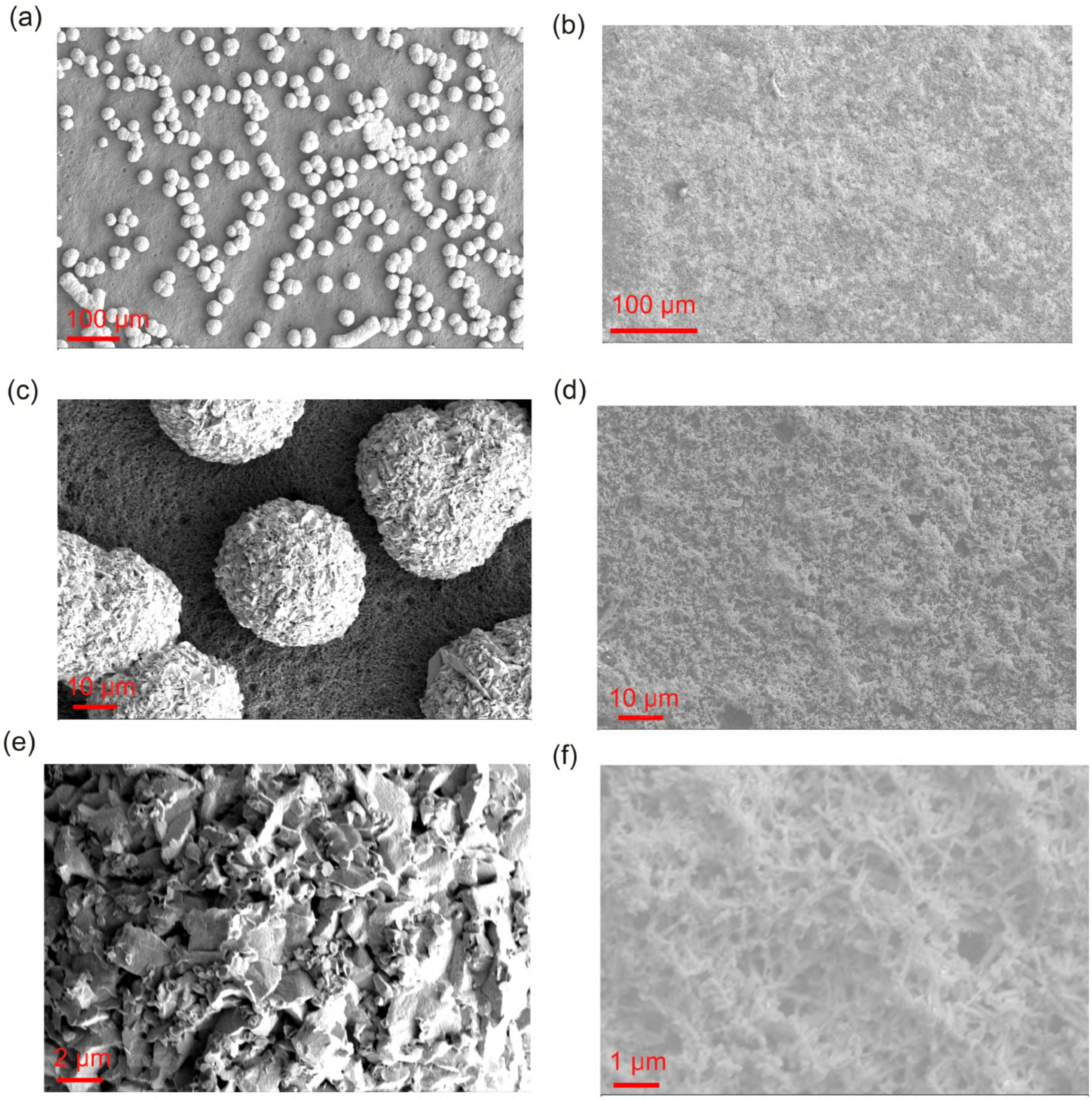

Fig. S1 SEM images of Bi catalysts at different magnifications. (a,c,e) Aqueous-derived (electrodeposited at 18 mA cm^{-2}) catalyst and (b,d,f) DES-derived (electrodeposited at 4 mA cm^{-2}) catalyst. Images include pristine electrodes as well as samples after electrochemical activation and CO_2RR operation. Scale bars are indicated in each image.

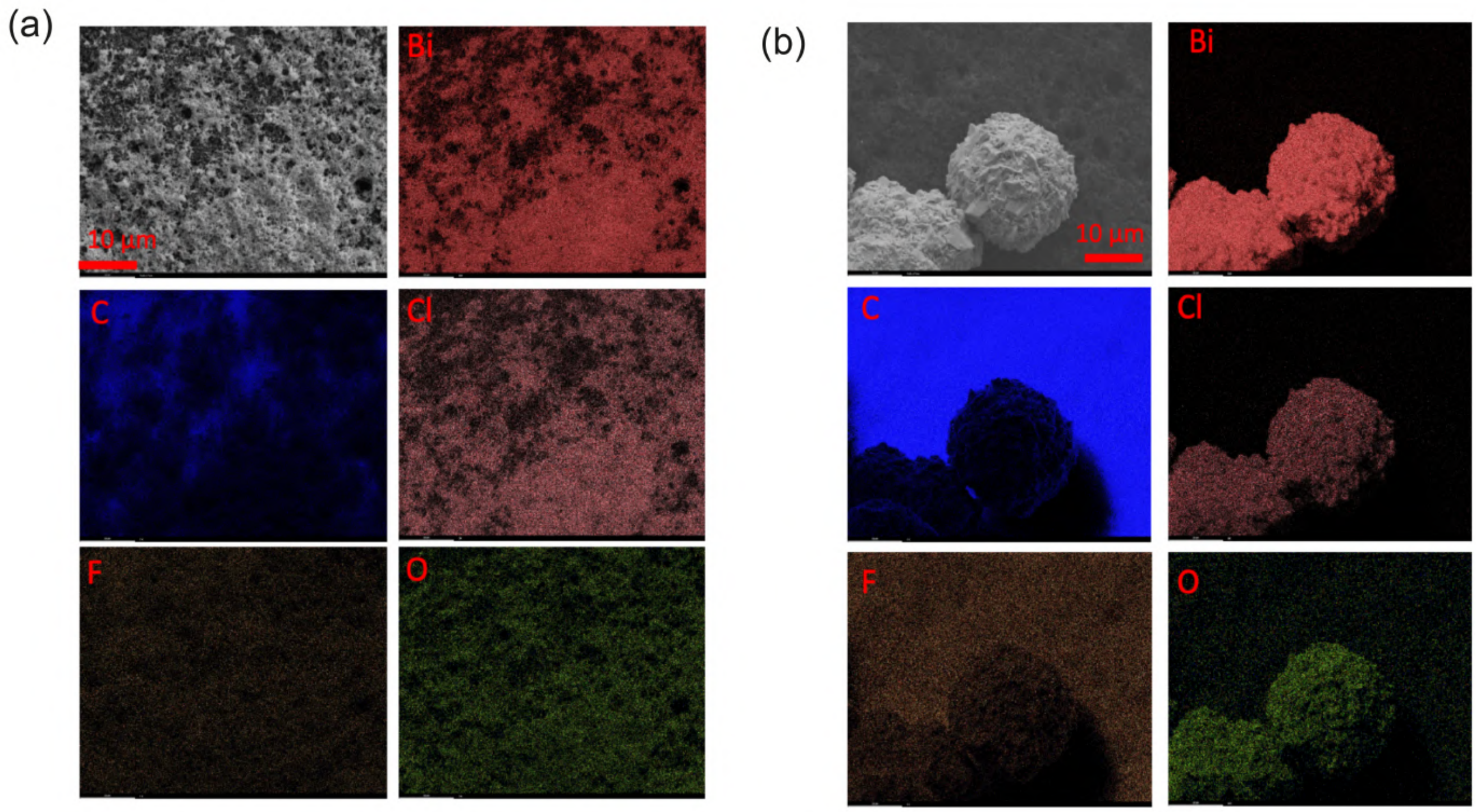

Fig. S2 SEM images and corresponding EDX elemental maps of pristine Bi catalysts electrodeposited from (a) deep eutectic solvent (DES) and (b) aqueous electrolyte. The elemental distributions of Bi, C, Cl, F, and O are shown. The Bi signal confirms the presence of deposited bismuth across the electrode surface, while C originates from the carbon paper substrate. The Cl signal is associated with residual species from the deposition electrolyte, while the F signal originates from the PTFE-based hydrophobic coating of the GDE substrate.

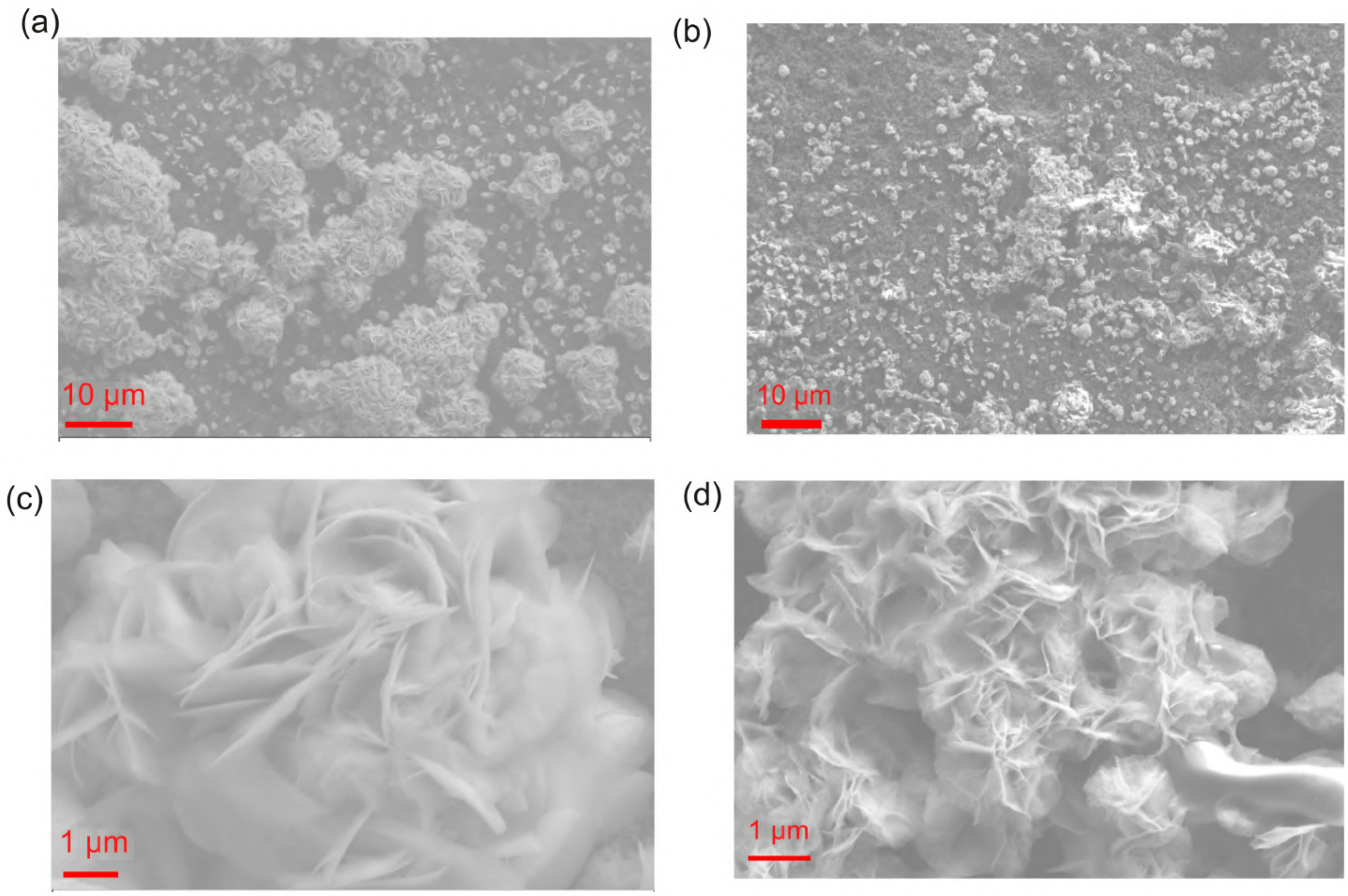

Fig. S3 SEM images of Bi catalysts after CO_2RR operation at 100 mA cm^{-2} showing the formation of nanostructured Bi morphologies. (a,c) Aqueous-derived (electrodeposited at 18 mA cm^{-2}) catalyst and (b,d) DES-derived (electrodeposited at 4 mA cm^{-2}) catalyst. Low-magnification images (a,b) illustrate the distribution of nanostructured Bi clusters on the carbon substrate, while high-magnification images (c,d) reveal the formation of flower-like assemblies composed of thin nanosheets. Scale bars: 10 μm (a,b) and 1 μm (c,d).

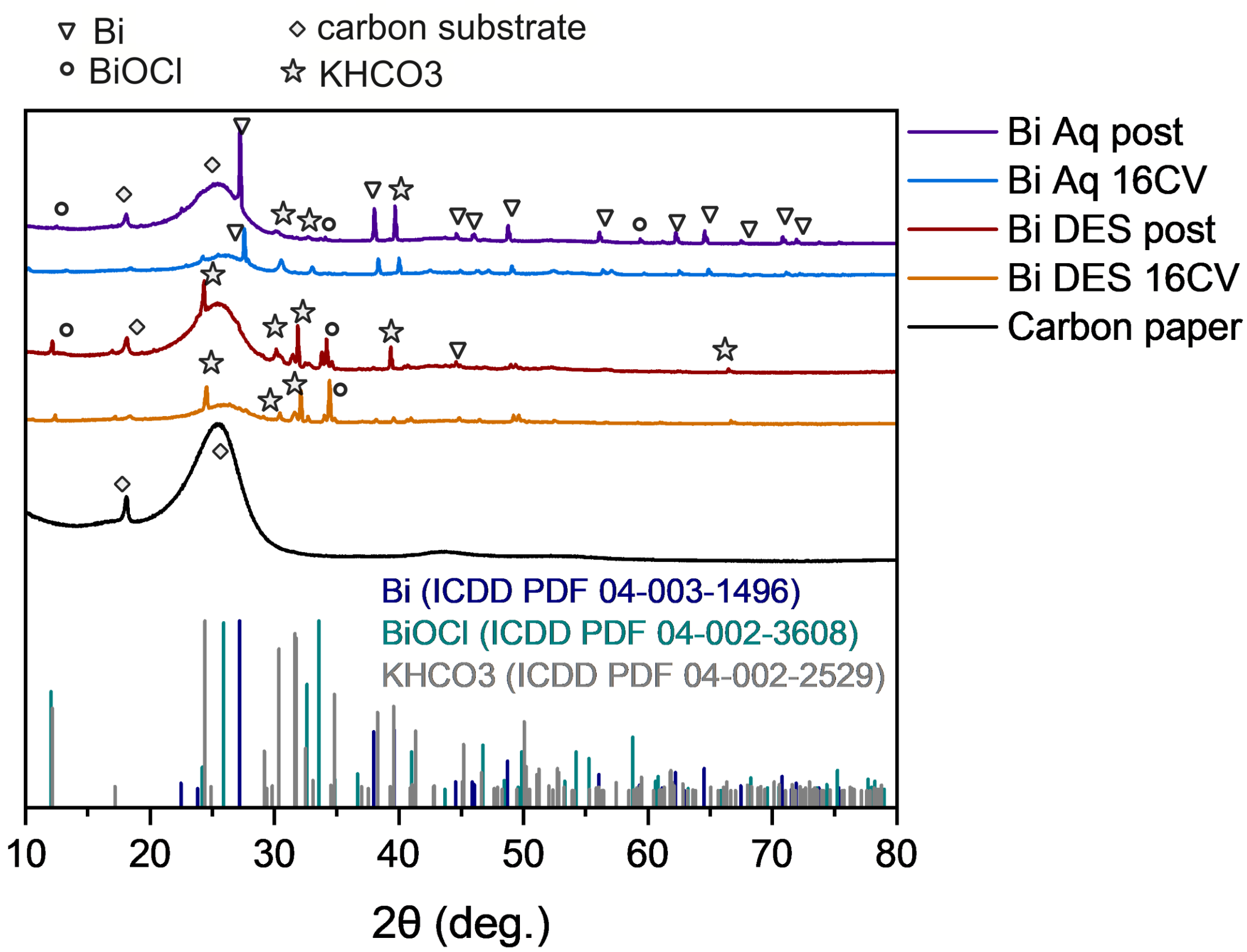

Fig. S4 XRD patterns of Bi catalysts after electrochemical activation (16 CV cycles, "16CV") and after CO_2RR operation at 100 mA cm^{-2} ("post"), for both aqueous- and DES-derived electrodes, together with the bare carbon paper substrate for reference. Symbols denote ▽ metallic Bi (ICDD PDF 04-003-1496), ∘ BiOCl (ICDD PDF 04-002-3608), ⋆ $KHCO_3$ (ICDD PDF 04-002-2529), and ◇ the carbon paper substrate, including its PTFE hydrophobic treatment. Corresponding reference stick patterns are shown at the bottom. Symbols are placed on representative traces; the same reflections occur in the remaining patterns. Patterns are vertically offset for clarity and intensities are not normalised. The $KHCO_3$ reflections arise from electrolyte residues precipitated within the porous electrode structure during electrolysis and are most pronounced in the DES-derived samples, where the low Bi loading (0.58 mg cm^{-2}) gives correspondingly weak Bi reflections.

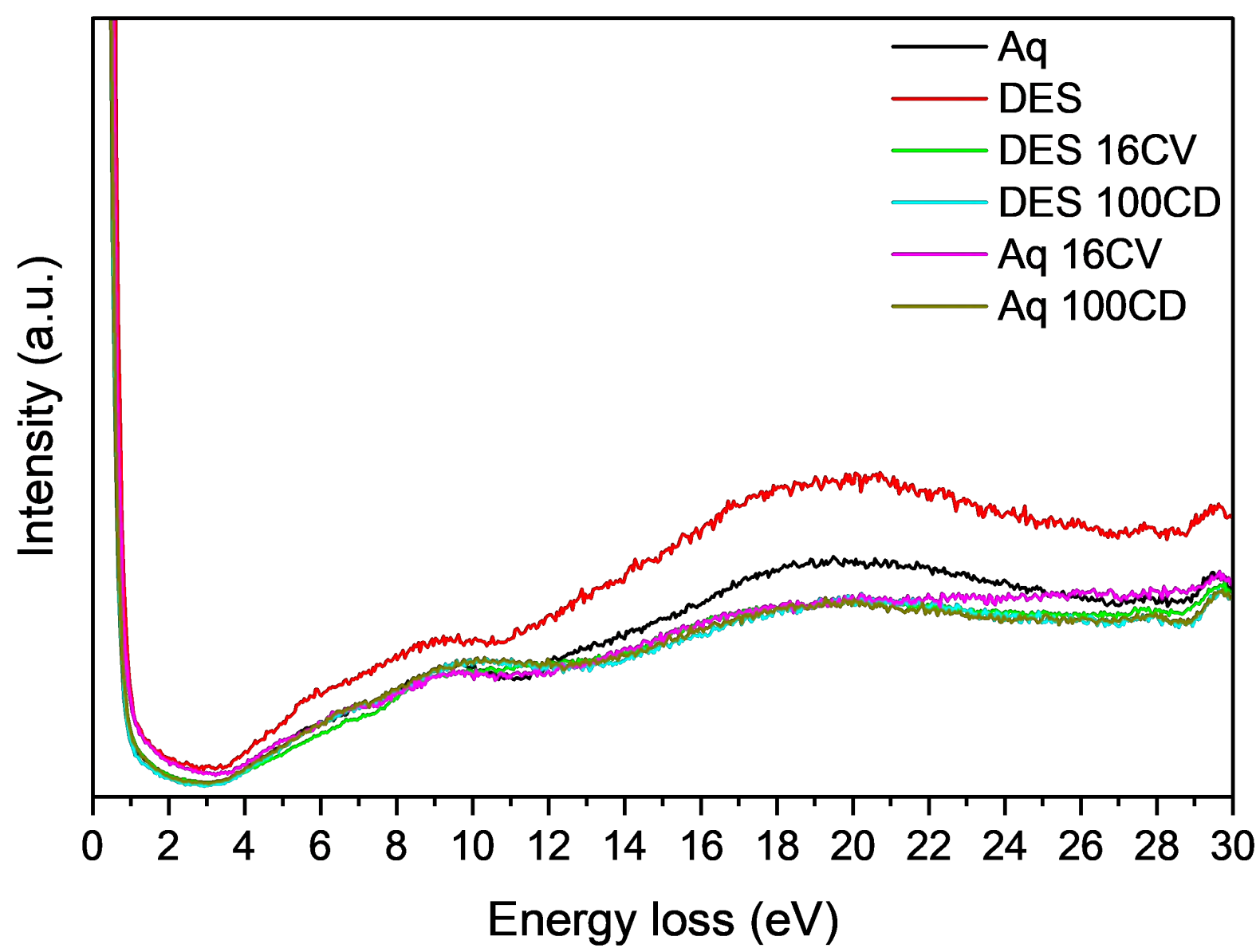

Fig. S5 Reflection electron energy loss spectroscopy (REELS) spectra of DES- and aqueous-derived Bi catalysts in the pristine state, after electrochemical activation (16CV), and after CO_2RR at 100 mA cm^{-2} (100CD). The spectra are plotted as intensity versus energy loss. The pristine DES-derived catalyst shows a higher plasmon-loss intensity, whereas the activated and post-CO_2RR samples exhibit similar spectral features, indicating comparable surface electronic structures after electrochemical activation.

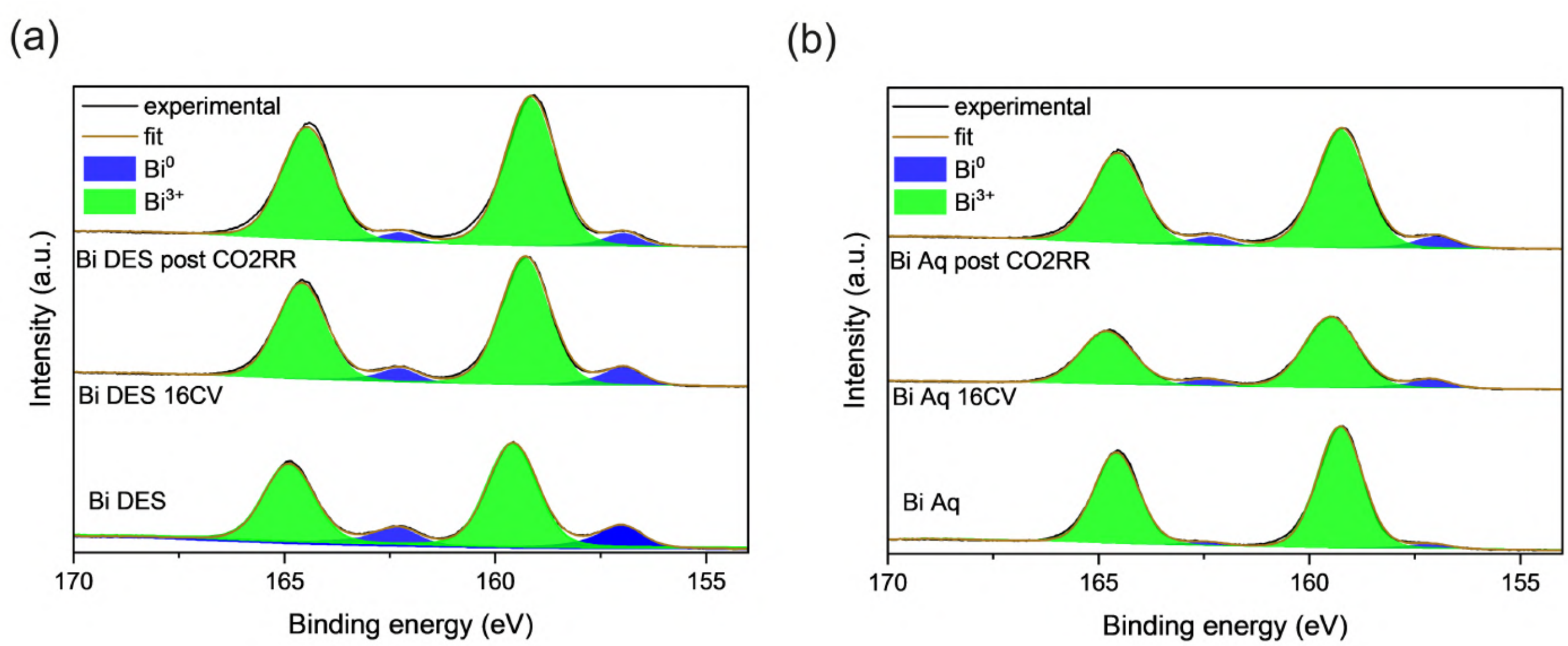

Fig. S6 High-resolution Bi 4f XPS spectra of (a) DES-derived and (b) aqueous-derived Bi electrodes in the pristine state, after electrochemical activation (16CV), and after CO_2RR at 100 mA cm^{-2} (post CO_2RR, denoted 100CD in Figure S9). The spectra are deconvoluted into metallic Bi^0 and oxidized Bi^{3+} doublets; binding energies are referenced to the Fermi edge. Fitted positions and Bi^0 fractions are given in Table S5.

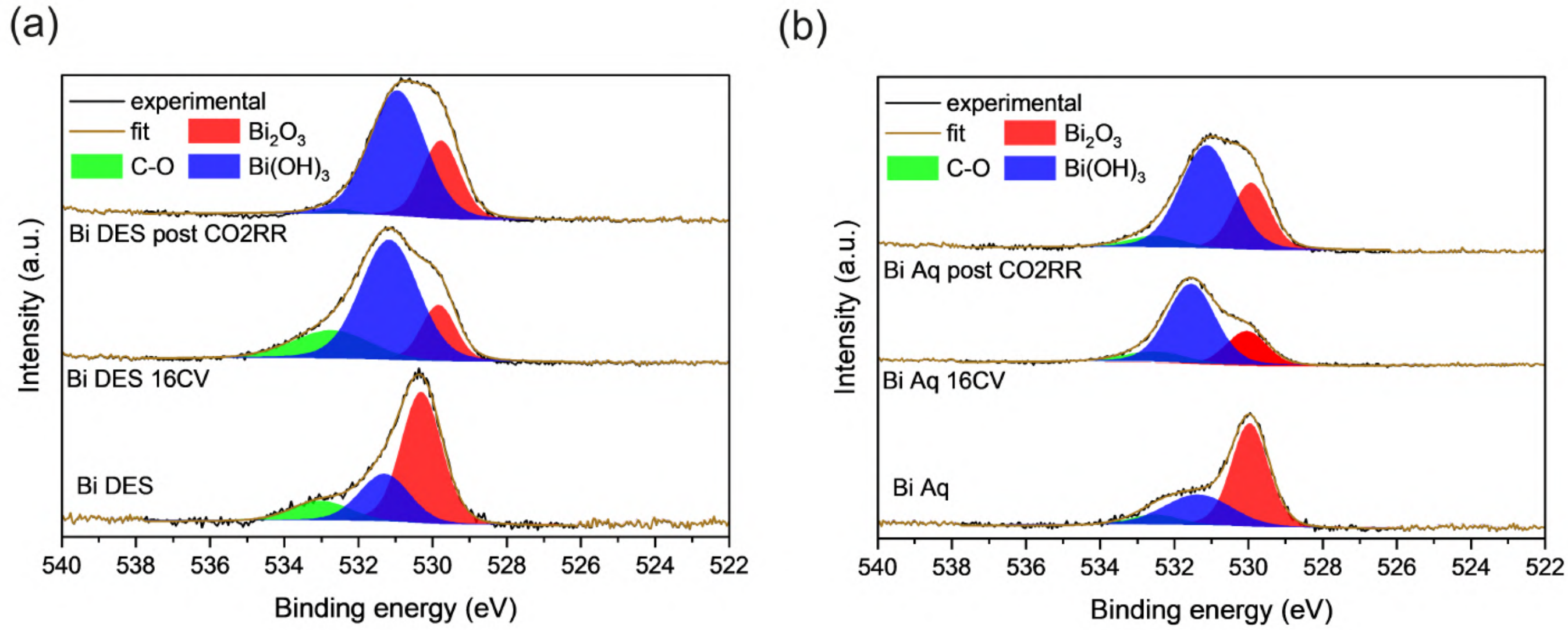

Fig. S7 High-resolution O 1s XPS spectra of (a) DES-derived and (b) aqueous-derived Bi electrodes in the pristine state, after activation (16CV), and after CO_2RR. The spectra are fitted with components for lattice oxygen in Bi_2O_3 (∼530 eV), Bi–OH species (∼531 eV), and C–O (∼532.5–533 eV).

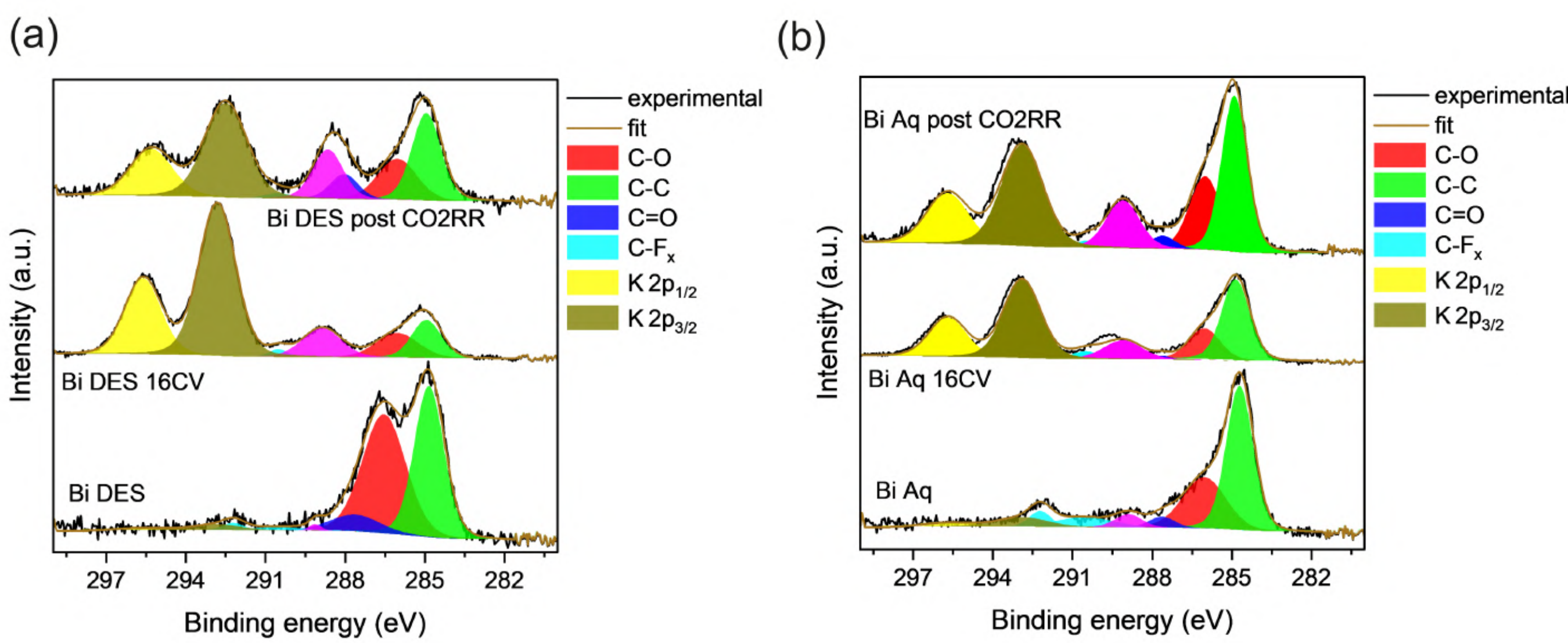

Fig. S8 High-resolution C 1s XPS spectra of (a) DES-derived and (b) aqueous-derived Bi electrodes in the pristine state, after activation (16CV), and after CO_2RR. The spectra are fitted with components corresponding to C–C, C–O, C=O, O–C=O (carbonate/carboxylate), and CF_x groups originating from the PTFE coating of the GDE. The K 2p doublet (K $2p_{3/2}$ at ∼292.9 eV) observed after activation and CO_2RR originates from residual $KHCO_3$ electrolyte.

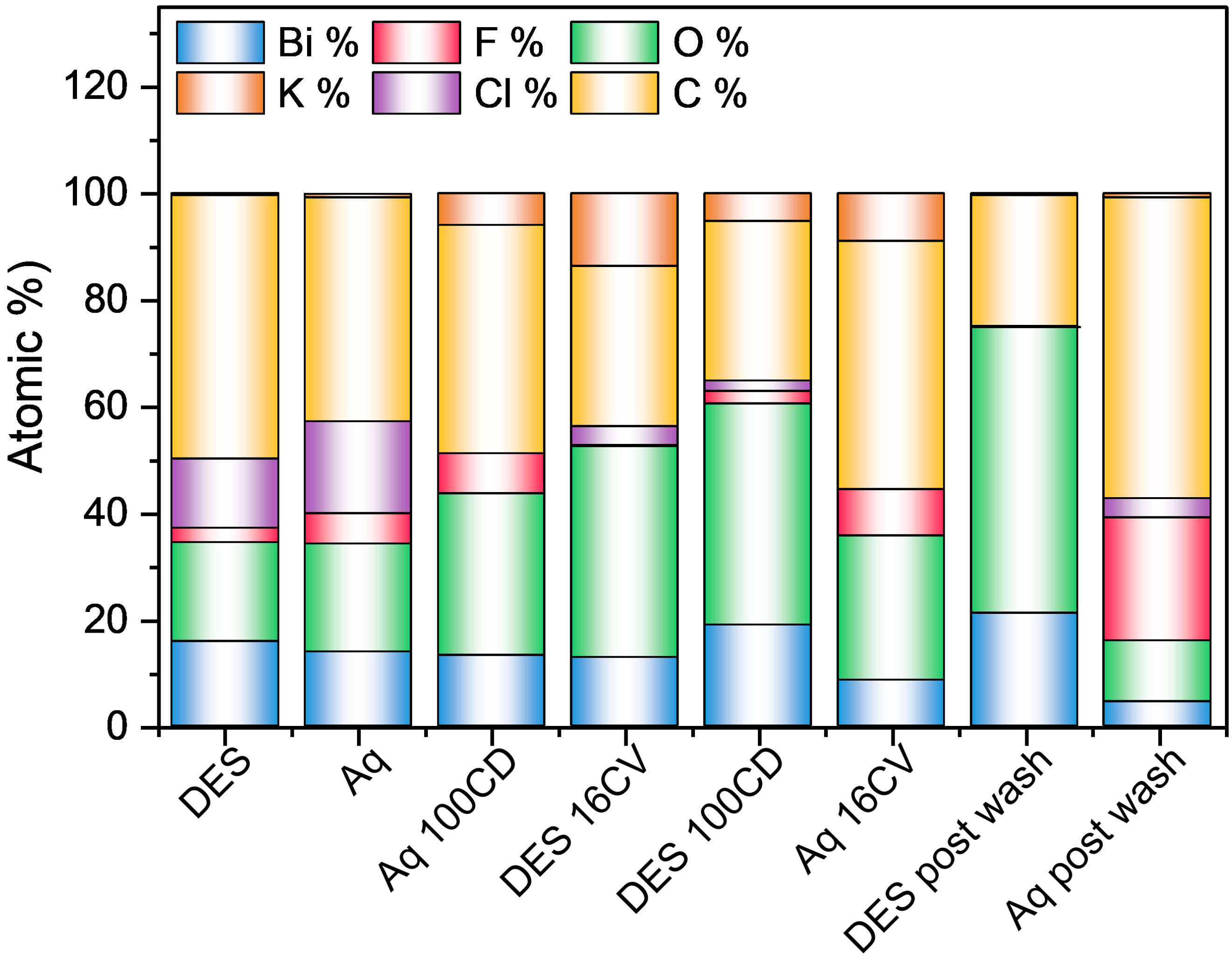

Fig. S9 XPS-derived surface elemental composition (at%) of DES- and aqueous-derived Bi electrodes in the pristine state, after activation (16CV), after CO_2RR at 100 mA cm^{-2} (100CD), and after flushing pristine electrodes with DI water in the flow cell for 3 h without applied potential (post wash).

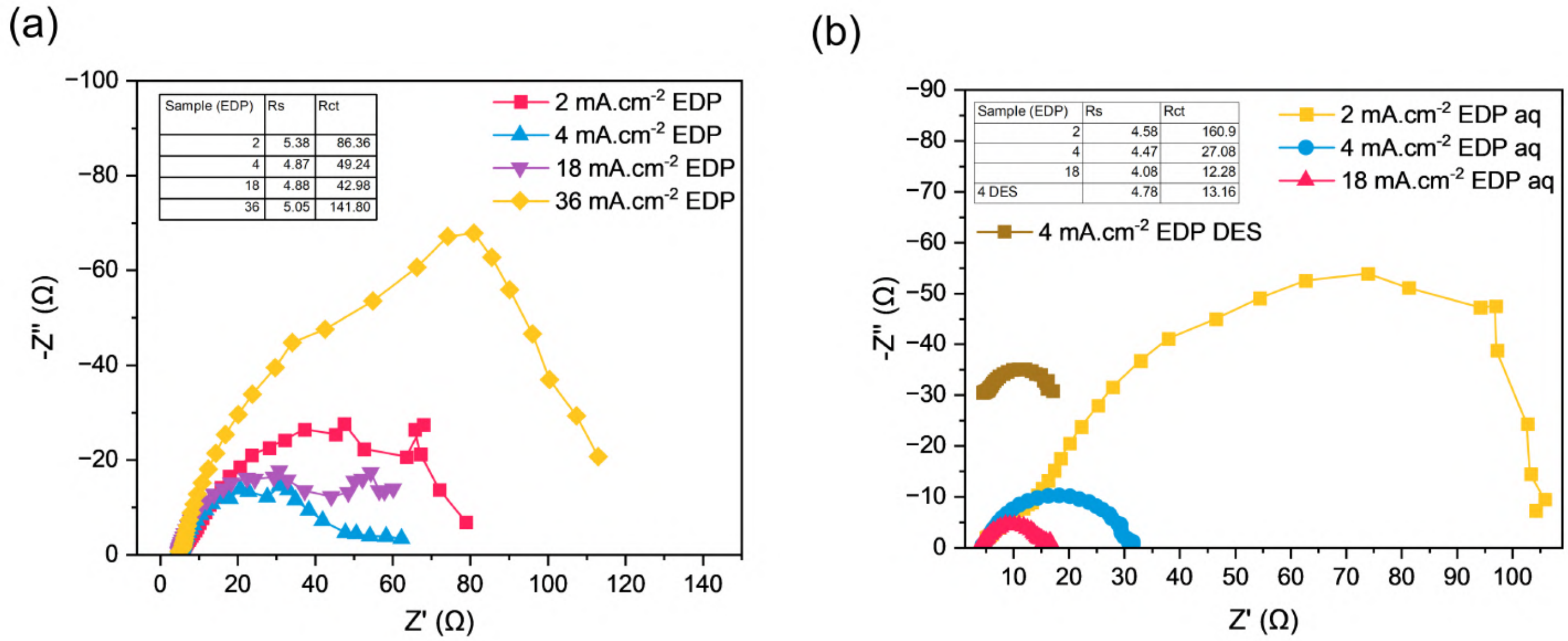

Fig. S10 Electrochemical impedance spectra of Bi catalysts after CV activation. Nyquist plots recorded in CO_2-saturated 0.5 M $KHCO_3$ at (a) −1.3 V and (b) −1.5 V vs. Ag/AgCl after 16 CV activation cycles. (a) DES-deposited Bi catalysts at deposition current densities of 2, 4, 18, and 36 mA cm^{-2}. (b) Aqueous-deposited Bi catalysts at 2, 4, and 18 mA cm^{-2}, together with the DES-deposited sample at 4 mA cm^{-2} for direct comparison. Inset tables report the solution resistance (R_s) and charge-transfer resistance (R_{ct}) extracted from equivalent circuit fitting. Spectra were recorded over a frequency range of 10 kHz to 0.01 Hz with an AC amplitude of 10 mV.

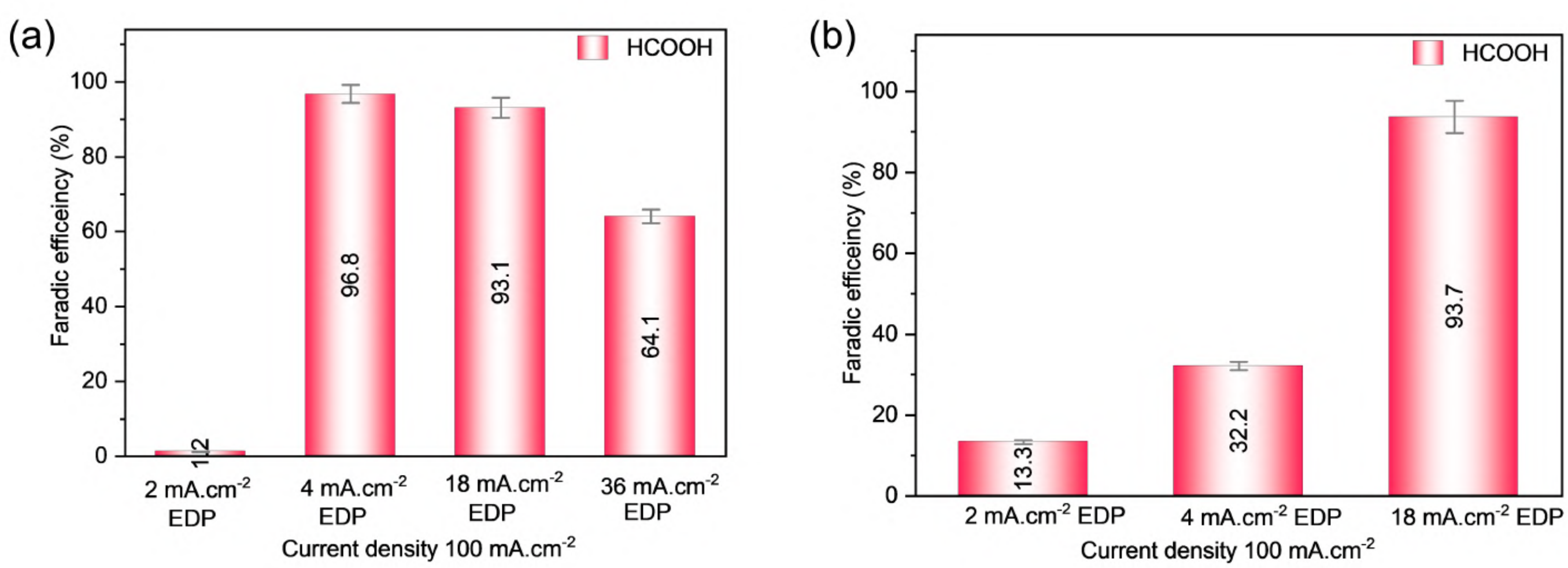

Fig. S11 Influence of electrodeposition current density on CO_2RR performance of Bi GDEs. Faradaic efficiency toward formic acid (FE_{HCOOH}) measured at 100 mA cm^{-2} for Bi electrodes deposited at different current densities in (a) DES electrolyte and (b) aqueous electrolyte.

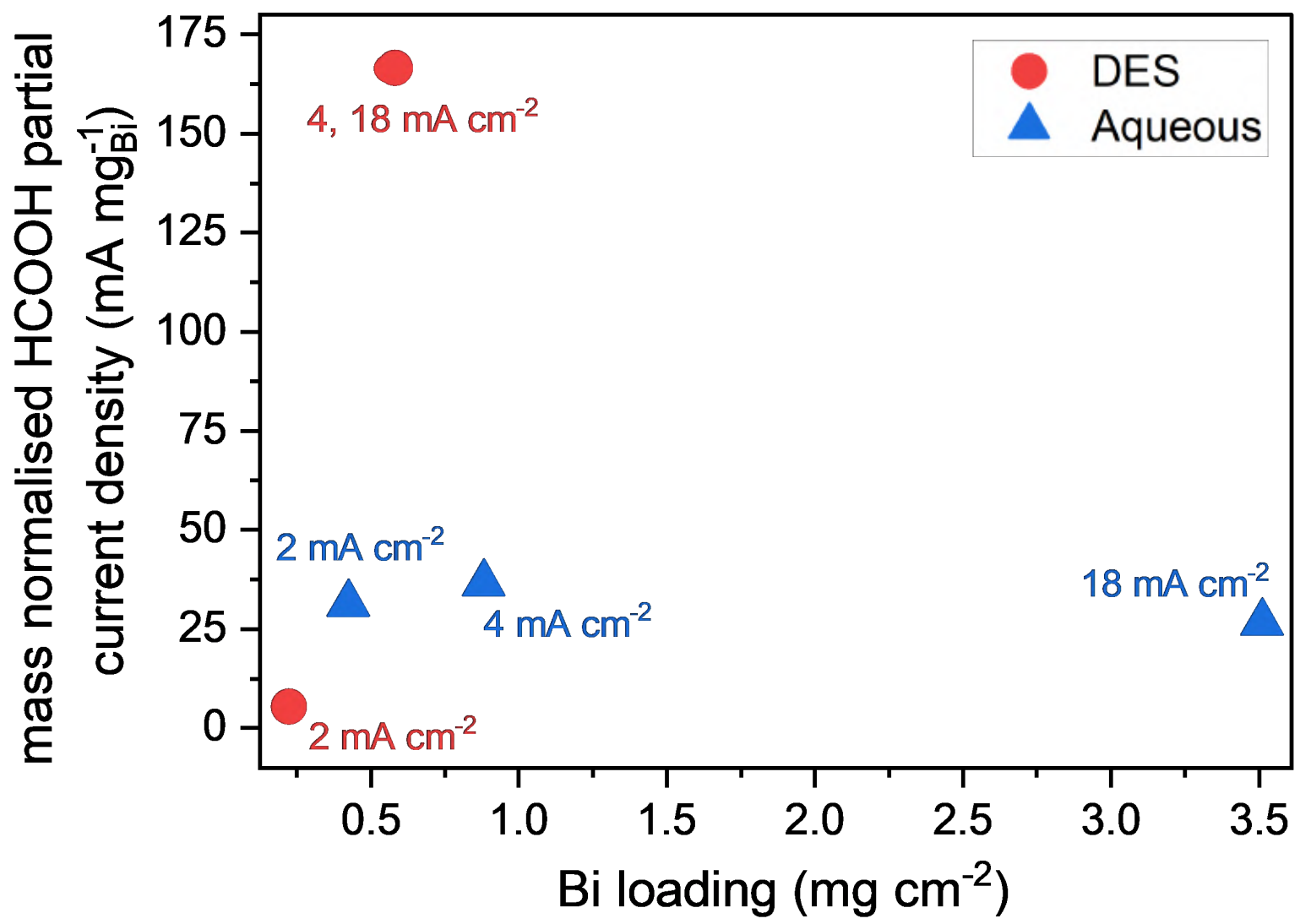

Fig. S12 Mass-normalised HCOOH partial current density as a function of Bi loading for DES- and aqueous-derived Bi/GDEs deposited at 2, 4, and 18 mA cm^{-2} (Table 1), evaluated at a CO_2RR test current density of 100 mA cm^{-2}. The DES-derived catalyst reaches its maximum mass-normalised activity ($\sim$166 mA mg$_{Bi}^{-1}$) already at 4 mA cm^{-2} deposition current and sustains this value up to 18 mA cm^{-2}, while the aqueous-derived catalyst remains below 37 mA mg$_{Bi}^{-1}$ across the entire deposition series.

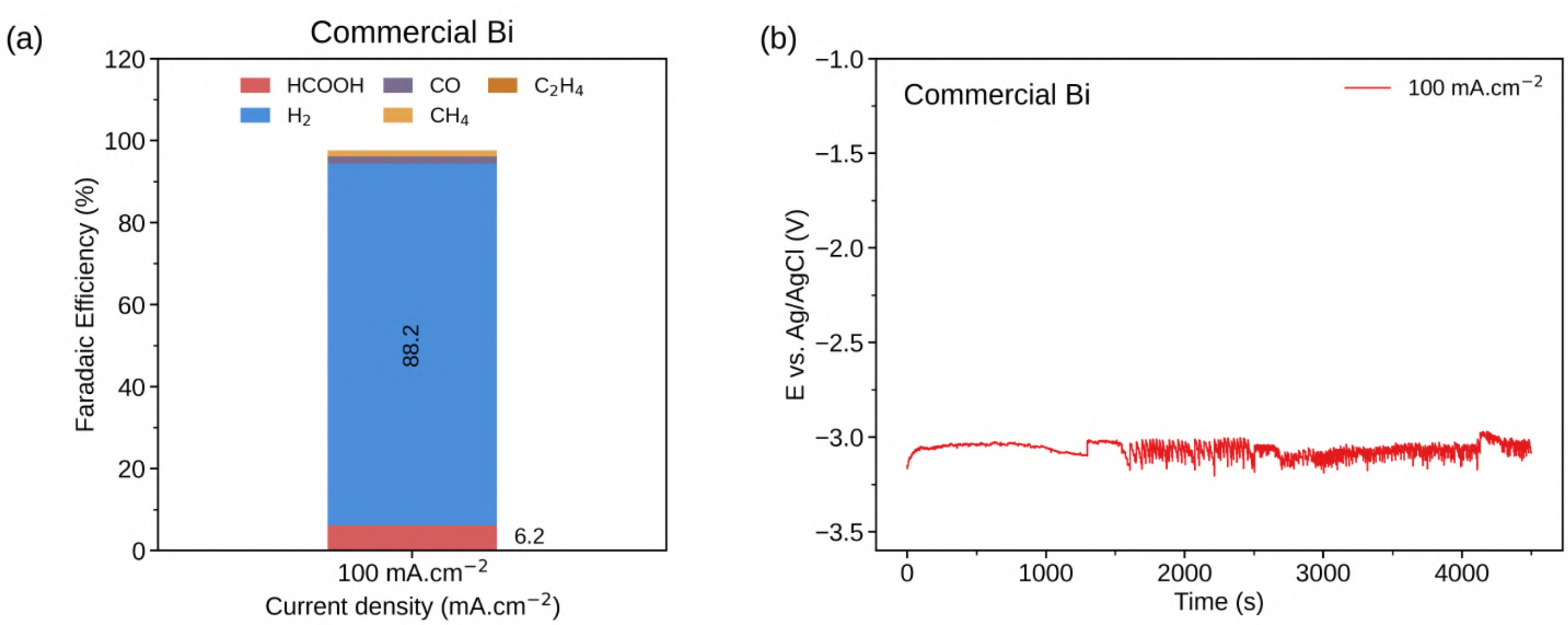

Fig. S13 CO_2RR performance of a benchmark electrode prepared from commercial Bi powder. (a) Faradaic efficiencies of HCOOH, H_2, CO, CH_4 and C_2H_4 at 100 mA cm^{-2} and (b) the corresponding chronopotentiometric profile, recorded in the flow cell under the same conditions as the electrodeposited Bi/GDEs. The electrode was prepared by air-brushing a catalyst ink onto a 2 cm^2 GDE to a Bi loading of 0.5 mg cm^{-2}, comparable to that of the DES-derived electrode. The ink consisted of 1.1 mg of Bi powder (325 mesh, 99.5%, metals basis, Thermo Scientific Chemicals), 10 µL of Sustainion® XA-9 alkaline ionomer and 1.5 mL of isopropanol, and was sonicated for 30 min before being sprayed with an airbrush using Ar as carrier gas. The electrode was dried for 5 h prior to testing. At 100 mA cm^{-2}, the air-brushed electrode yields a formate Faradaic efficiency of only 6.2%, with H_2 as the main product (88.2%), at a working potential of approximately -3.0 to -3.1 V vs. Ag/AgCl, compared with 96.8% formate Faradaic efficiency for the DES-derived electrode at a similar loading (Figure 3c).

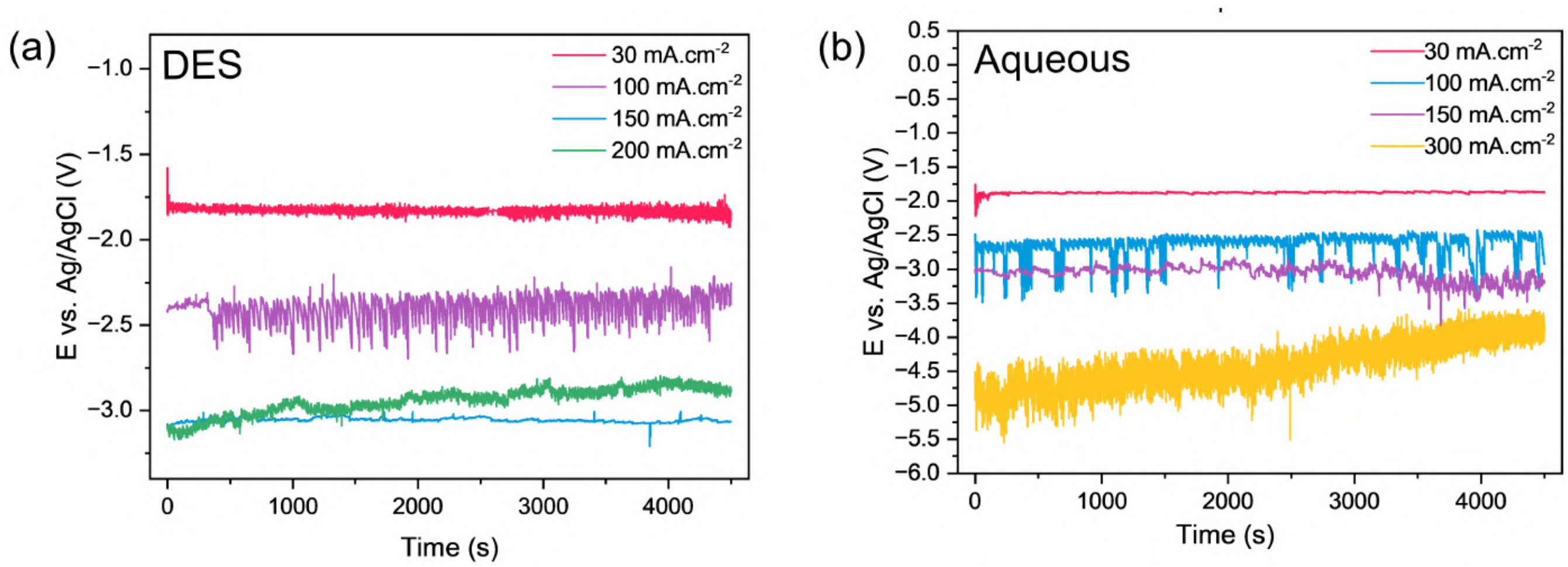

Fig. S14 Chronopotentiometric profiles of Bi catalysts during CO_2 electroreduction at different current densities. (a) DES-derived Bi catalyst and (b) aqueous-derived Bi catalyst, measured in the flow cell setup at TUW.

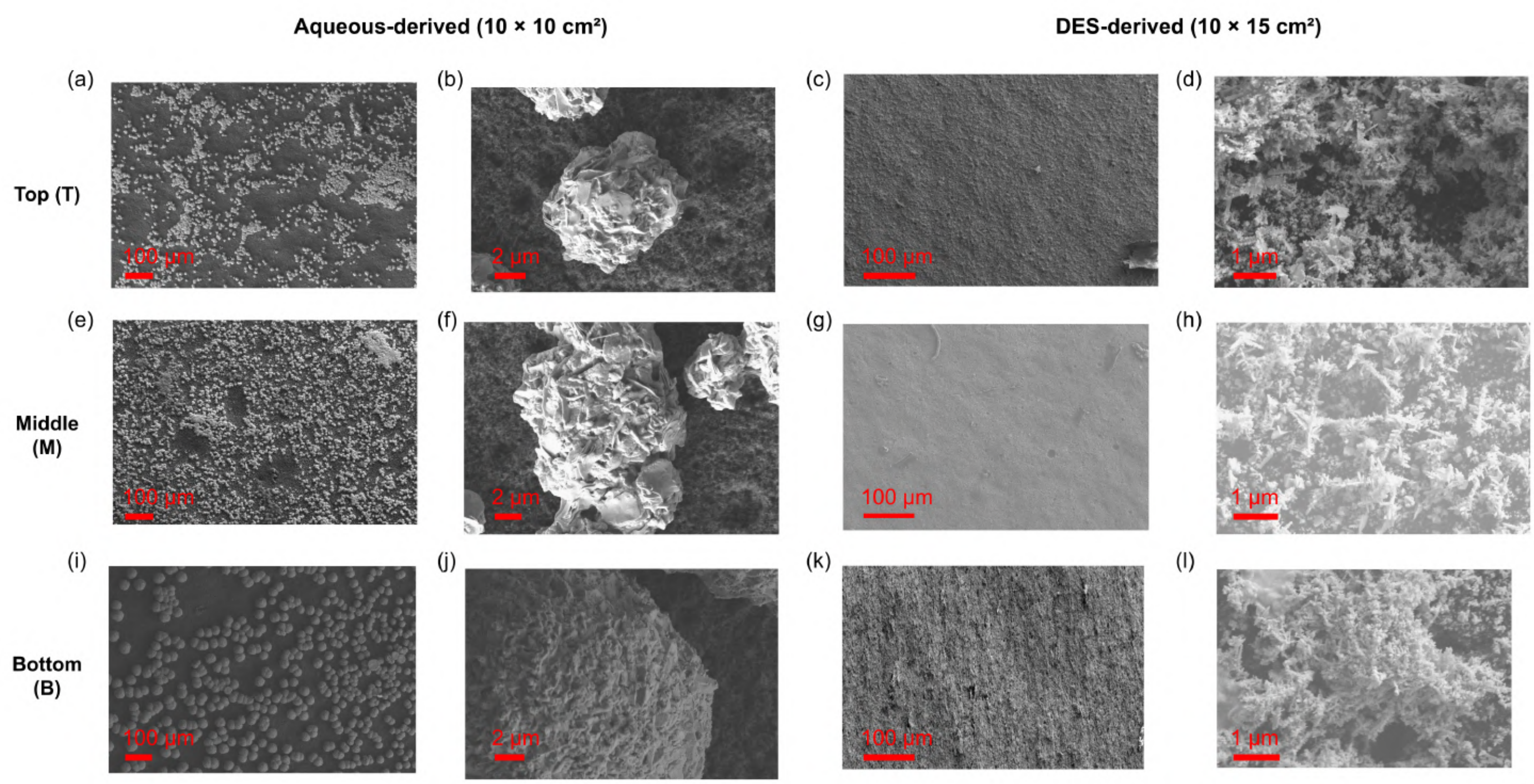

Fig. S15 SEM images of the large-area electrodes at the three sampling positions, top (T), middle (M) and bottom (B), marked in Figure 3e. (a,b,e,f,i,j) Aqueous-derived (electrodeposited at 18 mA cm^{-2}) electrode (10 × 10 cm^2; positions AT, AM and AB) and (c,d,g,h,k,l) DES-derived (electrodeposited at 4 mA cm^{-2}) electrode (10 × 15 cm^2; positions DT, DM and DB), each shown at low (a,c,e,g,i,k) and high (b,d,f,h,j,l) magnification. The aqueous-derived electrode shows a pronounced gradient in Bi coverage along the electrode, from sparsely distributed small aggregates at the top position to densely packed, large spherical aggregates at the bottom position. The DES-derived electrode shows a continuous and homogeneous Bi coating with a similar nanostructured morphology at all three positions.

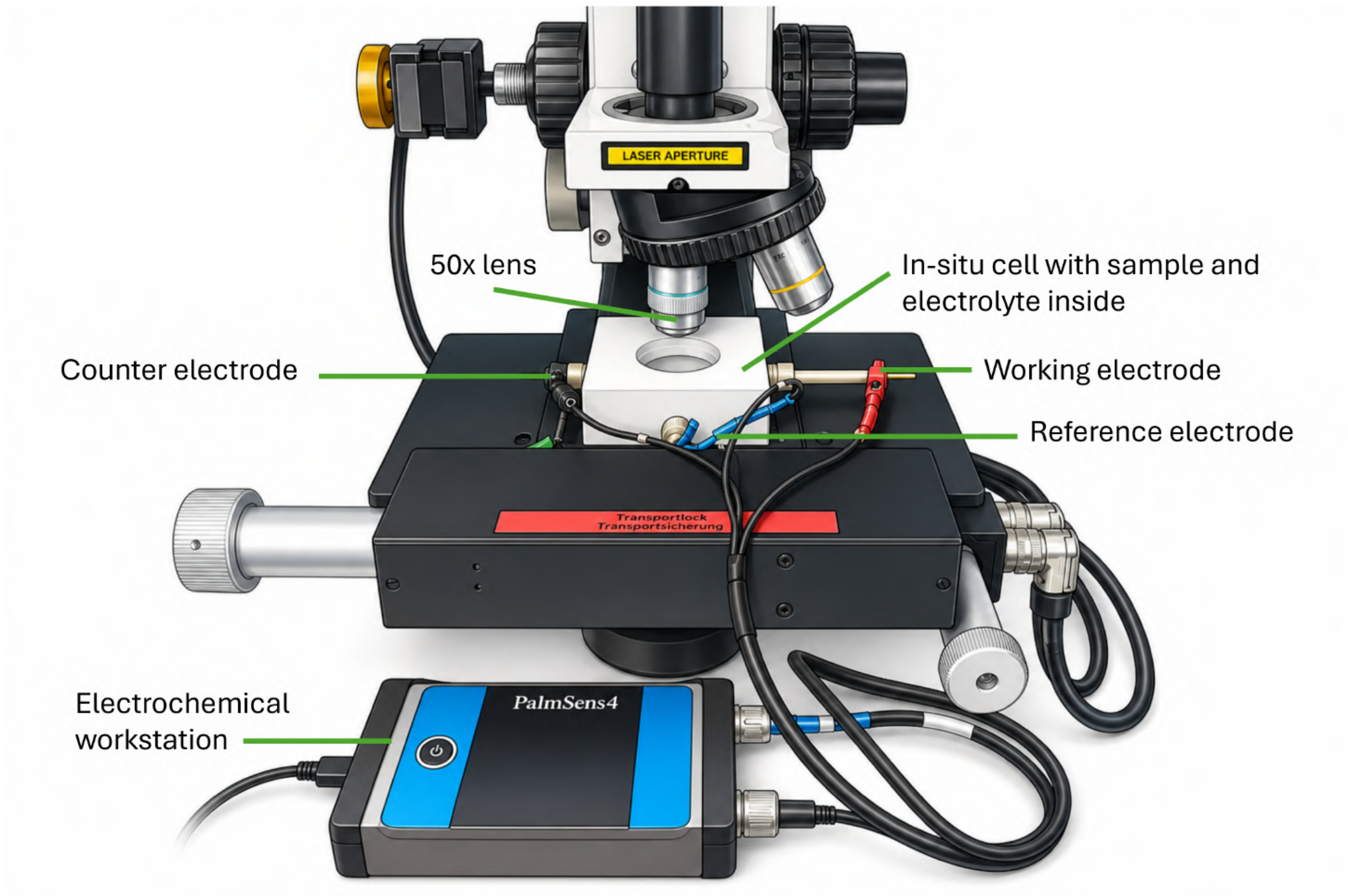

Fig. S16 *In situ* Raman electrochemical cell used for operando measurements with the three-electrode configuration consisting of a working electrode, Pt counter electrode, and reference electrode (Ag/AgCl).

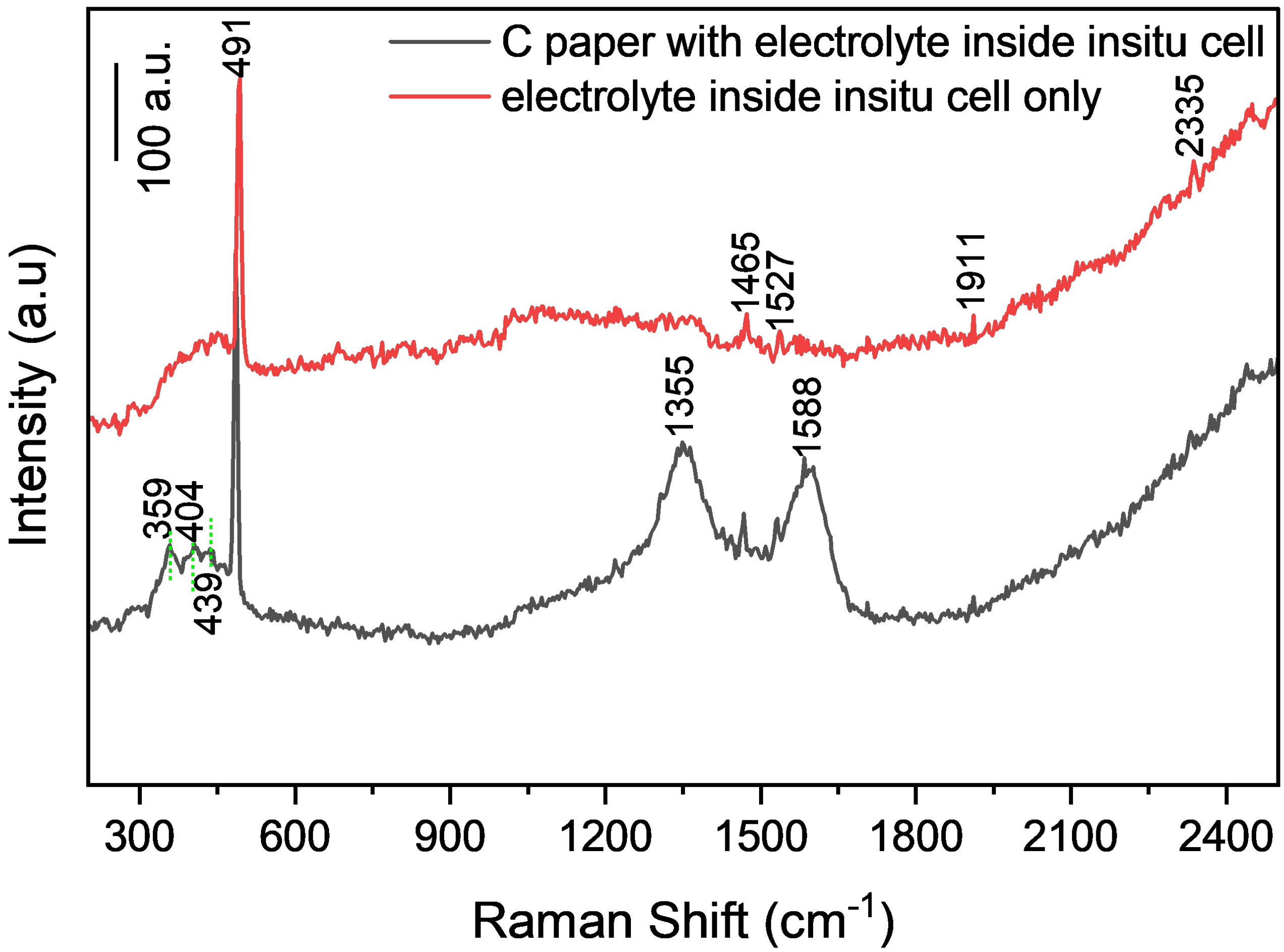

Fig. S17 *In situ* background spectra (raw data) recorded for electrolyte only and electrolyte with carbon paper only, confirming the absence of Bi-related or carbonate features in the substrate and electrolyte signals.

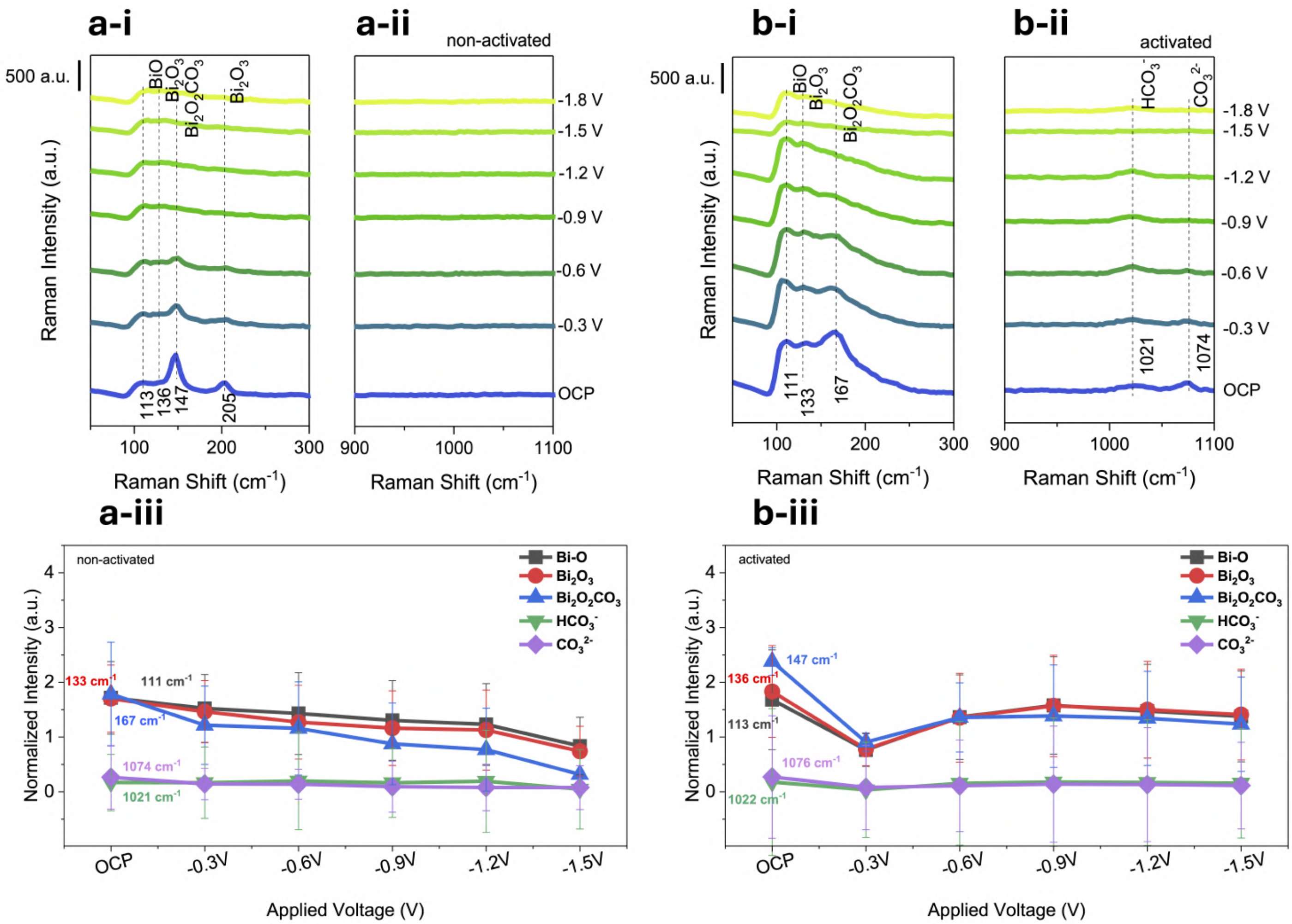

Fig. S18 Operando Raman spectra of the aqueous-derived catalyst before (a) and after (b) electrochemical activation. (a-i, b-i) Low-wavenumber region (50–300 cm^{-1}) showing the potential-dependent evolution of Bi^0, Bi–O, Bi_2O_3, and $Bi_2O_2CO_3$ bands. (a-ii, b-ii) Carbonate region (900–1100 cm^{-1}) showing the HCO_3^- and CO_3^{2-} bands. (a-iii, b-iii) Corresponding normalized band intensities as a function of applied potential, non-activated (a-iii) and activated (b-iii). This figure mirrors **Figure 4** of the main text, which shows the equivalent data for the DES-derived catalyst.

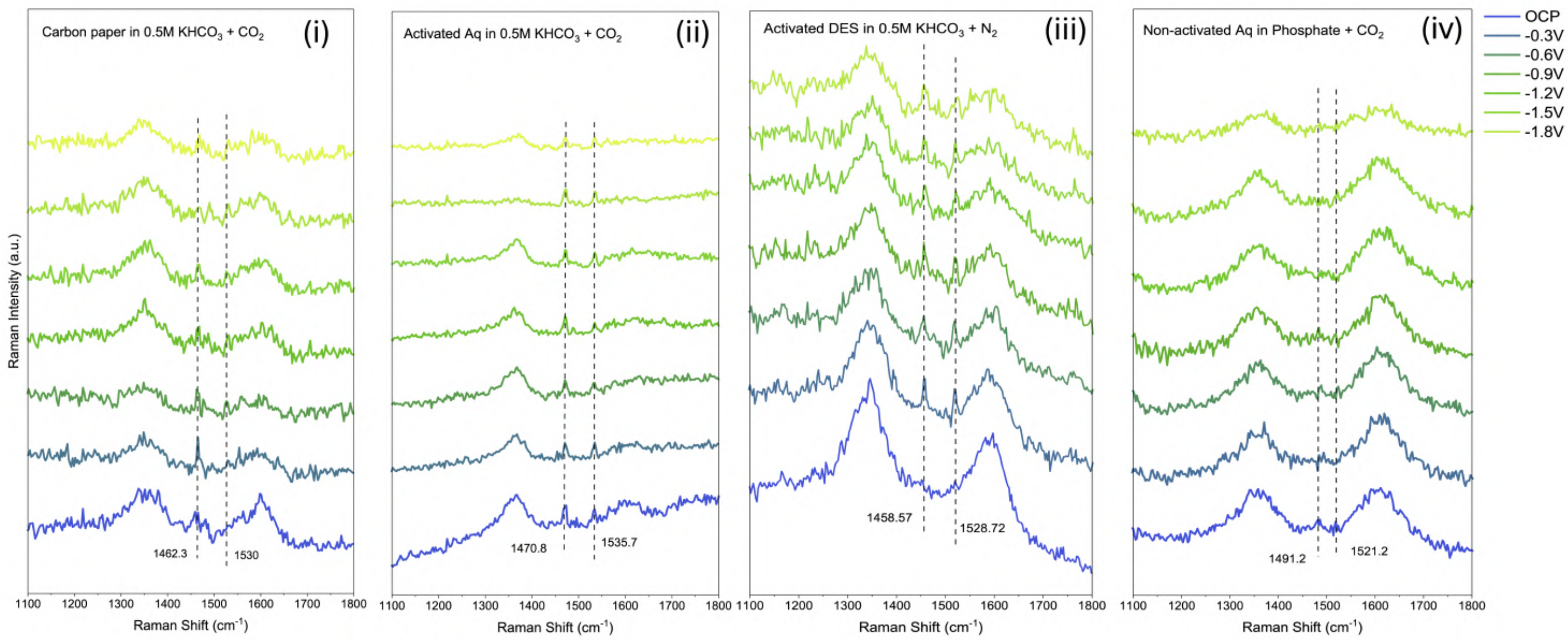

Fig. S19 Control experiments probing the origin of the 1400–1600 cm^{-1} spectral region. *In situ* Raman spectra recorded from OCP to −1.8 V vs. Ag/AgCl for (i) bare carbon paper in CO_2-saturated 0.5 M $KHCO_3$, (ii) the activated aqueous-derived catalyst in CO_2-saturated 0.5 M $KHCO_3$, (iii) the activated DES-derived catalyst in N_2-purged 0.5 M $KHCO_3$, and (iv) the non-activated aqueous-derived catalyst in CO_2-saturated phosphate electrolyte. Features within 1458–1536 cm^{-1} are observed under all four conditions, including on bare carbon paper and under N_2 purging (i.e. in the absence of CO_2), indicating that this region is not specific to CO_2 electroreduction or to the Bi catalyst and overlaps with the D/G envelope of the carbon-paper support.

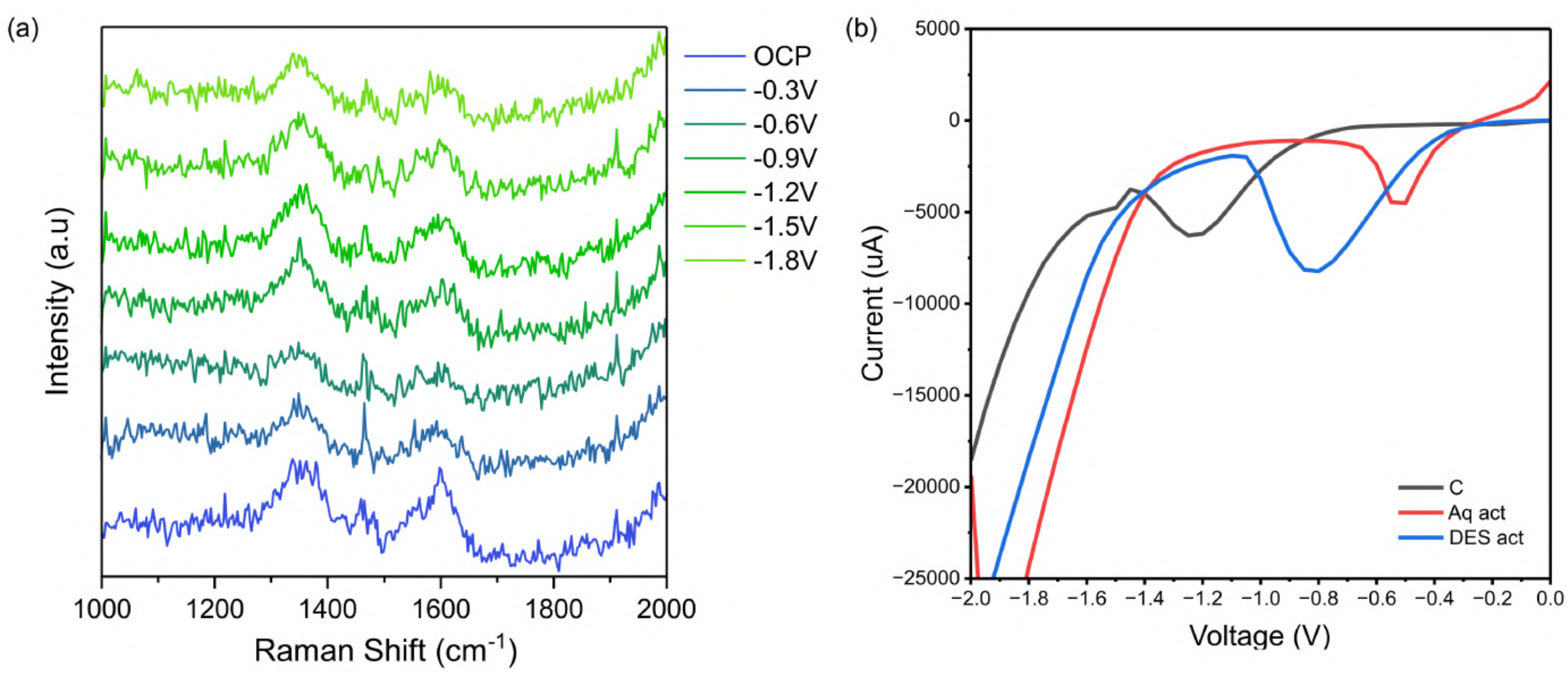

Fig. S20 *In situ* Raman spectra (raw data) recorded for bare carbon paper (a) and linear sweep voltammograms (LSV) of activated aqueous- and DES-derived catalysts (b).

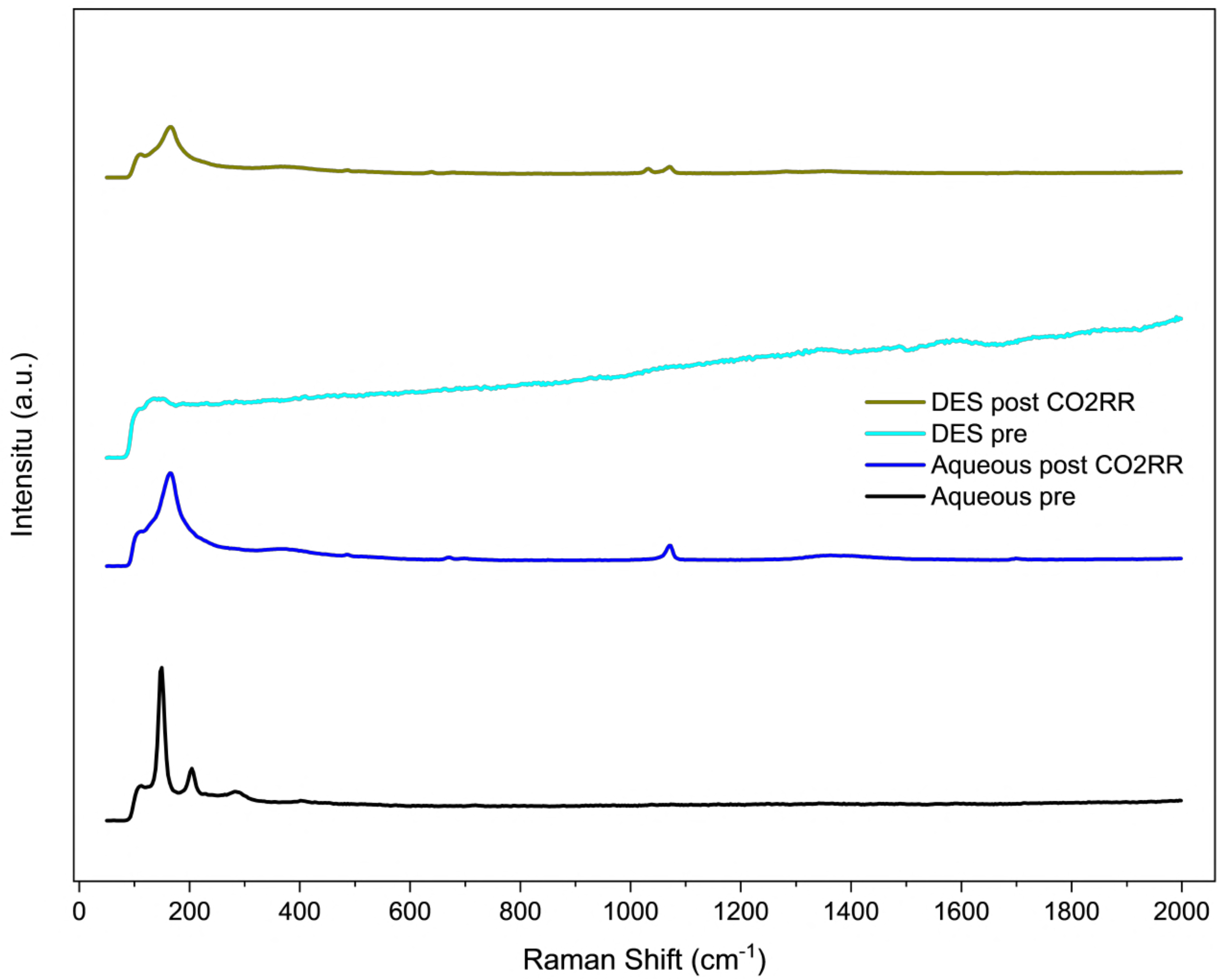

Fig. S21 *Ex situ* Raman spectra measured in air for aqueous- and DES-derived catalysts before and after cyclic voltammetry (CV) and chronopotentiometry (CD) activation, prior to baseline subtraction.

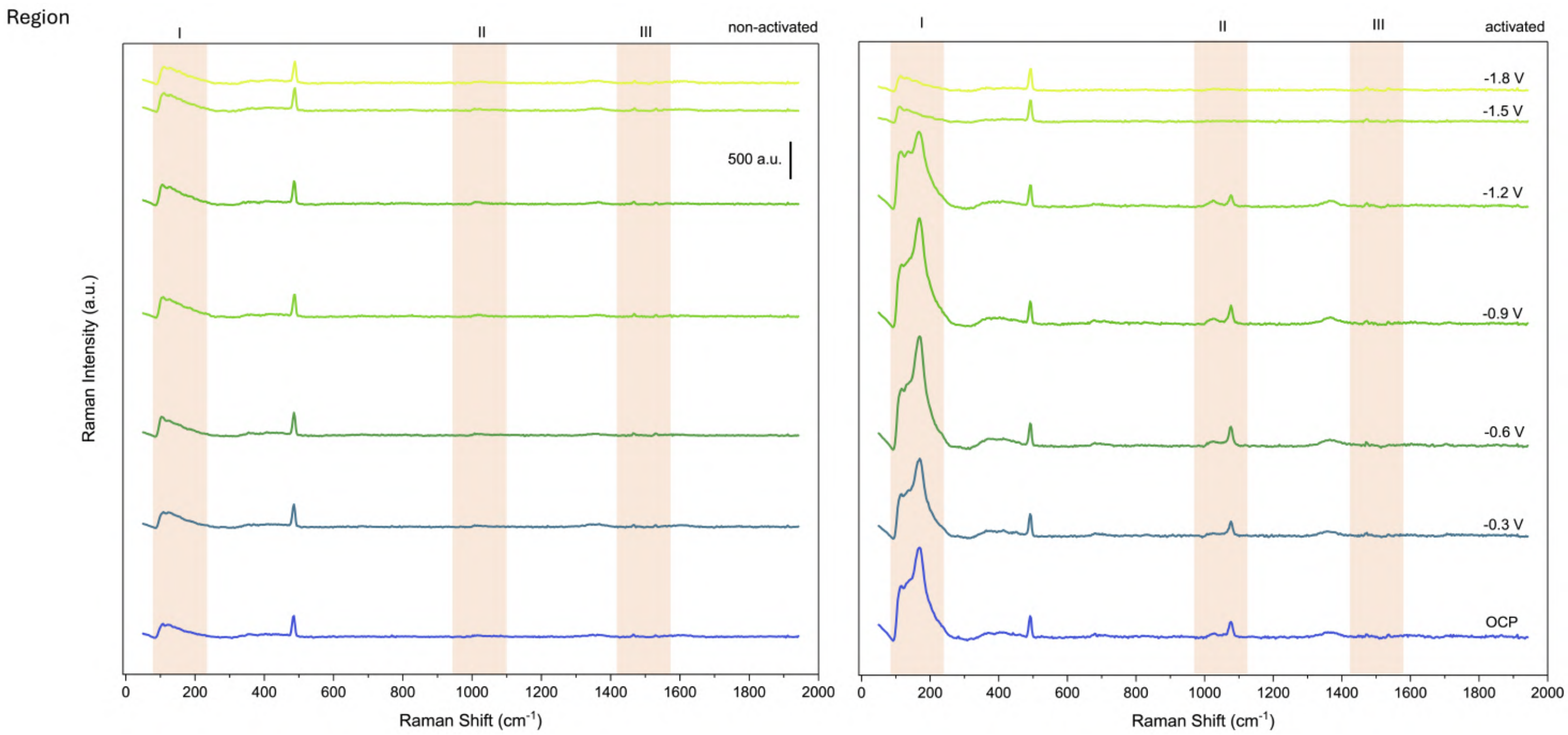

Fig. S22 Full-range *in situ* Raman spectra (50–2000 cm^{-1}) of the DES-derived catalyst before (left, non-activated) and after (right, activated) electrochemical activation, recorded from OCP to −1.8 V vs. Ag/AgCl in CO_2-saturated 0.5 M $KHCO_3$. Shaded bands mark region I (Bi-related low-wavenumber modes, 50–250 cm^{-1}), region II (interfacial carbonate modes, 970–1130 cm^{-1}), and region III (1420–1580 cm^{-1}), the latter excluded from mechanistic analysis as discussed in the main text and in **Figure S19**.

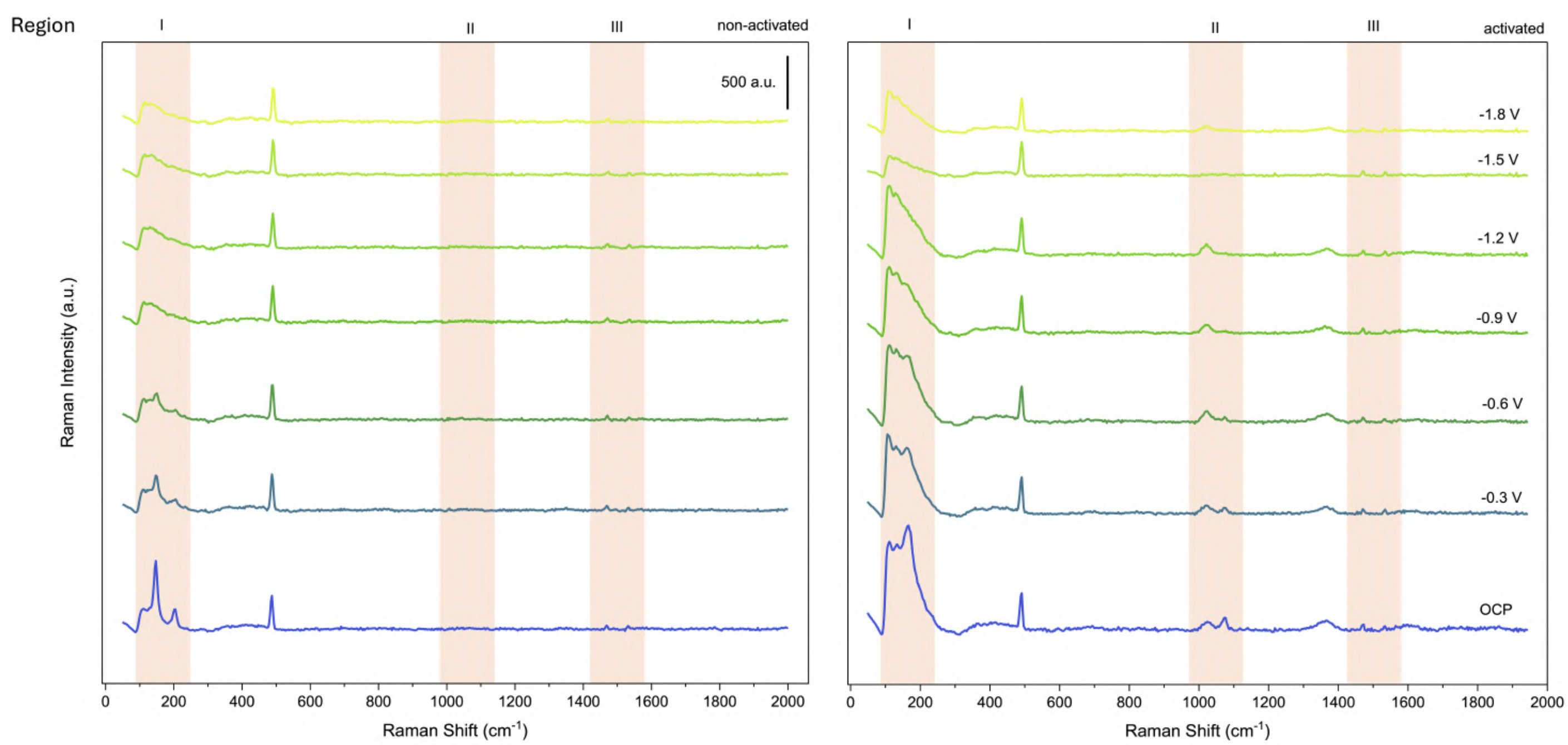

Fig. S23 Full-range *in situ* Raman spectra (50–2000 cm^{-1}) of the aqueous-derived catalyst before (left, non-activated) and after (right, activated) electrochemical activation, recorded from OCP to −1.8 V vs. Ag/AgCl in CO_2-saturated 0.5 M $KHCO_3$. Shaded bands mark region I (Bi-related low-wavenumber modes, 50–250 cm^{-1}), region II (interfacial carbonate modes, 970–1130 cm^{-1}), and region III (1420–1580 cm^{-1}), the latter excluded from mechanistic analysis as discussed in the main text and in **Figure S19**.

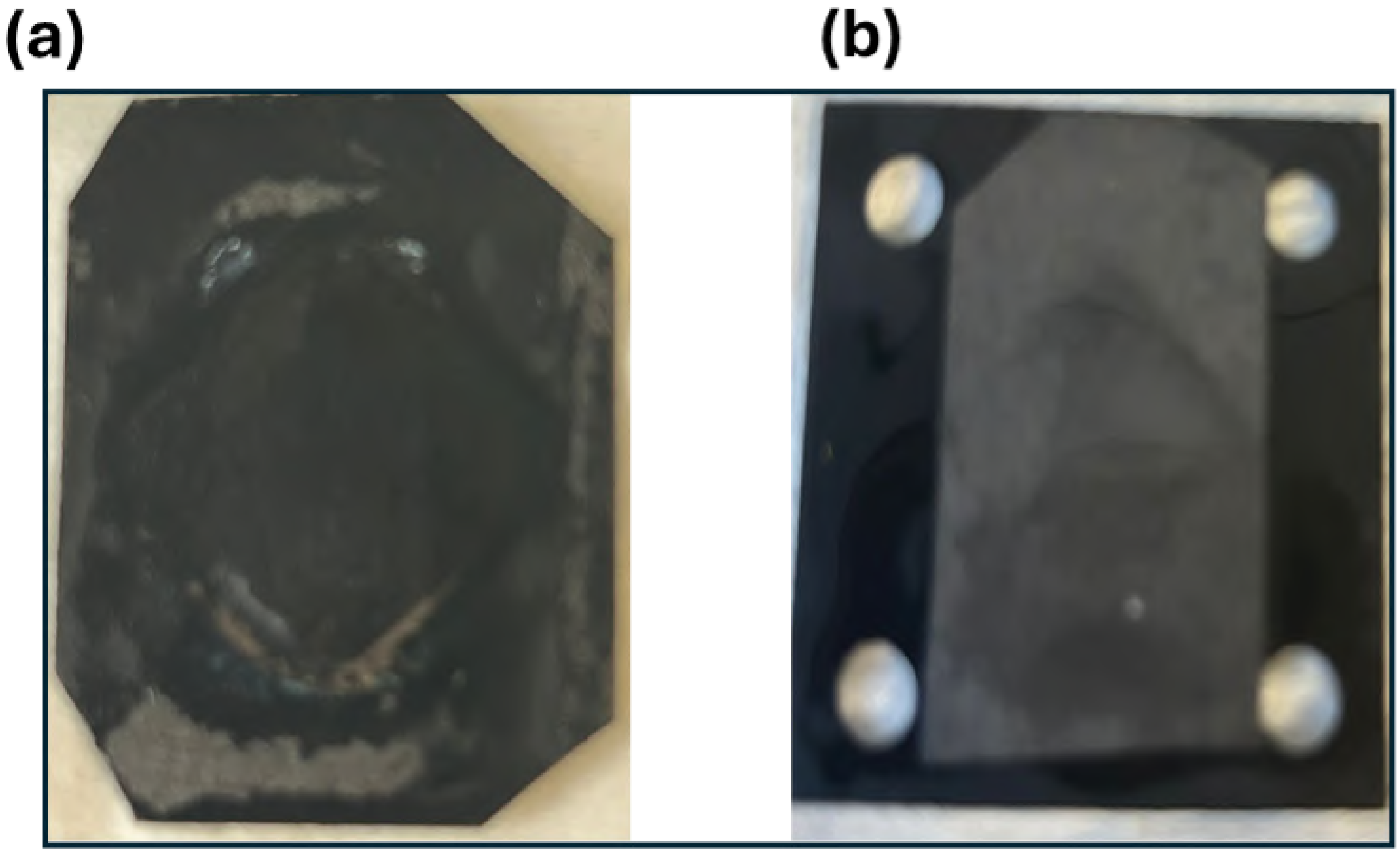

Fig. S24 Digital photographs of the DES-derived electrode after 16 h electrolysis stability test. (a) Catalyst side and (b) gas side of the gas diffusion electrode.

Table S1 Summary of Raman spectroscopic studies on Bi-based catalysts for CO_2-to-formate conversion.

Spectroscopy	Catalyst Precursor	Key Raman Peaks (cm^{-1}) and Assignments	Main Finding	Ref
In situ/operando and *ex situ*	Bi_2O_3 foam	119, 313, 475 (α-Bi_2O_3); 162 ($(BiO)_2CO_3$); 70, 98 (Bi^0)	Identified 162 cm^{-1} sub-carbonate as key intermediate	Dutta 2021 (ACS Catal.)[1]
In situ/operando and *ex situ*	Bi_2O_3 on GDE	162, 163, 96, 313 ($(BiO)_2CO_3$); Bi^0, Bi_2O_3	Showed CO_2 stabilizes sub-carbonate to -1.5 V	Zelocualtecatl-Montiel 2022 (ACS Catal.)[2]
Ex situ	Bi NSs, Bi_2O_3	74, 99.7 (Bi^0); 119, 313 (Bi_2O_3); 162 ($(BiO)_2CO_3$)	Argued Bi^0 is active site; sub-carbonate transient	Wu 2022 (I&EC Res.)[10]
In situ/operando and *ex situ*	Bi_2S_3 nanorods	184, 237, 259 (Bi_2S_3); 69, 95, 168 (post-reaction)	Identified Bi^0 cluster/$Bi_2O_2CO_3$ nanocomposite; detected *OCHO at 1469 cm^{-1}	Lin 2023 (Angew. Chem.)[11]
In situ/operando and *ex situ*	Bi@Bi_2O_3 nanodendrites	313 (Bi_2O_3); 1469 (*OCHO); 1130 ($HCOO^-$)	First direct detection of OCHO intermediate at 1469 cm^{-1}	Yang 2023 (Adv. Funct. Mater.)[12]
Ex situ	Bi-TA complex	568 (Bi–TA); 93, 120, 424 (post-reaction)	Bi-TA precursor $\rightarrow$ Bi/$Bi_2O_2CO_3$; Raman confirms 93, 120, 424 cm^{-1} as $Bi_2O_2CO_3$ fingerprint	Li 2025 (Adv. Sci.)[13]
**Operando/*in situ***	Bi on carbon paper (from wires/particles) to flower nanostructures	106 (Bi–Bi, Bi^0)	Becomes dominant as the Bi–O and $Bi_2O_2CO_3$ bands decrease at strongly cathodic potentials	Steele 2014 (Opt. Mater. Express)[14]
		111, 113, 114, 128 (Bi–O, Bi_2O_3)	Persists at moderate cathodic potentials and decreases sharply at -1.5 V	Jia 2023 (J. Am. Chem. Soc.)[15]
		133, 136, 138 (Bi–O, Bi_2O_3)	Decreases with cathodic polarization, consistent with Bi_2O_3 reduction	Steele 2014 (Opt. Mater. Express)[14]
		147, 150, 167, 170 (Bi–O, $Bi_2O_2CO_3$)	Decreases rapidly for Aq/Aq-act; for DES/DES-act, increases transiently up to -0.6 V and decreases sharply beyond -1.2 V	Zhang 2025 (Chem. Sci.)[16]
		205 (Bi–O, Bi_2O_3)	Progressively attenuates during cathodic polarization	Zhang 2025 (Chem. Sci.)[16]
		487 (Bi–O, Bi_2O_3)	Weakens and becomes unresolved as the oxide phase is reduced	Steele 2014 (Opt. Mater. Express)[14]
		1021, 1025 (C–O, HCO_3^-)	Relative intensity tracked together with the CO_3^{2-} band to follow the interfacial carbonate equilibrium	Jeon 2025 (Chem. Eng. J.)[17]
		1074, 1077 (C–OH, CO_3^{2-})	Non-monotonic evolution for DES-act, decreasing beyond -0.9 V; comparatively small changes for Aq-act	Jeon 2025 (Chem. Eng. J.)[17]
		1366 (Carbon, D band)	Present in the carbon-paper control; not assigned to catalyst restructuring or adsorbed intermediates	Jawhari 1995 (Carbon)[18]
		1468, 1475 (O–C–O, monodentate CO_3^{2-})	Potential-dependent evolution cannot be reliably resolved because of overlap with the carbon D/G-band envelope	Jia 2023 (J. Am. Chem. Soc.)[15]
		1528, 1533, 1538 (O–C–O, CO_3^{2-})	Potential-dependent evolution cannot be reliably resolved because of the strong carbon-support contribution	Jia 2023 (J. Am. Chem. Soc.)[15]
		1600 (Carbon, G band)	Present in the carbon-paper control; not interpreted as a catalyst- or intermediate-related feature	Jawhari 1995 (Carbon)[18]

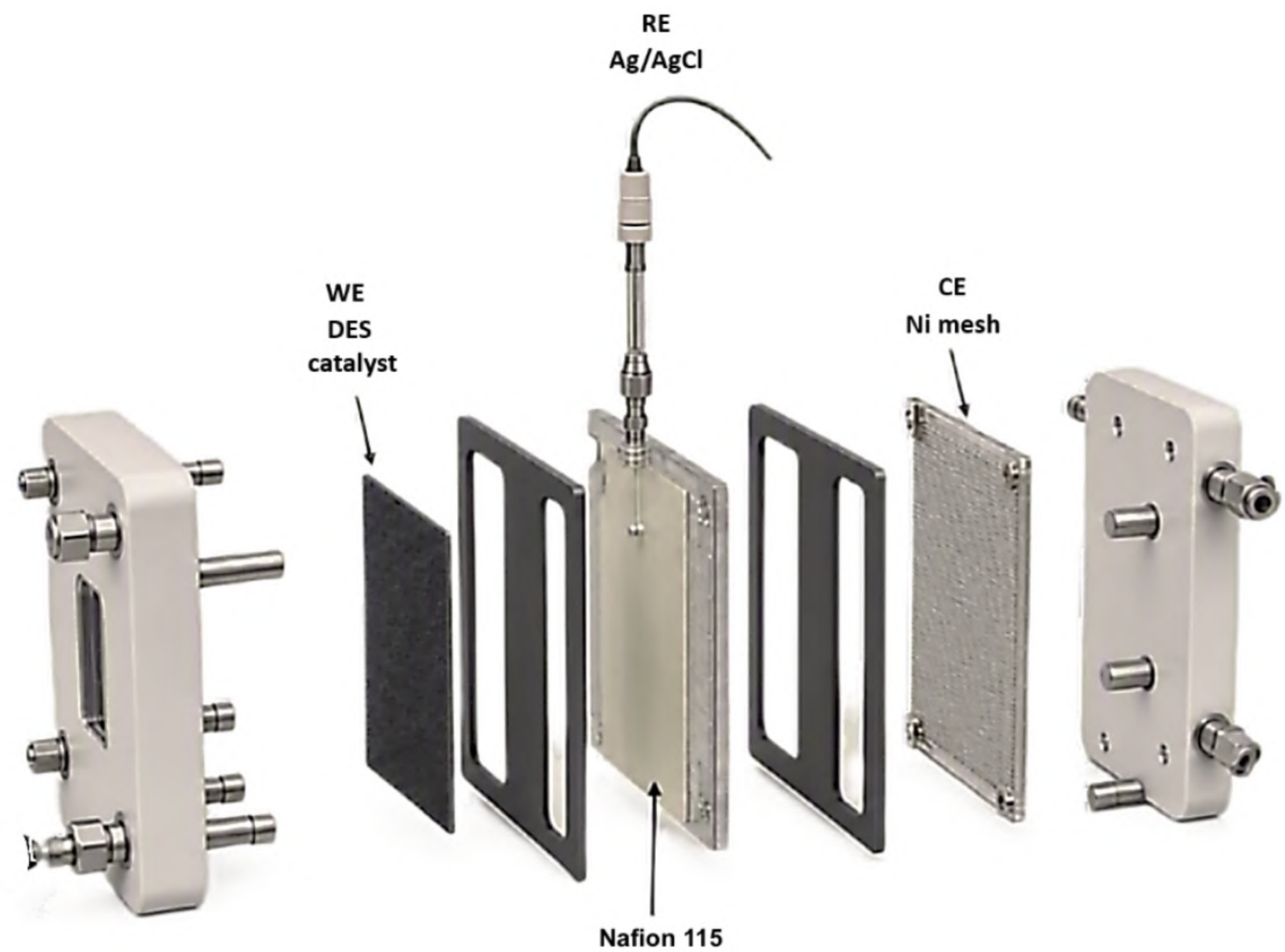

Fig. S25 Exploded schematic of the flow-cell configuration used for the long-term CO_2RR stability measurements, employing the directly electrode-posited DES-Bi/GDE (1×1 cm^2) as working electrode, an Ag/AgCl reference electrode, a Ni foam counter electrode, and a Nafion 115 membrane.

Table S2 Double-layer capacitance (C_{dl}) values extracted from the scan rate dependence of the non-faradaic current for aqueous- and DES-deposited Bi catalysts at different deposition current densities.

Sample	Slope (F)	C_{dl} (mF)
Aq-2 mA cm^{-2}	2.80×10^{-3}	2.80
Aq-4 mA cm^{-2}	2.19×10^{-3}	2.19
Aq-18 mA cm^{-2}	3.87×10^{-3}	3.87
DES-2 mA cm^{-2}	6.20×10^{-4}	0.620
DES-4 mA cm^{-2}	1.43×10^{-3}	1.43
DES-18 mA cm^{-2}	8.04×10^{-3}	8.04

Table S3 Bismuth dissolution during electrochemical treatments quantified by ICP–MS. DES and Aq denote catalysts electrodeposited from deep eutectic solvent and aqueous electrolytes at 18 mA cm^{-2}, respectively. CP20: chronopotentiometry at 100 mA cm^{-2} for 4500 s without prior CV activation. 16CV: number of activation cycles prior to electrolysis. 100CD: CV activation followed by chronopotentiometry at 100 mA cm^{-2} for 4500 s.

Sample	**Initial loading** (mg cm^{-2})	**Bi dissolved** (mg L^{-1})	**Bi dissolved** (mg)	**Bi dissolved** (mg cm^{-2})	**Bi loss** (%)
DES-16CV	0.5599	0.46	0.0230	0.0115	2.05
Aq-16CV	3.5112	2.79	0.1395	0.0698	1.99
DES-CP20	0.5599	4.84	0.2420	0.1210	21.61
Aq-CP20	3.5112	1.61	0.0805	0.0403	1.15
DES-100CD	0.5599	0.64	0.0320	0.0160	2.86
Aq-100CD	3.5112	0.75	0.0375	0.0188	0.53

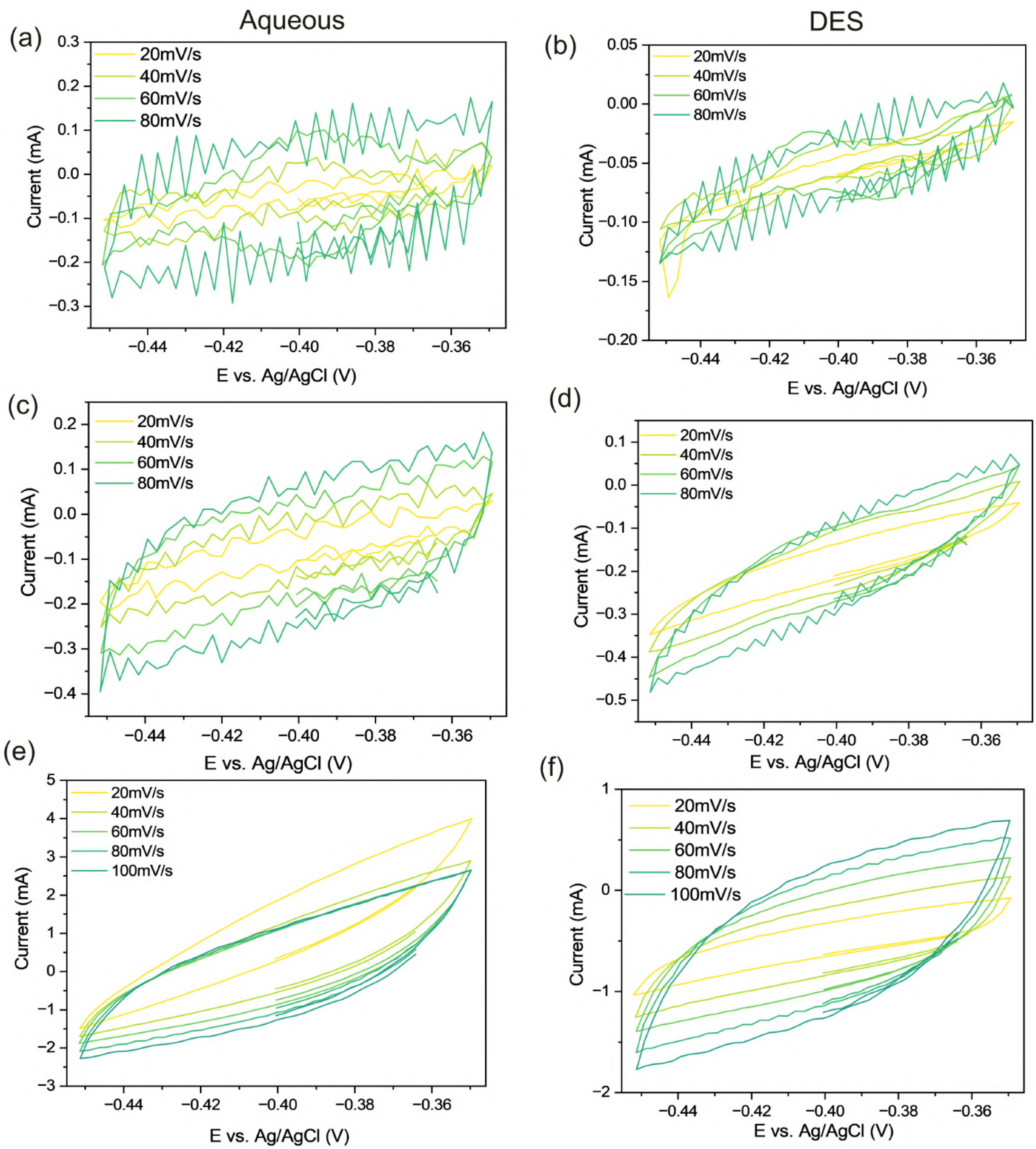

Fig. S26 Non-faradaic cyclic voltammograms for double-layer capacitance measurement. Cyclic voltammograms recorded in the non-faradaic potential window (−0.35 to −0.45 V vs. Ag/AgCl) at scan rates of 20, 40, 60, 80, and 100 mV s^{-1} in CO_2-saturated 0.5 M $KHCO_3$ after electrochemical activation. Left column (Aqueous): (a) 2 mA cm^{-2}, (c) 4 mA cm^{-2}, and (e) 18 mA cm^{-2} deposition current density. Right column (DES): (b) 2 mA cm^{-2}, (d) 4 mA cm^{-2}, and (f) 18 mA cm^{-2} deposition current density. The systematic increase in capacitive current magnitude with scan rate confirms a purely non-faradaic response across all samples. All measurements were performed after 16 CV activation cycles.

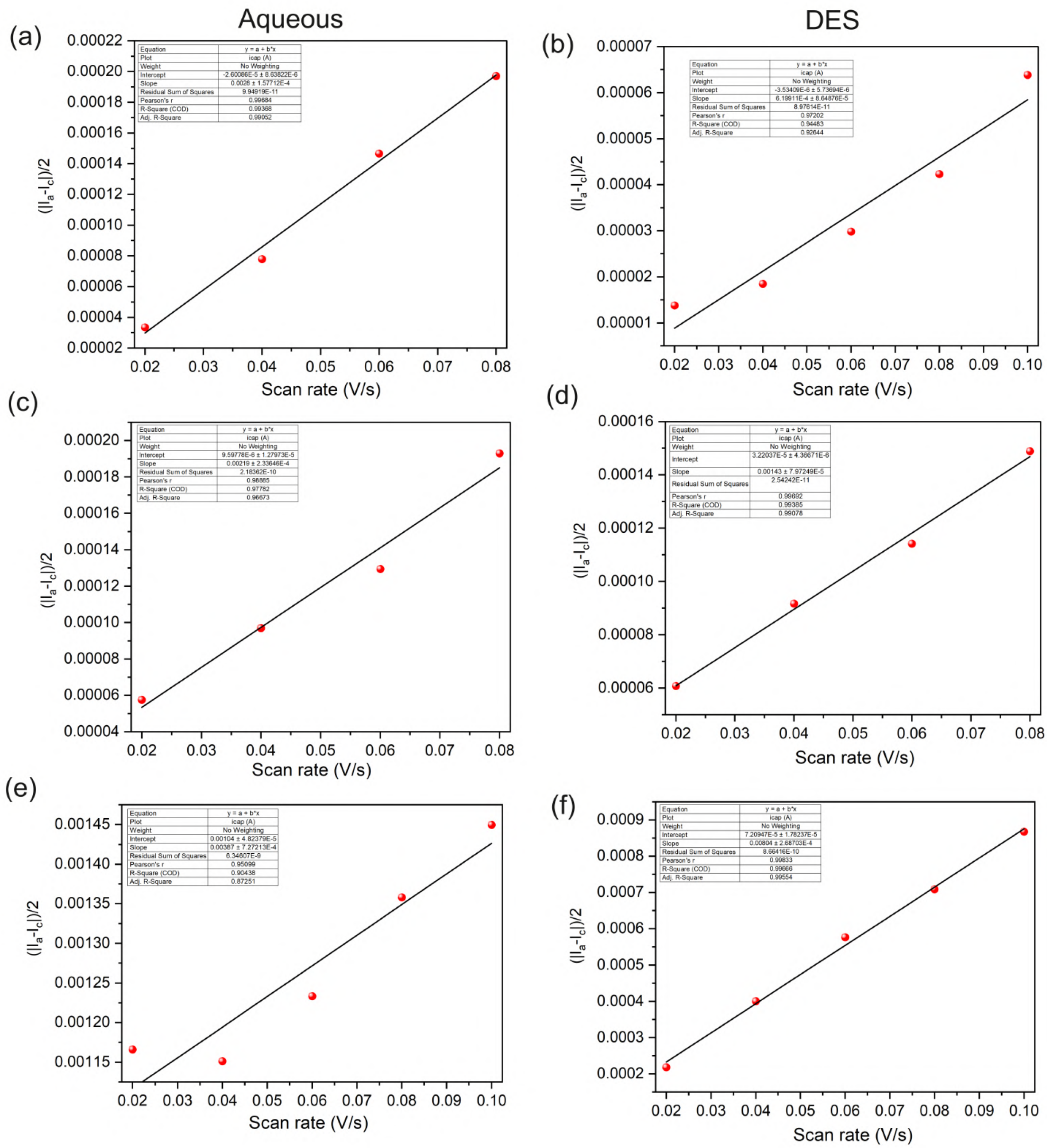

Fig. S27 Double-layer capacitance (C_{dl}) extraction from scan rate dependence. Plots of half the difference between anodic and cathodic current densities, $(|I_{\mathrm{a}} - I_{\mathrm{c}}|)/2$, as a function of scan rate for (a) Aq-2, (c) Aq-4, (e) Aq-18, (b) DES-2, (d) DES-4, and (f) DES-18. The slope of the linear fit corresponds to C_{dl} which is a relative descriptor of the electrochemically accessible interface.

Table S4 Crystallite size of pristine aqueous- and DES-derived Bi electrodes determined by Scherrer analysis of XRD reflections. Cu Kα radiation ($\lambda = 0.15406$ nm), $K = 0.9$.

Sample	Reflection	2θ ($^\circ$)	FWHM ($^\circ$)	β (rad)	D (nm)
Aqueous	(012)	27.22	0.1295	0.002260	63.1
	(104)	38.02	0.1591	0.002776	52.8
	(110)	39.68	0.1569	0.002738	53.8
				**Average**	**56.6**
DES	(012)	27.40	0.1397	0.002438	58.5
	(104)	38.19	0.1746	0.003047	48.2
	(110)	39.85	0.1665	0.002906	50.8
				**Average**	**52.5**

Table S5 XPS fitting results for DES- and aqueous-derived Bi electrodes: Bi $4f_{7/2}$ binding energies (BE) of Bi^0 and Bi^{3+}, Bi^0 fraction ($Bi^0/(Bi^0+Bi^{3+})$ from Bi 4f peak areas), and Bi–OH fraction ($Bi(OH)_3/(Bi_2O_3+Bi(OH)_3)$ from O 1s peak areas). Binding energies are referenced to the Fermi edge.

Sample	BE Bi^0 (eV)	BE Bi^{3+} (eV)	Bi^0 fraction (%)	Bi–OH fraction (%)
DES	156.84	159.58	20.1	30.9
DES 16CV	156.80	159.28	12.9	78.1
DES 100CD	156.80	159.15	7.6	70.1
Aq	156.97	159.27	4.9	35.0
Aq 16CV	156.96	159.49	11.2	72.9
Aq 100CD	156.85	159.24	9.5	68.5
DES post wash	156.80	159.41	4.5	70.0
Aq post wash	156.80	159.10	4.6	56.1

Table S6 Comparison of representative Bi-based catalysts for electrochemical CO_2 reduction to formate. The cell type is listed explicitly, as H-cell, flow-cell and zero-gap membrane electrode assembly (MEA) configurations impose different CO_2 mass-transport limits and are therefore not directly comparable. Catalyst loading is given where reported (n.r.: not reported).

Catalyst	Electrode preparation	Binder	Loading (mg cm^{-2})	Cell type	j (mA cm^{-2})	$FE_{formate}$ (%)	Stability	Ref.
Bi from DES (this work)	Direct electrodeposition	None	0.58	Flow cell (GDE)	100	96.8	32 h current-on (48 h elapsed)	**This work**
Commercial Bi powder (benchmark, same setup)	Air-brushed ink	Yes (Sustain-ion)	0.5	Flow cell (GDE)	100	6.2	—	**This work** (Fig. S13)
Bi_2O_3 nanofoam	Electrodeposition (DHBT) + annealing	None	n.r.	H-cell	—	>90 over ∼1.1 V window	—	Dutta 2021 (ACS Catal.)[1]
ap-Bi@GDE	Spray-coated ink	Yes	1.06	Flow cell (GDE)	111.6 (partial)	97.4	—	Zelocualtecatl-Montiel 2022 (ACS Catal.)[2]
OD-BiNSs	Spray-coated ink	Yes	∼1.0	Flow cell (GDE)	50–200	>94	—	Lee 2022 (ACS Appl. Mater. Interfaces)[3]
BiOBr-derived Bi	Drop-cast ink	Yes	n.r.	Flow cell (GDE)	148 (partial)	91	—	Yang 2023 (Nat. Catal.)[4]
BiO_n clusters	Catalyst ink	Yes	n.r.	MEA (zero-gap)	up to 500	>90	—	Jiang 2023 (Nano Res.)[5]
Co-doped Bi nanosheets	Air-brushed ink	Yes	0.4	Flow cell + solid electrolyte	200	∼90	100 h at 100 mA cm^{-2}	Nankya 2024 (Angew. Chem.)[6]
Bi/Bi_2O_3	Catalyst ink	Yes	67 ± 3	Flow cell (GDE)	150	∼90	21 h	Dinges 2025 (ChemSusChem)[7]
Cu-stabilised Bi subcarbonate	Catalyst ink	Yes	3.0	MEA (zero-gap, 100 cm^2)	300	92.5	130 h	Zhang 2025 (Nat. Commun.)[8]
Bi-GDE (VITO CORE)	Commercial GDE	—	n.r.	Flow cell (GDE, 400 cm^2)	100	63 (CE)	large-area operation	Izadi 2026 (Ind. Chem. Mater.)[9]

References

1. A. Dutta, I. Zelocualtecatl Montiel, K. Kiran, A. Rieder, V. Grozovski, L. Gut and P. Broekmann, *ACS Catal.*, 2021, **11**, 4988–5003.
2. I. Zelocualtecatl Montiel, A. Dutta, K. Kiran, A. Rieder, A. Iarchuk, S. Vesztergom, M. Mirolo, I. Martens, J. Drnec and P. Broekmann, *ACS Catal.*, 2022, **12**, 10872–10886.
3. J. Lee, H. Liu, Y. Chen and W. Li, *ACS Appl. Mater. Interfaces*, 2022, **14**, 14210–14217.
4. S. Yang, H. An, S. Arnouts, H. Wang, X. Yu, J. de Ruiter, S. Bals, T. Altantzis, B. M. Weckhuysen and W. van der Stam, *Nat. Catal.*, 2023, **6**, 796–806.
5. X. Jiang, L. Lin, Y. Rong, R. Li, Q. Jiang, Y. Yang and D. Gao, *Nano Res.*, 2023, **16**, 12050–12057.
6. R. Nankya, Y. Xu, A. Elgazzar, P. Zhu, T.-U. Wi, C. Qiu, Y. Feng, F. Che and H. Wang, *Angew. Chem. Int. Ed.*, 2024, **63**, e202403671.
7. I. Dinges, S. R. Waldvogel and M. Stöckl, *ChemSusChem*, 2025, **18**, e202501583.
8. H. Zhang, Z. Bo, M. Wang, W. Lin, Y. Liu, N. Liu, X. Sang, B. Yang, Z. Li, L. Lei, L. Dai and Y. Hou, *Nat. Commun.*, 2025, **16**, 577.
9. P. Izadi, S. Varhade, C. Schneider, P. Haus, C. Singh, A. Guruji, D. Pant and F. Harnisch, *Ind. Chem. Mater.*, 2026, **4**, 260.
10. J. Wu, X. Yu, H. He, C. Yang, D. Xia, L. Wang, J. Huang, N. Zhao, F. Tang, L. Deng and Y.-N. Liu, *Ind. Eng. Chem. Res.*, 2022, **61**, 12383–12391.
11. L. Lin, X. He, X.-G. Zhang, W. Ma, B. Zhang, D. Wei, S. Xie, Q. Zhang, X. Yi and Y. Wang, *Angew. Chem. Int. Ed.*, 2023, **62**, e202214959.
12. S. Yang, M. Jiang, W. Zhang, Y. Hu, J. Liang, Y. Wang, Z. Tie and Z. Jin, *Adv. Funct. Mater.*, 2023, **33**, 2301984.
13. B. Li, J. Chen, L. Wang, D. Xia, S. Mao, L. Xi, H. Liu, S. Ying and Y. Wang, *Adv. Sci.*, 2025, **12**, 2415616.
14. J. A. Steele and R. A. Lewis, *Opt. Mater. Express*, 2014, **4**, 2133–2142.
15. G. Jia, Y. Wang, M. Sun, H. Zhang, L. Li, Y. Shi, L. Zhang, X. Cui, T. W. B. Lo, B. Huang and J. C. Yu, *J. Am. Chem. Soc.*, 2023, **145**, 14133–14142.
16. D. Zhang, X. Liu, Y. Zhao, H. Zhang, A. V. Rudnev and J.-F. Li, *Chem. Sci.*, 2025, **16**, 4916–4936.
17. J. Jeon, H.-S. Bang, Y.-J. Ko, E. Huh, J. Kang, X. Zhang, S. Ka, Y. Kim, W. H. Lee, K. Kim, H. K. Yu, H.-S. Oh and J.-Y. Choi, *Chem. Eng. J.*, 2025, **523**, 168350.
18. T. Jawhari, A. Roid and J. Casado, *Carbon*, 1995, **33**, 1561–1565.
